# What do we know about UK household adaptation to climate change? A systematic review

**DOI:** 10.1007/s10584-014-1252-7

**Published:** 2014-09-18

**Authors:** James J. Porter, Suraje Dessai, Emma L. Tompkins

**Affiliations:** 1 Sustainability Research Institute and ESRC Centre for Climate Change Economics and Policy, School of Earth and Environment, University of Leeds, Leeds, LS2 9JT UK; 2 Geography & Environment, University of Southampton, (Highfield Campus), Southampton, SO17 1BJ UK

## Abstract

The UK Government’s first National Adaptation Programme seeks to create a ‘climate-ready society’ capable of making well-informed and far-sighted decisions to address risks and opportunities posed by a changing climate, where individual households are expected to adapt when it is in their interest to do so. How, and to what extent, households are able to do this remains unclear. Like other developed countries, research on UK adaptation has focused predominately on public and private organisations. To fill that gap, a systematic literature review was conducted to understand what actions UK households have taken in response to, or in anticipation of, a changing climate; what drives or impedes these actions; and whether households will act autonomously. We found that UK households struggle to build long-term adaptive capacity and are reliant upon traditional reactive coping responses. Of concern is that these coping responses are less effective for some climate risks (e.g. flooding); cost more over the long-term; and fail to create household capacity to adapt to other stresses. While low-cost, low-skill coping responses were already being implemented, the adoption of more permanent physical measures, behavioural changes, and acceptance of new responsibilities are unlikely to happen autonomously without further financial or government support. If public policy on household adaptation to climate change is to be better informed than more high-quality empirical research is urgently needed.

**Electronic supplementary material:**

The online version of this article (doi:10.1007/s10584-014-1252-7) contains supplementary material, which is available to authorized users.

## Introduction

Changes to the UK’s climate are unavoidable (Murphy et al. 2009). If the UK is to adapt, it needs to become a ‘climate-ready society’ capable of making ‘timely, far-sighted and well-informed decisions to address the risks and opportunities posed by a changing climate’ (Defra 2013: 11). That vision is neatly encapsulated in the UK Government’s first National Adaptation Programme (NAP). But it has very little to say about the role of households. This is somewhat surprising as households are places where adaptation happens. Households can take actions themselves to adapt, suffer the consequences of other’s actions such as through higher water bills, insurance premiums, or taxes, and exert pressure on local/national public policy to take further action as well as businesses. Besides these private benefits, household actions can also provide adaptation public goods including turning impermeable surfaces, like paved gardens, back into vegetated areas where runoff can be reduced after heavy downpours, for example (Tompkins and Eakin 2012). The NAP does state that ‘if adapting to climate change is in the private interests of an individual… then it should occur naturally and without the government’s intervention’ (Defra 2013: 7), placing the onus on individuals, and therein households, to get on with adapting themselves. How, and to what extent, households are able to do this remains unclear.

Home to 26 million households, the scale and extent of changes to the UK’s climate may not be realised for many decades but the UK’s sensitivity to climate-related risks: floods, heatwaves, cold spells, droughts, and water scarcity, is already being felt. Yet like other developed countries, research on UK adaptation has focused predominately on the actions of public and private organisations (Arnell and Delaney 2006; Berkhout et al. 2006; Tompkins et al. 2010). A systematic review of adaptation actions, by Ford et al. (2011) of academic literature published from 2006 to 09, found only three papers that documented household-level adaptation in developed nations, while UK activities dominated European adaptation publications. Since then, the UK government has increased its support for adaptation by passing the Climate Change Act 2008, funding climate information programmes under the UK Climate Impact Programme and then the Environment Agency’s Climate Ready Support Service for England, releasing new climate projections (Murphy et al. 2009), and delivering its first national Climate Change Risk Assessment (Defra 2012), all of which suggest that the UK continues to be a hive of climate change adaptation research activity (Mullan et al. 2013).

To understand what adaptation actions UK households have taken in response to, or in anticipation of, a changing climate, three main questions were asked: 1) What actions can households take to adapt? 2) What are the drivers of, or barriers to, household actions? and 3) Will households act autonomously? To answer these research questions, a systematic review of the peer-reviewed literature was conducted. Still relatively novel in climate change research, systematic reviews offer a promising method to identify, analyse, and synthesise large amounts of literature (see Berrang-Ford et al. 2011; Ford et al. 2011; Lorenz et al. 2014). Unlike traditional literature reviews, where the search or selection criteria are often unclear and rarely justified, systematic reviews are more transparent, accountable, and reproducible; and have been standard practice in the medical and public health sciences for several decades (e.g. Higgins and Green 2011; Moher et al. 2009; Morton et al. 2011).

## Data and methods

Following Berrang-Ford et al. (2011), a systematic review methodology was applied to assess what adaptation actions UK households have taken in response to, or in anticipation of, a changing climate. Using ISI Web of Knowledge, the largest and most comprehensive research publication database, a keyword search was made for journal articles on UK household adaptation actions published between January 2006 and November 2012. Papers published prior to 2006 were excluded as they were captured in the review process for the IPCC’s 2007 assessment report, although there was relatively little research on households adaptation (Alcamo et al. 2007).

As ‘climate change’ can manifest itself in various ways—change, variability, and extremes—different keyword combinations were used to capture the fullness of the topic. ‘Adaptation’ is similarly complex—encompassing risk, resilience and vulnerability, hence additional searches were run. In total, 138 keyword combinations including climat*, chang*, adapt*, household*, home*, resilien* and risk* were used (see Supplementary Materials for keyword list). One thousand two hundred thirty-five documents were returned. Once imported into the EndNote software, inclusion and exclusion criterion were applied.

Only empirical, peer-reviewed publications, written in English and focusing on present-day adaptive responses in the UK were included. Articles outside the scope of the study were excluded. That is, they examined adaptation in natural systems—biological responses, were conceptual, not peer-reviewed, only considered climate-mitigation, or focused on future impact studies (see Kent et al. 2013 for a review of the grey literature on UK household adaptation). Forty-eight UK-specific human adaptation papers were retained. To differentiate high-quality, empirically robust, publications from those using less rigorous research, these articles were graded from zero to five. Five star papers used methods highly appropriate for the research question at hand, were clearly executed, critically/statistically analysed, and covered a large sample size for the applied method. Surveys with over 200 subjects from a discrete population or in-depth interviews with more than 50 participants fitted into this bracket, for example (see Supplementary Materials).

Fifteen papers (1.2 % of the initial search) met the inclusion criteria, scoring three or above. An identifier number (#1–15) was assigned to each publication and is used to refer to each one individually (see Table 1). These 15 high-quality papers covered quantitative (*n* = 5), qualitative (*n* = 8), and mixed methods (*n* = 5) approaches, and focused on one or more of three main climate risks: heatwaves and management of heat stress (*n* = 7), cold spells and coping with winter extremes (*n* = 6), and flood risk and coastal erosion (*n* = 7).

**Table 1:** UK household papers systemically reviewed

Identifier	Paper details
1	Wolf J, Adger WN, Lorenzoni I, Abrahamson V, Raine R (2010) Social capital, individual responses to heat waves and climate change adaptation: An empirical study of two UK cities. Global Environmental Change-Human and Policy Dimensions 20:44–52.
2	Harvatt J, Petts J, Chilvers J (2011) Understanding householder responses to natural hazards: flooding and sea-level rise comparisons. Journal of Risk Research 14:63–83.
3	Bichard E, Kazmierczak A (2012) Are homeowners willing to adapt to and mitigate the effects of climate change? Climatic Change 112:633–654.
4	Harries T (2012) The anticipated emotional consequences of adaptive behaviour-impacts on the take-up of household flood-protection measures. Environment and Planning A 44:649–668.
5	Wolf J, Adger WN, Lorenzoni I (2010) Heat waves and cold spells: an analysis of policy response and perceptions of vulnerable populations in the UK. Environment and Planning A 42:2721–2734.
6	Glenk K, Fischer A (2010) Insurance, prevention or just wait and see? Public preferences for water management strategies in the context of climate change. Ecological Economics 69:2279–2291.
7	Hitchings R, Day R (2011) How older people relate to the private winter warmth practices of their peers and why we should be interested. Environment and Planning A 43:2452–2467.
8	Gupta R, Gregg M (2012) Using UK climate change projections to adapt existing English homes for a warming climate. Building and Environment 55:20–42.
9	Kazmierczak A, Cavan G (2011) Surface water flooding risk to urban communities: Analysis of vulnerability, hazard and exposure. Landscape and Urban Planning 103:185–197.
10	Brown S, Walker G (2008) Understanding heat wave vulnerability in nursing and residential homes. Building Research and Information 36:363–372.
11	Gul MS, Menzies GF (2012) Designing domestic buildings for future summers: Attitudes and opinions of building professionals. Energy Policy 45:752–761.
12	Oven KJ, Curtis SE, Reaney S, Riva M, Stewart MG, Ohlemuller R, Dunn CE, Nodwell S, Dominelli L, Holden R (2012) Climate change and health and social care: Defining future hazard, vulnerability and risk for infrastructure systems supporting older people’s health care in England. Applied Geography 33:16–24.
13	Williams K, Joynt JLR, Payne C, Hopkins D, Smith I (2012) The conditions for, and challenges of, adapting England’s suburbs for climate change. Building and Environment 55:131–140.
14	Bernier P, Fenner RA, Ainger C (2010) Assessing the sustainability merits of retrofitting existing homes. Proceedings of the Institution of Civil Engineers-Engineering Sustainability 163:197–207.
15	Jones CA, Davies SJ, Macdonald N (2012) Examining the social consequences of extreme weather: the outcomes of the 1946/1947 winter in upland Wales, UK. Climatic Change 113:35–53.

To ensure consistency, a qualitative scorecard was developed to record the characteristics of each article—authorship, research focus, methods used. Three core questions were posed: (1) What actions can a household take to adapt to climate change?, (2) What are the drivers, triggers, and barriers to these actions?, and (3) Will these actions happen autonomously? Close reading of the papers revealed a series of emergent themes related to the description of household adaptation allowing each paper to be assessed and compared.

## Results

### What actions can households take to adapt?

Household responses have been grouped into two main types: intuitive, inexpensive, and accessible responses (which we term ‘*coping responses’*) and more complex, costly, and challenging anticipatory actions (which we term ‘*adaptations’*). Greater personal, financial, and technical investment is required in the latter. No judgement is made about the efficacy of the action taken. Significantly hotter or colder weather was associated with reactive coping responses whereas the threat of flooding involved more proactive adaptations.

Coping responses dominated heat-stress management (six of the seven papers, strongly agreed, #1, 5, 8, 10, 12, 13). Changing one’s clothes, dietary intake, and keeping windows open at night, are all low cost, low tech, and quickly implementable fixes (#1, 5, 10) (see Table 2 for greater detail). In contrast, less evidence was found of adaptations such as landscaping efforts to introduce shaded areas into gardens or modifications made to existing properties to accommodate air-conditioning (#11, 13).

**Table 2:** Examples of UK household actions as a function of climate risk (heatwaves, coldspells and flooding) and type of action (coping response, adapting to current and potential risks)

	Coping responses	Adapting to current risks	Adapting to potential risks
Heatwaves	• Take regular cool showers, baths or body washes • Seek shade outside the home • Change dietary intake (e.g. salads) • Keep windows open at night to aid natural ventilation • Wear less and lighter clothing	• Purchase lockable limiters on windows to allow them to remain open yet secure at night • Install ceiling fans in bedrooms • Erect canopies for shade	• Install air-conditioning /active cooling units • Paint external walls white or fitting a reflective roof to increase albedo • Purchase external shutters • Plant trees and plants for shade
Cold spells	• Wear extra and heavier clothing • Change dietary intake (e.g. hot meals and drinks) • Turn up, or keep on for longer, heating	• Replace single or cold glazed windows with new double glazing • Install cavity-wall and loft insulation for heat retention • Purchase draft-proofing measures to window seals and external facing doors • Use the Government’s winter fuel allowance (>65 years)	• Relocate away from areas that can become isolated or cut off during heavy snowfall
Flooding	• Turn off gas water and electricity mains • Move valuables and sentimental items upstairs	• Use doorguards. Gates, and toilet plugs to prevent water getting in • Seal entry points, water/sewage and electricity pipes • Waterproof external walls, doors • Raise thresholds, floors • Subscribe to the Environment Agency’s flood warning service • Take out appropriate flood insurance policies	• Move electricity fixtures up the wall • Tiling/water resistant paint for lower ground floor areas • Relocate white goods above flood levels • Removal of non-porous surfaces (e.g. driveways) • Plant vegetation rich spaces

Source: Kent et al. (2013: 54/55)

A similar array of low cost, low-tech, responses were found for minimising exposure to cold temperatures including changing clothes, diets, and routines (five of the six papers, strongly agreed, #3, 5, 7, 12, 14). Rather than keeping homes warm all day, older people innovated by visiting friends/families or community venues where warmth was guaranteed. Some evidence of more demanding and costly alternatives included installing double-glazed windows, cavity/loft insulation, and efficient boilers (#3, 12, 14). Flood risk responses, in contrast to heat-stress and cold extremes, required more high tech, complex responses (five of the seven papers, strongly agreed, #2, 3, 4, 9, 13). Reinstating porous surfaces (#9), introducing resilience measures by moving electricity fixtures up the wall or replacing carpeted areas with tiled floors (#3, 8), and acquiring specialized insurance were popular choices but required forward planning and investment (#2, 6).

### What are the drivers of, or barriers to, household adaptation?

Three main drivers influenced responses to all climate risks: previous exposure to weather extremes, pressure of social acceptability, and long-term financial rewards. Consciously or not, these drivers can spur on action as well as influence what actions are taken. For example, in colder weather older people may prefer to wear extra clothing or sit with blankets around them (#5, 7, 13, 14). Yet when hosting friends/family older people may not want to concern visitors that they are cold or may want to provide a warm environment. Turning up the heating in these situations is often the only socially acceptable option.

Barriers to household adaptation are more risk specific. The literature indicates that uptake of strategies for minimising cold temperature exposure (e.g. double-glazing and cavity/loft insulation) is based on calculations about when, or by how much, these installations will provide financial returns - lowering fuel bills or providing protection from rising energy costs (#5, 13, 14). Other barriers included concern over: (i) finding qualified and reliable professionals, and (ii) home-life disruption whilst the work is done (#5, 13, 14) . Slow cost recovery, workmanship concerns, and installation disruption are also barriers for responding to heat stress. Air-conditioning for an average size property can cost around £10,000 to install and thereafter incur operational and maintenance costs (#1, 8, 11). Aesthetic and security considerations present further barriers. Fitting air-conditioning can involve modifying window frames to accommodate units and therein change the property’s façade. If located in a conservation area, social and legal obstacles can exist (#13). Modified window frames can also attract burglars, while leaving windows open at night is a safety hazard for small children and vulnerable groups (#1, 8, 10).

In response to flood risk, households appear to have two main aims: to preserve an appreciating asset and to avoid exposure to floodwaters. Insurers and lenders can play a key role in incentivising homeowner action, either through the withdrawal of insurance cover/increasing premiums and excesses, or lenders declining mortgage applications (#2, 3, 4, 6). Homeownership, paradoxically, can act both as a driver and a barrier to responding to flood risk. Areas of mixed types of ownership—rental, owner-occupied, and social housing—experienced lower levels of flood management actions (#2, 3, 4, 13). Unable to afford or lacking the authority to make alterations to the building, tenants can be left exposed. Even when flood risk reducing investments are made, if insurers fail to reward homeowners with lower premiums/excesses, the financial payoff acting as an incentive for further action is lost (#2, 3, 6, 9). Personal experience of flooding can also be a barrier to action. Unwilling to accept their home as unsafe, anxiety avoidance—an emotional response affecting an individual’s perception of responsibility and capacity to act—can be encountered (#4).

### Will household act autonomously?

Households are already undertaking low-cost, low-skill, coping responses to hot and cold weather by changing dietary intake, clothing, and routines. Flood-proofing measures rolled out quickly and temporarily—doorguards, toilet plugs, and airbrick covers—have also been implemented (five of the seven papers, agreed, #2, 3, 4, 9, 13). This suggests that low-level coping can occur without government intervention (four of the nine papers, strongly agreed, #1, 5, 8, 13). More permanent measures—tiling floors, relocating electrical fittings, and removing impermeable surfaces—are expected to be adopted only if financial incentives are offered from government or insurers (#2, 3, 4, 9). Autonomous actions involving behavioural changes, acceptance of new responsibilities, or extensive-technical/resource-intensive actions are very unlikely to occur, however (eight of the nine papers, strongly agreed, #1, 2, 3, 4, 5, 8, 9, 13).

The role of the state is unclear in motivating these actions. Those at risk of flooding tend to defer responsibility to government; believe the insurance safety-net will save them; and cite unfamiliarity with flood-proofing products as reasons for not taking action (five of the seven papers strongly agreed, #2, 3, 4, 9, 13). Yet lacking access to state provided resources to support anticipatory adaptations does not always appear to be a barrier to action (eight of the nine papers strongly agreed, #1, 2, 3, 4, 5, 8, 9, 13). Less than a third of government grants for flood-proofing goods and home-insulation have been taken up, for example (#4, 14) . While in relation to over-heating, a few households were found to have taken expensive adaptation decisions (e.g. installing air-conditioning units, interior redesign, and reflective roofs), despite the lack of public funds available to support these actions (#1, 5).

## Discussions and conclusion

Our systematic review of the academic literature shows that the evidence-base for household adaptation in developed countries, particularly the UK, has grown steadily since 2006 (see Ford et al. 2011). Yet still with only 15 high-quality empirical papers found, our ability to quantitatively interrogate the literature for emergent patterns is limited. This importantly points to the significant gap in the research base, which will need to be filled if discussions on how, or to what extent, households are able and willing to adapt, are to be better informed. Of those papers reviewed, three key findings stand out.

First, UK households routinely take small, low cost, low tech, intuitive, and quickly implementable actions such as changing diets, clothing, and opening/closing windows, as coping responses to existing climatic variability. More proactive adaptations to larger, systematic, climatic changes involving greater personal, financial, and technical investment including the installation of air-conditioning or porous surfaces were rarely found. Although short-term, ‘pay-as-you-go’, coping responses are voluntarily adopted they are often risk specific (e.g. cold-snaps, heat-waves) and temporary, many of which fail to generate longer-term capacity to adapt. This leads us to question the role of existing government schemes in promoting anticipatory adaptation. It is interesting to note that much of the research on household adaptation to climate change has been conducted in developing countries with a different livelihood context (see Hisali et al. 2011 on Uganda’s argicultural production). The lack of a comparable body of literature in the UK makes any meaningful comparisons with our results difficult.

Second, past exposure to extreme weather, pressures of social acceptability, and long-term financial rewards appear to be the main drivers of household adaptation. Extreme weather events/impacts and recognising opportunities have been identified in other work as key drivers of adaptation, for example, in the development of National Adaptation Strategies in Europe (Biesbroek et al. 2010), and in driving institutional adaptation action in the UK (Tompkins et al. 2010). Our findings showed that barriers to action were risk-specific, but included financial considerations as well as a preference for convenience and personal exposure to climate risks. While there is a burgeoning theoretical literature on barriers to adaptation (Adger et al. 2009; Biesbroek et al. 2010; Dow et al. 2013; Klein et al. 2014), it has been difficult to draw out general findings from the empirical evidence due to the small number of case studies, where context and scale are critical.

Lastly, our findings suggest that long-term household adaptations are unlikely to happen autonomously. Short-term and low-cost coping responses are already being undertaken but more involved adaptations that require time and investment need incentives if they are to be adopted. These results indicate that new initiatives may be needed from the state or the private sector (e.g., insurers) to encourage long-term household adaptation. Behavioural changes and acceptance of new responsibilities are also unlikely to occur autonomously. While there is a growing research focus on transformational change, which describes shifts in social values, institutions, and technical practices (Pelling 2011), our research suggests that in relation to the UK’s National Adaptation Programme neither progressive adaptation nor transformational change/adaptation is likely to happen without state support. This could include demonstrating the financial rewards that householders, and society more widely, will reap when adaptations are taken early or rethinking what is socially acceptable through the introduction of new building standards to cope with extreme weather. Making social contracts and their renegotiations more explicit has been put forward as one way to facilitate autonomous adaptation and enable transformation (Adger et al. 2013), however there is a lack of empirical evidence to back this up.

If the UK Government is serious about evidence-based policymaking then much more high-quality empirical research is urgently needed on household adaptation to aid climate adaptation policy-making.

## Electronic supplementary material

Below is the link to the electronic supplementary material.

ESM 1 (DOC 461 kb)
