# Supplementary material for: What do we know about UK household adaptation to climate change? A systematic review
Source: Clim Change. 2014 Sep 18;127(2):371–9. doi: 10.1007/s10584-014-1252-7 (PMC4372777; doi:10.1007/s10584-014-1252-7)
Supplement: Supplementary file 1 — (DOC 461 kb) [file 10584_2014_1252_MOESM1_ESM.doc]

**Supplementary Materials**

**Table of Contents**

1. Methodology for Selecting Peer-Reviewed Publications [1](#__RefHeading___Toc266692095)

2. Selection Criteria for Systematic Evidence Review [2](#__RefHeading___Toc266692096)

3. Ranking Criteria and Examples [3](#__RefHeading___Toc266692097)

3. Example of Scorecard for Recording Article Characteristics [6](#__RefHeading___Toc266692099)

4. Excluded Papers by Category (n=1187) [8](#__RefHeading___Toc266692100)

# 1. Methodology for Selecting Peer-Reviewed Publications

To ensure consistency, and defensibility, across the systematic literature review three interrelated stages were involved.

1. After selecting a consistent set of keywords, ISI Web of Knowledge was searched for peer review articles, between 1^st^ January 2006 and 1^st^ November 2012, corresponding to climate adaptation on an individual/household level.
2. A filtering framework was then applied to prioritise *only* UK-based empirical papers, and to differentiate the high-quality publications from less rigorous research; and
3. Finally, to structure the analysis a set of four core research questions were used to introduce consistency across the reviews: What actions are households taking to adapt to climate change? What are the drivers, triggers, and barriers to action? How effective are these actions? Will these actions happen autonomously?

Two searches were performed and the results of each were combined (see below). As climate change can manifest itself in a variety of ways, not just ‘change’ *per se*, to capture the fullness of the subject, and to gain an understanding ‘to what’ people are responding, different keyword variations were used.

**Search 1 – Variations of Climate Risk for Individuals/Households etc**

Level 1 Climat*

PLUS ONE OF THE FOLLOWING

Level 2 Chang* Variab * Extreme*

PLUS ONE OF THE FOLLOWING

Level 3 Resilien* Vulnerab* Risk* Adapt*

PLUS ONE OF THE FOLLOWING

Level 4 Individual* Household* Community* Group* Family* Home*

PLUS ONE OF THE FOLLOWING

Level 5 Cost* Economic*

**Search 2 – Substitutions for Risk, Resilience, Adaptation and Vulnerability**

Level 1   climate*

Level 2   chang*

PLUS ONE OF THE FOLLOWING

Level 3

| robust | poverty | preparedness | knowledge |
| --- | --- | --- | --- |
| panarchy | social safety net | disaster mitigat* | inform* |
| renew* | poor | disaster risk | tool* |
| learn* | well-being | hazard manage* | technolog* |
| self-organis* | coping | disaster response* | skill* |
| buffer* | cope | disaster recovery* | behaviour |
|  |  | recovery* | financ* |
|  |  | expos* | social network* |
|  |  | sensitiv* | social capital* |
|  |  | perception | capacity |
|  |  |  | ability |

PLUS ONE OF THE FOLLOWING

Level 4    Individual*      Household*

Level 5    UK

The first search involved 72 different keyword combinations and delivered 1102 peer-reviewed publications. Using Endnote, these papers were filtered in relation to UK-specificity (cf. Great Britain, England, Northern Ireland, Scotland and Wales). The second search involved 66 keyword combinations and returned an additional 133 papers. After combining the two samples, and applying the selection criteria (see below), 48 UK-specific papers were prioritised. That subset yielded 15 peer-reviewed publications, which met the 3* or above ranking and was put forward for full analysis.

# 2. Selection Criteria for Systematic Evidence Review

- **Content**. Only *UK-based Research Papers* were analysed. Review articles and commentaries were excluded. That is, *empirically grounded research* is prioritized over conceptual work.
- **Methods.** As part of assessing the empirical fit of the methods used, priority was given to established quantitative and qualitative techniques. If a survey was used, what was the response rate? How appropriate was the target audience? Is it representative? What was asked? And what statistical techniques were used in the analysis (different methods have different levels of sophistication)? The same was asked of interview data. How consistent, or comparable, is the sample? Is the material collected exhaustive (triangulated with other textual sources etc) or convenient (small set of interviews)? And have proven analytical techniques been applied (content/discourse analysis)?
- **Scalability**. The capacity to *scale up* findings, whether they are from surveys or case study driven, from one location to other parts of the UK was key. Has a discrete sub-population been targeted (cf. older people, children, rural communities)? And could the methods/data be extended beyond it’s specific research site without distortion?

Inclusion/Exclusion Criteria for Selecting Documents

| PHASE 1: Keyword Search | |
| --- | --- |
| **Inclusion Criteria** | **Exclusion Criteria** |
| - English Language - 1^st^ January 2006 and 1^st^ November 2012 - Indexed in ISI Web of Knowledge - Peer-reviewed publications | - Non-English Language - Pre-2006 and after 1^st^ November 2012 - Neither indexed nor available from ISI Web of Knowledge - Others (editorials, reviews, book chapters, meetings etc) |

| PHASE 2: Title and Abstract Review | |
| --- | --- |
| **Inclusion Criteria** | **Exclusion Criteria** |
| - Adaptation in human systems - Adaptive response(s) - Practical/empirical focus - Fieldwork conducted after 2000. - UK-based | - Adaptation in natural systems (cf. plants, animals, microbes) - Mitigation only - Conceptual focus only - Prehistoric or future - Non-UK |

# 3. Ranking Criteria and Examples

| **Star Rating** | **Assessment** | **Example** |
| --- | --- | --- |
| 5* | - **Methods** used are highly appropriate for the research at hand, clearly executed and critically justified, and cover a sufficiently large sample size. Questionnaires that survey over 200 subjects of a discrete population, or in-depth interviews with more than 50 participants would fit into this bracket, for example. Additionally, the application of multiple methods to provide triangulation is another mark of the highest quality evidence. - Choice of research site, question(s) explored, and presentation offer immediate **scalability**. Findings can be both scaled up from local to regional and even national scales and scaled back again. - Outlook of the research speaks directly to, and actually develops, the questions posed in the systematic literature review. | Wolf et al (2012) |
| 4* | - **Methods** used are appropriate for the research at hand, clearly presented and justified. Questionnaires that survey less than 200 subjects of a discrete population, or several, and in-depth interviews with less than 50 respondents would fit into this bracket, for example. Application of triangulation, even with lower sample sizes, is a good indication of high quality evidence. - Findings, either in the form of the research site chosen or questions asked, can more-or-less be **scaled up** to a number of places in the UK without much distortion. - Themes covered by the research are closely related to the questions posed in the systematic literature review. | Kazmierczak and Cavan (2012) |
| 3* | - **Methods** used are appropriate for the research at hand, and well justified, and the sample sizes are sufficient. Questionnaires that survey less than 50 subjects of a discrete population and in-depth interviews with less than 25 respondents fit into this bracket, for example. - Findings, either in the form of the research site chosen or questions asked, can more-or-less be **scaled up** to other places in the UK with the caveat that their applicability will vary. - Themes covered by the research are closely related to the questions posed in the systematic literature review. | Brown and Walker (2008) |

Figure 1: Document Selection Process

# 3. Example of Scorecard for Recording Article Characteristics

| **Ref [1] JP** | **[Wolf et al 2012]** |
| --- | --- |
| **Title** | **Social capital, individual responses to heat waves and climate change adaptation: An empirical study of two UK cities** |
| **Review rating** | Five star. |
| **Climate hazard (s) covered** | Heat waves |
| **Climate risks or opportunities covered** | Vulnerability of elderly people |
| **Overview** | It has been claimed that high social capital contributes to both positive public health outcomes and to climate change adaptation. Strong social networks have been said to support individuals and collective initiatives of adaptation and enhance resilience. As a result, there is an expectation that social capital could reduce vulnerability to risks from the impacts of climate change in the health sector. This paper examines evidence on the role social networks play in individuals' responses to heat wave risk in a case study in the UK. Based on interviews with independently living elderly people and their primary social contacts in London and Norwich, we suggest that strong bonding networks could potentially exacerbate rather than reduce the vulnerability of elderly people to the effects of heat waves. Most respondents interviewed did not feel that heat waves posed a significant risk to them personally, and most said that they would be able to cope with hot weather. Bonding networks could perpetuate rather than challenge these narratives and therefore contribute to vulnerability rather than ameliorating it. These results suggest a complex rather than uniformly positive relationship between social capital, health and adaptation to climate change. |
| **Methods:** | **What methodological/empirical criteria does the paper meet?** |
|  | Focusing on the role played by older people’s perceptions of vulnerability and heat risk, the study involved semi-structured (n=105) with people aged 75 or above at two locations: Norwich and London, to collect data. Consistently in approach was introduced through an interview protocol. London was chosen because it was the worst affected city in the 2003 heat wave and Norwich acted as another city to complemented that. Respondents (n=60) were selected on the basis of Index of Multiple Deprivation of postcodes and the other (n=45) through social contacts, via General Practice databases. Grounded theory, aided by NVivo, was used to analysis the data. All of this points to a **very strong**, **rigourous** and **empirically-grounded** research project. |
| **Scalability:** | **Could the data/findings collected by scaled up to a national-scale?** |
|  | Certain aspects of the study lent themselves better to be **scaled up** than others. The inclusion of a capital city and one major city, and the large number of respondents, means the results should be applicable to other parts of the UK with large urban areas. How transferable the findings are for rural and semi-rural areas is not as clear. |
| **Question 1:** | **What actions can an individual/household take to adapt to climate change?** |
| **Response 1** | Older individuals/households, living independently without the assistance of a carer, had a number of **actions** available to them but the study focused mainly on immediate heat abatement strategies. For example, taking a cool shower, increasing the amount of water consumed, staying out of direct sunlight, and delaying activities such as shopping etc to cooler times of the day. If residents felt unable to cope they would contact friends/family for help. |
| **Question 2:** | **What are the drivers, triggers, and barriers to these actions?** |
| **Response 2** | The paper suggests that the main **driver** for action was an unplanned response to intense heat. Friends/family acted as an important factor in helping older individuals/households to cope with extreme heat events. A potential **barrier**, however, was knowledge about the effect heat risks on the health of older people varied considerably. An unwillingness to report problems, and be seen as a burden to friends/family or the health service, meant they do not always seek help when experiencing heat-related side effects and potentially exacerbated the seriousness of the problem. Many respondents did not perceive themselves to be at-risk from heat illness and had not made any plans to cope with such an event. As a result, some key risks missed by family/friends such as heat stress can go untreated. Here the seriousness and awareness to put plans in place of heat risks can act as a **barrier**. |
| **Question 3:** | **How effective are these actions?** |
| **Response 3** | It is argued in the paper that the **effectiveness** of those actions is often compromised because remedial measures are taken too late. Such actions are, therefore, only **effective** is taken early on and followed consistently during a heat wave. Dehydration is not always easy to identify, especially for family/friends who pop in and out during the day, and of course, the exposure from prolonged heat overnight. Implicit throughout the paper, it is assumed, these measures can be **effective** is implemented correctly. |
| **Question 4:** | **Will these actions happen autonomously?** |
| **Response 4** | The authors suggest that there is a tension underlying this case study between the vulnerability of older individuals/householders and a need to be seen as independent, which impact on his/her perception of heat risk. Whilst there is evidence that those aged 75 or above are greater risk from heat, this seems to be at odds with commonsense perceptions of resilience. As a result, whilst these actions may happen **autonomously** persistent concerns over a lack of awareness or the necessity to take action means that more communication is needed from central/local government to convey not only the risks but what individuals/households can do about them |
| **Full reference** | Wolf, J., Adger, N., Lorenzoni, I., Abrahmson, V. and Raine, R. 2010. Social capital, individual responses to heat waves and climate change adaptation: An empirical study of two UK cities. *Global Environmental Change*, 20, 44-52. |

# 4. Excluded Papers by Category (n=1187)

**Non-UK Specific (n=524)**

1. Abuodha, P.A.O. and C.D. Woodroffe, Assessing vulnerability to sea-level rise using a coastal sensitivity index: a case study from southeast Australia. Journal of Coastal Conservation, 2010. 14(3): p. 189-205.

2. Acosta-Michlik, L. and V. Espaldon, Assessing vulnerability of selected farming communities in the Philippines based on a behavioural model of agent's adaptation to global environmental change. Global Environmental Change-Human and Policy Dimensions, 2008. 18(4): p. 554-563.

3. Adanalyan, A. and S. Gevorgyan, The Global Climate Change Impact on Water Resources of Armenia, in Climate Change and Its Effects on Water Resources: Issues of National and Global Security, A. Baba, et al., Editors. 2011. p. 123-129.

4. Addo, K.A., et al., Impacts of Coastal Inundation Due to Climate Change in a CLUSTER of Urban Coastal Communities in Ghana, West Africa. Remote Sensing, 2011. 3(9): p. 2029-2050.

5. Aerts, J.C.J.H., et al., Dealing with Uncertainty in Flood Management Through Diversification. Ecology and Society, 2008. 13(1).

6. Aerts, J.C.J.H. and W.J.W. Botzen, Flood-Resilient Waterfront Development in New York City: Bridging Flood Insurance, Building Codes, and Flood Zoning, in Flood-Resilient Waterfront Development in New York City: Bridging Flood Insurance, Building Codes, and Flood Zoning. 2011. p. 1-82.

7. Ahammad, R., Constraints of pro-poor climate change adaptation in Chittagong city. Environment and Urbanization, 2011. 23(2): p. 503-515.

8. Ahmed, S.A., N.S. Diffenbaugh, and T.W. Hertel, Climate volatility deepens poverty vulnerability in developing countries. Environmental Research Letters, 2009. 4(3).

9. Akter, S., The Role of Microinsurance as a Safety Net Against Environmental Risks in Bangladesh. Journal of Environment & Development, 2012. 21(2): p. 263-280.

10. Al-Jeneid, S., et al., Vulnerability assessment and adaptation to the impacts of sea level rise on the Kingdom of Bahrain. Mitigation and Adaptation Strategies for Global Change, 2008. 13(1): p. 87-104.

11. Alam, E. and A.E. Collins, Cyclone disaster vulnerability and response experiences in coastal Bangladesh. Disasters, 2010. 34(4): p. 931-954.

12. Alayon-Gamboa, J.A. and J.C. Ku-Vera, Vulnerability of smallholder agriculture in Calakmul, Campeche, Mexico. Indian Journal of Traditional Knowledge, 2011. 10(1): p. 125-132.

13. Albano Amora, S.S., et al., Monitoring of Lutzomyia longipalpis Lutz & Neiva, 1912 in an area of intense transmission of visceral leishmaniasis in Rio Grande do Norte, Northeast Brazil. Revista Brasileira De Parasitologia Veterinaria, 2010. 19(1): p. 39-43.

14. Alberini, A. and A. Chiabai, Urban environmental health and sensitive populations: How much are the Italians willing to pay to reduce their risks? Regional Science and Urban Economics, 2007. 37(2): p. 239-258.

15. Alessa, L., et al., Perception of change in freshwater in remote resource-dependent Arctic communities. Global Environmental Change-Human and Policy Dimensions, 2008. 18(1): p. 153-164.

16. Alexander, K.S., A. Ryan, and T.G. Measham, Managed retreat of coastal communities: understanding responses to projected sea level rise. Journal of Environmental Planning and Management, 2012. 55(4): p. 409-433.

17. Alexandra, J., Australia's landscapes in a changing climate-caution, hope, inspiration, and transformation. Crop & Pasture Science, 2012. 63(3): p. 215-231.

18. Allen, K.M., Community-based disaster preparedness and climate adaptation: local capacity-building in the Philippines. Disasters, 2006. 30(1): p. 81-101.

19. Altaweel, M.R., L.N. Alessa, and A.D. Kliskey, Cadillac Desert: The American West and Its Disappearing Water. Journal of the American Water Resources Association, 2009. 45(6): p. 1379-1389.

20. Andersson, L., et al., Participatory modelling for locally proposed climate change adaptation related to water and agriculture in South Africa, in Global Change: Facing Risks and Threats to Water Resources, E. Servat, et al., Editors. 2010. p. 214-220.

21. Angadi, S.V., et al., Adaptation of alternative pulse and oilseed crops to the semiarid Canadian Prairie: Seed yield and water use efficiency. Canadian Journal of Plant Science, 2008. 88(3): p. 425-438.

22. Antwi-Agyei, P., et al., Mapping the vulnerability of crop production to drought in Ghana using rainfall, yield and socioeconomic data. Applied Geography, 2012. 32(2): p. 324-334.

23. Appiah, M., et al., Forest and agroecosystem fire management in Ghana. Mitigation and Adaptation Strategies for Global Change, 2010. 15(6): p. 551-570.

24. Archer, E., et al., Sustaining agricultural production and food security in Southern Africa: an improved role for climate prediction? Climatic Change, 2007. 83(3): p. 287-300.

25. Ayers, J., International funding to support urban adaptation to climate change. Environment and Urbanization, 2009. 21(1): p. 225-240.

26. Ayers, J., Resolving the Adaptation Paradox: Exploring the Potential for Deliberative Adaptation Policy-Making in Bangladesh. Global Environmental Politics, 2011. 11(1): p. 62-+.

27. Ayers, J. and T. Forsyth, COMMUNITY-BASED ADAPTATION TO CLIMATE CHANGE: Strengthening Resilience through Development. Environment, 2009. 51(4): p. 22-31.

28. Badjeck, M.-C., et al., Impacts of climate variability and change on fishery-based livelihoods. Marine Policy, 2010. 34(3): p. 375-383.

29. Badjeck, M.-C., et al., Climate variability and the Peruvian scallop fishery: the role of formal institutions in resilience building. Climatic Change, 2009. 94(1-2): p. 211-232.

30. Bajayo, R., Building community resilience to climate change through public health planning. Health Promotion Journal of Australia, 2012. 23(1): p. 30-36.

31. Bambrick, H.J., et al., Climate Change and Health in the Urban Environment: Adaptation Opportunities in Australian Cities. Asia-Pacific Journal of Public Health, 2011. 23(2): p. 67S-79S.

32. Banerji, G. and S. Basu, Adapting to climate change in Himalayan cold deserts. International Journal of Climate Change Strategies and Management, 2010. 2(4): p. 426-448.

33. Barbier, B., et al., Human Vulnerability to Climate Variability in the Sahel: Farmers' Adaptation Strategies in Northern Burkina Faso. Environmental Management, 2009. 43(5): p. 790-803.

34. Barbieri, A.F., et al., Climate change and population migration in Brazil's Northeast: scenarios for 2025-2050. Population and Environment, 2010. 31(5): p. 344-370.

35. Bardsley, D.K. and G.P. Rogers, Prioritizing Engagement for Sustainable Adaptation to Climate Change: An Example from Natural Resource Management in South Australia. Society & Natural Resources, 2011. 24(1): p. 1-17.

36. Bardsley, D.K. and S.M. Sweeney, Guiding Climate Change Adaptation Within Vulnerable Natural Resource Management Systems. Environmental Management, 2010. 45(5): p. 1127-1141.

37. Barnett, J., Dangerous climate change in the Pacific Islands: food production and food security. Regional Environmental Change, 2011. 11: p. S229-S237.

38. Bartlett, S., Climate change and urban children: impacts and implications for adaptation in low- and middle-income countries. Environment and Urbanization, 2008. 20(2): p. 501-519.

39. Battaglini, A., et al., European winegrowers' perceptions of climate change impact and options for adaptation. Regional Environmental Change, 2009. 9(2): p. 61-73.

40. Bayramin, I., G. Erpul, and H.E. Erdogan, Use of CORINE methodology to assess soil erosion risk in the semi-arid area of Beypazari, Ankara. Turkish Journal of Agriculture and Forestry, 2006. 30(2): p. 81-100.

41. Beaumier, M.C. and J.D. Ford, Food Insecurity among Inuit Women Exacerbated by Socio-economic Stresses and Climate Change. Canadian Journal of Public Health-Revue Canadienne De Sante Publique, 2010. 101(3): p. 196-201.

42. Beckman, M., Converging and conflicting interests in adaptation to environmental change in central Vietnam. Climate and Development, 2011. 3(1): p. 32-41.

43. Bedsworth, L., Air quality planning in California's changing climate. Climatic Change, 2012. 111(1): p. 101-118.

44. Beier, C., et al., Precipitation manipulation experiments - challenges and recommendations for the future. Ecology Letters, 2012. 15(8): p. 899-911.

45. Belanger, D., et al., Use of a Remote Car Starter in Relation to Smog and Climate Change Perceptions: A Population Survey in Quebec (Canada). International Journal of Environmental Research and Public Health, 2009. 6(2): p. 694-709.

46. Belliveau, S., B. Smit, and B. Bradshaw, Multiple exposures and dynamic vulnerability: Evidence from the grape industry in the Okanagan Valley, Canada. Global Environmental Change-Human and Policy Dimensions, 2006. 16(4): p. 364-378.

47. Bellon, M.R., D. Hodson, and J. Hellin, Assessing the vulnerability of traditional maize seed systems in Mexico to climate change. Proceedings of the National Academy of Sciences of the United States of America, 2011. 108(33): p. 13432-13437.

48. Bergsma, E., J. Gupta, and P. Jong, Does individual responsibility increase the adaptive capacity of society? The case of local water management in the Netherlands. Resources Conservation and Recycling, 2012. 64: p. 13-22.

49. Berrang-Ford, L., et al., Vulnerability of indigenous health to climate change: A case study of Uganda's Batwa Pygmies. Social Science & Medicine, 2012. 75(6): p. 1067-1077.

50. Berry, P., et al., Risk Perception, Health Communication, and Adaptation to the Health Impacts of Climate Change in Canada, in Climate Change Adaptation in Developed Nations: From Theory to Practice, J.D. Ford and L. BerrangFord, Editors. 2011. p. 205-219.

51. Beymer-Farris, B.A. and T.J. Bassett, The REDD menace: Resurgent protectionism in Tanzania's mangrove forests. Global Environmental Change-Human and Policy Dimensions, 2012. 22(2): p. 332-341.

52. Bi, P., et al., The Effects of Extreme Heat on Human Mortality and Morbidity in Australia: Implications for Public Health. Asia-Pacific Journal of Public Health, 2011. 23(2): p. 27S-36S.

53. Biazin, B., et al., Rainwater harvesting and management in rainfed agricultural systems in sub-Saharan Africa - A review. Physics and Chemistry of the Earth, 2012. 47-48: p. 139-151.

54. Binternagel, N.B., et al., Adaptation to climate change in Indonesia - livelihood strategies of rural households in the face of ENSO related droughts, in Tropical Rainforests and Agroforests under Global Change: Ecological and Socio-economic Valuations, T. Tscharntke, et al., Editors. 2010. p. 351-375.

55. Blanco, A.V.R., Local initiatives and adaptation to climate change. Disasters, 2006. 30(1): p. 140-147.

56. Blennow, K., et al., Climate change and the probability of wind damage in two Swedish forests. Forest Ecology and Management, 2010. 259(4): p. 818-830.

57. Blennow, K. and J. Persson, Climate change: Motivation for taking measure to adapt. Global Environmental Change-Human and Policy Dimensions, 2009. 19(1): p. 100-104.

58. Bloetscher, F., B. Heimlich, and D.E. Meeroff, Development of an adaptation toolbox to protect southeast Florida water supplies from climate change. Environmental Reviews, 2011. 19: p. 397-417.

59. Bloetscher, F., et al., Improving resilience against the effects of climate change. Journal American Water Works Association, 2010. 102(11): p. 36-46.

60. Blondel, J., The 'Design' of mediterranean landscapes: A millennial story of humans and ecological systems during the historic period. Human Ecology, 2006. 34(5): p. 713-729.

61. Bolte, A., et al., Climate change and forest management - accordances and differences between the German states regarding assessments for needs and strategies towards forest adaptation. Landbauforschung Volkenrode, 2009. 59(4): p. 269-278.

62. Boomiraj, K., et al., Assessing the vulnerability of Indian mustard to climate change. Agriculture Ecosystems & Environment, 2010. 138(3-4): p. 265-273.

63. Bornman, M., et al., Implications for health education and intervention strategies arising from children's caregivers concerns following successful malaria control. Transactions of the Royal Society of Tropical Medicine and Hygiene, 2012. 106(7): p. 408-414.

64. Bown, P. and P. Pearson, Calcareous plankton evolution and the Paleocene/Eocene thermal maximum event: New evidence from Tanzania. Marine Micropaleontology, 2009. 71(1-2): p. 60-70.

65. Braun, B. and T. Assheuer, Floods in megacity environments: vulnerability and coping strategies of slum dwellers in Dhaka/Bangladesh. Natural Hazards, 2011. 58(2): p. 771-787.

66. Briceno, S., Investing Today for a Safer Future: How the Hyogo Framework for Action can Contribute to Reducing Deaths During Earthquakes. Earthquake Engineering in Europe, ed. M. Garevski and A. Ansal. Vol. 17. 2010. 441-461.

67. Brody, S., H. Grover, and A. Vedlitz, Examining the willingness of Americans to alter behaviour to mitigate climate change. Climate Policy, 2012. 12(1): p. 1-22.

68. Brondizio, E.S. and E.F. Moran, Human dimensions of climate change: the vulnerability of small farmers in the Amazon. Philosophical Transactions of the Royal Society B-Biological Sciences, 2008. 363(1498): p. 1803-1809.

69. Brouwer, R., et al., Socioeconomic vulnerability and adaptation to environmental risk: A case study of climate change and flooding in Bangladesh. Risk Analysis, 2007. 27(2): p. 313-326.

70. Brown, C., et al., Hydroclimate risk to economic growth in sub-Saharan Africa. Climatic Change, 2011. 106(4): p. 621-647.

71. Brown, D., Making the linkages between climate change adaptation and spatial planning in Malawi. Environmental Science & Policy, 2011. 14(8): p. 940-949.

72. Brown, H.C.P., Climate change and Ontario forests: Prospects for building institutional adaptive capacity. Mitigation and Adaptation Strategies for Global Change, 2009. 14(6): p. 513-536.

73. Brubaker, M., et al., Climate change and health effects in Northwest Alaska. Global health action, 2011. 4.

74. Brubaker, M.Y., et al., Climate change health assessment: a novel approach for Alaska Native communities. International Journal of Circumpolar Health, 2011. 70(3): p. 266-273.

75. Bryan, E., et al., Adaptation to climate change in Ethiopia and South Africa: options and constraints. Environmental Science & Policy, 2009. 12(4): p. 413-426.

76. Bryant, L., et al., Climate change and family planning: least-developed countries define the agenda. Bulletin of the World Health Organization, 2009. 87(11): p. 852-857.

77. Buhk, C., A. Meyn, and A. Jentsch, The challenge of plant regeneration after fire in the Mediterranean Basin: scientific gaps in our knowledge on plant strategies and evolution of traits. Plant Ecology, 2007. 192(1): p. 1-19.

78. Burbidge, A.A., et al., Is Australia ready for assisted colonization? Policy changes required to facilitate translocations under climate change. Pacific Conservation Biology, 2011. 17(3, Sp. Iss. SI): p. 259-269.

79. Buys, L., E. Miller, and K. van Megen, Conceptualising climate change in rural Australia: community perceptions, attitudes and (in)actions. Regional Environmental Change, 2012. 12(1): p. 237-248.

80. Calgaro, E. and K. Lloyd, Sun, sea, sand and tsunami: examining disaster vulnerability in the tourism community of Khao Lak, Thailand. Singapore Journal of Tropical Geography, 2008. 29(3): p. 288-306.

81. Callaghan, T.V., et al., A new climate era in the sub-Arctic: Accelerating climate changes and multiple impacts. Geophysical Research Letters, 2010. 37.

82. Camargo, C., et al., Community involvement in management for maintaining coral reef resilience and biodiversity in southern Caribbean marine protected areas. Biodiversity and Conservation, 2009. 18(4): p. 935-956.

83. Campbell, S., Ecological specialisation and conservation of Australia's Large-footed Myotis: a review of trawling bat behaviour. Biology and Conservation of Australasian Bats, ed. B. Law, et al. 2011. 72-85.

84. Carey, M., A. French, and E. O'Brien, Unintended effects of technology on climate change adaptation: an historical analysis of water conflicts below Andean Glaciers. Journal of Historical Geography, 2012. 38(2): p. 181-191.

85. Carlos Linares, J., A. Delgado-Huertas, and J. Antonio Carreira, Climatic trends and different drought adaptive capacity and vulnerability in a mixed Abies pinsapo-Pinus halepensis forest. Climatic Change, 2011. 105(1-2): p. 67-90.

86. Carnie, T.-L., et al., In their own words: Young people's mental health in drought-affected rural and remote NSW. Australian Journal of Rural Health, 2011. 19(5): p. 244-248.

87. Casale, M., et al., Understanding vulnerability in southern Africa: comparative findings using a multiple-stressor approach in South Africa and Malawi. Regional Environmental Change, 2010. 10(2): p. 157-168.

88. Cavatassi, R., L. Lipper, and U. Narloch, Modern variety adoption and risk management in drought prone areas: insights from the sorghum farmers of eastern Ethiopia. Agricultural Economics, 2011. 42(3): p. 279-292.

89. Ceccato, L., V. Giannini, and C. Giupponi, Participatory assessment of adaptation strategies to flood risk in the Upper Brahmaputra and Danube river basins. Environmental Science & Policy, 2011. 14(8): p. 1163-1174.

90. Chaliha, S., et al., Climate variability and farmer's vulnerability in a flood-prone district of Assam. International Journal of Climate Change Strategies and Management, 2012. 4(2): p. 179-200.

91. Chan, E.Y.Y., et al., Help-Seeking Behavior during Elevated Temperature in Chinese Population. Journal of Urban Health-Bulletin of the New York Academy of Medicine, 2011. 88(4): p. 637-650.

92. Chatterjee, M., Slum dwellers response to flooding events in the megacities of India. Mitigation and Adaptation Strategies for Global Change, 2010. 15(4): p. 337-353.

93. Chavunduka, C. and D.W. Bromley, Climate, carbon, civil war and flexible boundaries: Sudan's contested landscape. Land Use Policy, 2011. 28(4): p. 907-916.

94. Chazdon, R.L., Beyond deforestation: Restoring forests and ecosystem services on degraded lands. Science, 2008. 320(5882): p. 1458-1460.

95. Chen, L., T. Zuo, and R.G. Rasaily, Farmer's Adaptation to Climate Risk in the Context of China-A research on Jianghan Plain of Yangtze River Basin, in International Conference on Agricultural Risk and Food Security 2010, S. Xu, et al., Editors. 2010. p. 116-125.

96. Chen, N. and P. Graham, Climate Change as a Survival Strategy: Soft Infrastructure for Urban Resilience and Adaptive Capacity in Australia's Coastal Zones. Resilient Cities: Cities and Adaptation to Climate Change - Proceedings of the Global Forum 2010, ed. K. OttoZimmermann. Vol. 1. 2011. 379-388.

97. Cheng, K., et al., Carbon footprint of China's crop production-An estimation using agro-statistics data over 1993-2007. Agriculture Ecosystems & Environment, 2011. 142(3-4): p. 231-237.

98. Cheong, S.-M., Policy solutions in the U.S. Climatic Change, 2011. 106(1): p. 57-70.

99. Cheong, S.-M., The role of government in disaster management: the case of the Hebei Spirit oil spill compensation. Environment and Planning C-Government and Policy, 2011. 29(6): p. 1073-1086.

100. Chin, A., et al., An integrated risk assessment for climate change: analysing the vulnerability of sharks and rays on Australia's Great Barrier Reef. Global Change Biology, 2010. 16(7): p. 1936-1953.

101. Chmura, D.J., et al., Forest responses to climate change in the northwestern United States: Ecophysiological foundations for adaptive management. Forest Ecology and Management, 2011. 261(7): p. 1121-1142.

102. Chow, W.T.L., W.-C. Chuang, and P. Gober, Vulnerability to Extreme Heat in Metropolitan Phoenix: Spatial, Temporal, and Demographic Dimensions. Professional Geographer, 2012. 64(2): p. 286-302.

103. Ciscar, J.-C., et al., Physical and economic consequences of climate change in Europe. Proceedings of the National Academy of Sciences of the United States of America, 2011. 108(7): p. 2678-2683.

104. Clark, M.S. and L.S. Peck, HSP70 heat shock proteins and environmental stress in Antarctic marine organisms: A mini-review. Marine Genomics, 2009. 2(1): p. 11-18.

105. Cloern, J.E., et al., Projected evolution of California's San Francisco Bay-Delta-river system in a century of climate change. PloS one, 2011. 6(9): p. e24465-e24465.

106. Codjoe, S.N.A., L.K. Atidoh, and V. Burkett, Gender and occupational perspectives on adaptation to climate extremes in the Afram Plains of Ghana. Climatic Change, 2012. 110(1-2): p. 431-454.

107. Codjoe, S.N.A. and G. Owusu, Climate change/variability and food systems: evidence from the Afram Plains, Ghana. Regional Environmental Change, 2011. 11(4): p. 753-765.

108. Coffee, J.E., et al., Preparing for a changing climate: The Chicago climate action plan's adaptation strategy. Journal of Great Lakes Research, 2010. 36: p. 115-117.

109. Collings, P., Economic Strategies, Community, and Food Networks in Ulukhaktok, Northwest Territories, Canada. Arctic, 2011. 64(2): p. 207-219.

110. Colloff, M.J. and D.S. Baldwin, Resilience of floodplain ecosystems in a semi-arid environment. Rangeland Journal, 2010. 32(3): p. 305-314.

111. Cooper, P.J.M., et al., Coping better with current climatic variability in the rain-fed farming systems of sub-Saharan Africa: An essential first step in adapting to future climate change? Agriculture Ecosystems & Environment, 2008. 126(1-2): p. 24-35.

112. Cousins, B., et al., A synthesis of sociological and biological perspectives on sustainable land use in Namaqualand. Journal of Arid Environments, 2007. 70(4): p. 834-846.

113. Crispo, E., et al., Broken barriers: Human-induced changes to gene flow and introgression in animals. Bioessays, 2011. 33(7): p. 508-518.

114. Cunsolo Willox, A., et al., "From this place and of this place:" climate change, sense of place, and health in Nunatsiavut, Canada. Social science & medicine (1982), 2012. 75(3): p. 538-47.

115. D'Agostino, A.L. and B.K. Sovacool, Sewing climate-resilient seeds: implementing climate change adaptation best practices in rural Cambodia. Mitigation and Adaptation Strategies for Global Change, 2011. 16(6): p. 699-720.

116. Dantas-Torres, F., Biology and ecology of the brown dog tick, Rhipicephalus sanguineus. Parasites & Vectors, 2010. 3.

117. Dasgupta, A. and A. Baschieri, VULNERABILITY TO CLIMATE CHANGE IN RURAL GHANA: MAINSTREAMING CLIMATE CHANGE IN POVERTY-REDUCTION STRATEGIES. Journal of International Development, 2010. 22(6): p. 803-820.

118. de los Rios C, J.C. and J. Almeida, PERCEPTIONS AND ADAPTATION TECHNIQUES TO SOCIO-ENVIRONMENTAL RISKS IN PARAMO OF SONSON COLOMBIA. Cuadernos De Desarrollo Rural, 2010. 7(65): p. 107-124.

119. Derbile, E.K. and R.A. Kasei, Vulnerability of crop production to heavy precipitation in north-eastern Ghana. International Journal of Climate Change Strategies and Management, 2012. 4(1): p. 36-53.

120. Deressa, T.T., R.M. Hassan, and C. Ringler, Perception of and adaptation to climate change by farmers in the Nile basin of Ethiopia. Journal of Agricultural Science, 2011. 149: p. 23-31.

121. Diaz, J., C. Linares, and A. Tobias, Impact of extreme temperatures on daily mortality in Madrid (Spain) among the 45-64 age-group. International Journal of Biometeorology, 2006. 50(6): p. 342-348.

122. Djoudi, H. and M. Brockhaus, Is adaptation to climate change gender neutral? Lessons from communities dependent on livestock and forests in northern Mali. International Forestry Review, 2011. 13(2): p. 123-135.

123. Dodman, D., D. Mitlin, and J.R. Co, Victims to victors, disasters to opportunities Community-driven responses to climate change in the Philippines. International Development Planning Review, 2010. 32(1): p. 1-26.

124. Dodson, J., T. Li, and N. Sipe, Urban Structure and Socioeconomic Barriers to Consumer Adoption of Energy-Efficient Automobile Technology in a Dispersed City Case Study of Brisbane, Australia. Transportation Research Record, 2010(2157): p. 111-118.

125. Donner, S.D., Coping with Commitment: Projected Thermal Stress on Coral Reefs under Different Future Scenarios. PloS one, 2009. 4(6).

126. Douglas, E.M., et al., Coastal flooding, climate change and environmental justice: identifying obstacles and incentives for adaptation in two metropolitan Boston Massachusetts communities. Mitigation and Adaptation Strategies for Global Change, 2012. 17(5): p. 537-562.

127. Douglas, I., Climate change, flooding and food security in south Asia. Food Security, 2009. 1(2): p. 127-136.

128. Douglas, I., et al., Unjust waters: climate change, flooding and the urban poor in Africa. Environment and Urbanization, 2008. 20(1): p. 187-205.

129. Drimie, S. and S. Gillespie, Adaptation to climate change in Southern Africa: factoring in AIDS. Environmental Science & Policy, 2010. 13(8): p. 778-784.

130. Dugmore, A.J., C. Keller, and T.H. McGovern, Norse Greenland settlement: Reflections on climate change, trade, and the contrasting fates of human settlements in the North Atlantic islands. Arctic Anthropology, 2007. 44(1): p. 12-36.

131. Dumaru, P., Community-based adaptation: enhancing community adaptive capacity in Druadrua Island, Fiji. Wiley Interdisciplinary Reviews-Climate Change, 2010. 1(5): p. 751-763.

132. Duncan, K., Global climate change, air pollution, and women's health, in Management of Natural Resources, Sustainable Development and Ecological Hazards, C.A. Brebbia, M.E. Conti, and E. Tiezzi, Editors. 2007. p. 633-643.

133. Eakin, H. and L.A. Bojorquez-Tapia, Insights into the composition of household vulnerability from multicriteria decision analysis. Global Environmental Change-Human and Policy Dimensions, 2008. 18(1): p. 112-127.

134. Eakin, H., A. Winkels, and J. Sendzimir, Nested vulnerability: exploring cross-scale linkages and vulnerability teleconnections in Mexican and Vietnamese coffee systems. Environmental Science & Policy, 2009. 12(4): p. 398-412.

135. Eakin, H.C. and M.B. Wehbe, Linking local vulnerability to system sustainability in a resilience framework: two cases from Latin America. Climatic Change, 2009. 93(3-4): p. 355-377.

136. Easdale, M.H. and H. Rosso, Dealing with drought: social implications of different smallholder survival strategies in semi-arid rangelands of Northern Patagonia, Argentina. Rangeland Journal, 2010. 32(2): p. 247-255.

137. Ebi, K.L., et al., US Funding Is Insufficient to Address the Human Health Impacts of and Public Health Responses to Climate Variability and Change. Environmental Health Perspectives, 2009. 117(6): p. 857-862.

138. Ebi, K.L. and J.C. Semenza, Community-Based Adaptation to the Health Impacts of Climate Change. American Journal of Preventive Medicine, 2008. 35(5): p. 501-507.

139. Egeru, A., Role of Indigenous Knowledge in Climate Change Adaptation: A case study of the Teso Sub-Region, Eastern Uganda. Indian Journal of Traditional Knowledge, 2012. 11(2): p. 217-224.

140. Einarsson, N., From good to eat to good to watch: whale watching, adaptation and change in Icelandic fishing communities. Polar Research, 2009. 28(1): p. 129-138.

141. Engle, N.L. and M.C. Lemos, Unpacking governance: Building adaptive capacity to climate change of river basins in Brazil. Global Environmental Change-Human and Policy Dimensions, 2010. 20(1): p. 4-13.

142. English, P.B., et al., Environmental Health Indicators of Climate Change for the United States: Findings from the State Environmental Health Indicator Collaborative. Environmental Health Perspectives, 2009. 117(11): p. 1673-1681.

143. Eriksen, S. and E. Selboe, The social organisation of adaptation to climate variability and global change: The case of a mountain farming community in Norway. Applied Geography, 2012. 33(1): p. 159-167.

144. Eriksen, S. and J.A. Silva, The vulnerability context of a savanna area in Mozambique: household drought coping strategies and responses to economic change. Environmental Science & Policy, 2009. 12(1): p. 33-52.

145. Eugenio Barrios, J., J. Alfredo Rodriguez-Pineda, and M. de la Maza Benignos, Integrated river basin management in the Conchos River basin, Mexico: A case study of freshwater climate change adaptation. Climate and Development, 2009. 1(3): p. 249-260.

146. Fang, Y., Adaptation Management of Mountain Tourism Service: The Case of the Source Regions of the Yangtze and Yellow River. Journal of Mountain Science, 2009. 6(3): p. 299-310.

147. Fatti, C.E. and C. Vogel, Is science enough? Examining ways of understanding, coping with and adapting to storm risks in Johannesburg. Water Sa, 2011. 37(1): p. 57-65.

148. Fazey, I., et al., A three-tiered approach to participatory vulnerability assessment in the Solomon Islands. Global Environmental Change-Human and Policy Dimensions, 2010. 20(4): p. 713-728.

149. Fazey, I., et al., Maladaptive trajectories of change in Makira, Solomon Islands. Global Environmental Change-Human and Policy Dimensions, 2011. 21(4): p. 1275-1289.

150. Fischer, T., et al., Probability Distribution of Precipitation Extremes for Weather Index-Based Insurance in the Zhujiang River Basin, South China. Journal of Hydrometeorology, 2012. 13(3): p. 1023-1037.

151. Fisher, M., M. Chaudhury, and B. McCusker, Do Forests Help Rural Households Adapt to Climate Variability? Evidence from Southern Malawi. World Development, 2010. 38(9): p. 1241-1250.

152. Flugman, E., P. Mozumder, and T. Randhir, Facilitating adaptation to global climate change: perspectives from experts and decision makers serving the Florida Keys. Climatic Change, 2012. 112(3-4): p. 1015-1035.

153. Fontaine, J.J., et al., Spatial and temporal variation in climate change: a bird's eye view. Climatic Change, 2009. 97(1-2): p. 305-311.

154. Ford, J., et al., Reducing vulnerability to climate change in the Arctic: The case of Nunavut, Canada. Arctic, 2007. 60(2): p. 150-166.

155. Ford, J.D., Dangerous climate change and the importance of adaptation for the Arctic's Inuit population. Environmental Research Letters, 2009. 4(2).

156. Ford, J.D., Vulnerability of Inuit food systems to food insecurity as a consequence of climate change: a case study from Igloolik, Nunavut. Regional Environmental Change, 2009. 9(2): p. 83-100.

157. Ford, J.D., Indigenous Health and Climate Change. American Journal of Public Health, 2012. 102(7): p. 1260-1266.

158. Ford, J.D., et al., Sea ice, climate change, and community vulnerability in northern Foxe Basin, Canada. Climate Research, 2009. 38(2): p. 137-154.

159. Ford, J.D. and T. Pearce, Climate change vulnerability and adaptation research focusing on the Inuit subsistence sector in Canada: Directions for future research. Canadian Geographer-Geographe Canadien, 2012. 56(2): p. 275-287.

160. Ford, J.D., et al., Climate change policy responses for Canada's Inuit population: The importance of and opportunities for adaptation. Global Environmental Change-Human and Policy Dimensions, 2010. 20(1): p. 177-191.

161. Ford, J.D., B. Smit, and J. Wandel, Vulnerability to climate change in the Arctic: A case study from Arctic Bay, Canada. Global Environmental Change-Human and Policy Dimensions, 2006. 16(2): p. 145-160.

162. Ford, J.D., et al., Vulnerability to climate change in Igloolik, Nunavut: what we can learn from the past and present. Polar Record, 2006. 42(221): p. 127-138.

163. Forsberg, B., et al., An expert assessment on climate change and health - with a European focus on lungs and allergies. Environmental Health, 2012. 11.

164. Fraser, E.D.G., Food system vulnerability: Using past famines to help understand how food systems may adapt to climate change. Ecological Complexity, 2006. 3(4): p. 328-335.

165. Fraser, E.D.G., Travelling in antique lands: using past famines to develop an adaptability/resilience framework to identify food systems vulnerable to climate change. Climatic Change, 2007. 83(4): p. 495-514.

166. Fraser, E.D.G., et al., Assessing Vulnerability to Climate Change in Dryland Livelihood Systems: Conceptual Challenges and Interdisciplinary Solutions. Ecology and Society, 2011. 16(3).

167. Frazier, T.G., N. Wood, and B. Yarnal, Stakeholder perspectives on land-use strategies for adapting to climate-change-enhanced coastal hazards: Sarasota, Florida. Applied Geography, 2010. 30(4): p. 506-517.

168. Frazier, T.G., et al., Influence of potential sea level rise on societal vulnerability to hurricane storm-surge hazards, Sarasota County, Florida. Applied Geography, 2010. 30(4): p. 490-505.

169. Fritze, H., I.T. Stewart, and E. Pebesma, Shifts in Western North American Snowmelt Runoff Regimes for the Recent Warm Decades. Journal of Hydrometeorology, 2011. 12(5): p. 989-1006.

170. Fu, Y., et al., Climate change adaptation among tibetan pastoralists: challenges in enhancing local adaptation through policy support. Environmental Management, 2012. 50(4): p. 607-21.

171. Furgal, C. and J. Seguin, Climate change, health, and vulnerability in Canadian northern Aboriginal communities. Environmental Health Perspectives, 2006. 114(12): p. 1964-1970.

172. Furlow, J., et al., Building resilience to climate change through development assistance: USAID's climate adaptation program. Climatic Change, 2011. 108(3): p. 411-421.

173. Furman, C., et al., Beyond the "fit": introducing climate forecasts among organic farmers in Georgia (United States). Climatic Change, 2011. 109(3-4): p. 791-799.

174. Galiano, L., et al., Determinants of drought effects on crown condition and their relationship with depletion of carbon reserves in a Mediterranean holm oak forest. Tree Physiology, 2012. 32(4): p. 478-489.

175. Gardali, T., et al., A climate change vulnerability assessment of California's at-risk birds. PloS one, 2012. 7(3): p. e29507-e29507.

176. Garrelts, H. and H. Lange, Path Dependencies and Path Change in Complex Fields of Action: Climate Adaptation Policies in Germany in the Realm of Flood Risk Management. Ambio, 2011. 40(2): p. 200-209.

177. Garschagen, M., F.G. Renaud, and J. Birkmann, Dynamic Resilience of Peri-Urban Agriculturalists in the Mekong Delta Under Pressures of Socio-Economic Transformation and Climate Change, in Environmental Change and Agricultural Sustainability in the Mekong Delta, M.A. Stewart and P.A. Coclanis, Editors. 2011. p. 141-163.

178. Gartner, K., M. Englisch, and E. Leitgeb, Effects of Climate Change on the Vulnerability of Norway Spruce Stands - Soil Hydrological Constraints for Forest Management in Austria's Lowlands, in Forest Management and the Water Cycle: An Ecosystem-Based Approach, M. Bredemeier, et al., Editors. 2011. p. 127-140.

179. Gentle, P. and T.N. Maraseni, Climate change, poverty and livelihoods: adaptation practices by rural mountain communities in Nepal. Environmental Science & Policy, 2012. 21: p. 24-34.

180. Gero, A., K. Meheux, and D. Dominey-Howes, Integrating disaster risk reduction and climate change adaptation in the Pacific. Climate and Development, 2011. 3(4): p. 310-327.

181. Gero, A., K. Meheux, and D. Dominey-Howes, Integrating community based disaster risk reduction and climate change adaptation: examples from the Pacific. Natural Hazards and Earth System Sciences, 2011. 11(1): p. 101-113.

182. Ghimire, Y.N., G.P. Shivakoti, and S.R. Perret, Household-level vulnerability to drought in hill agriculture of Nepal: implications for adaptation planning. International Journal of Sustainable Development and World Ecology, 2010. 17(3): p. 225-230.

183. Gilbert, G. and R. McLeman, Household access to capital and its effects on drought adaptation and migration: a case study of rural Alberta in the 1930s. Population and Environment, 2010. 32(1): p. 3-26.

184. Glavovic, B.C., W.S.A. Saunders, and J.S. Becker, Land-use planning for natural hazards in New Zealand: the setting, barriers, 'burning issues' and priority actions. Natural Hazards, 2010. 54(3): p. 679-706.

185. Godden, L. and A. Kung, Water Law and Planning Frameworks Under Climate Change Variability: Systemic and Adaptive Management of Flood Risk. Water Resources Management, 2011. 25(15): p. 4051-4068.

186. Gooch, M., et al., Community-Derived Indicator Domains for Social Resilience to Water Quality Decline in a Great Barrier Reef Catchment, Australia. Society & Natural Resources, 2012. 25(5): p. 421-439.

187. Gray, C. and V. Mueller, Drought and Population Mobility in Rural Ethiopia. World Development, 2012. 40(1): p. 134-145.

188. Gray, C.L. and V. Mueller, Natural disasters and population mobility in Bangladesh. Proceedings of the National Academy of Sciences of the United States of America, 2012. 109(16): p. 6000-6005.

189. Green, D., et al., An assessment of climate change impacts and adaptation for the Torres Strait Islands, Australia. Climatic Change, 2010. 102(3-4): p. 405-433.

190. Grewe, H.A., et al., Community-Based Prevention of Climate-Associated Health Risks. Gesundheitswesen, 2010. 72(8-9): p. 466-471.

191. Grimm, K., Katrina, Wilma and me: Learning to live with climate surprises? Geoscience Canada, 2006. 33(2): p. 76-80.

192. Guleria, S. and J.K.P. Edward, Coastal community resilience: analysis of resilient elements in 3 districts of Tamil Nadu State, India. Journal of Coastal Conservation, 2012. 16(1): p. 101-110.

193. Gyenge, J., et al., Stand density and drought interaction on water relations of Nothofagus antarctica: contribution of forest management to climate change adaptability. Trees-Structure and Function, 2011. 25(6): p. 1111-1120.

194. Hahn, M.B., A.M. Riederer, and S.O. Foster, The Livelihood Vulnerability Index: A pragmatic approach to assessing risks from climate variability and change-A case study in Mozambique. Global Environmental Change-Human and Policy Dimensions, 2009. 19(1): p. 74-88.

195. Hamisi, H.I., et al., Crisis in the wetlands: Combined stresses in a changing climate - Experience from Tanzania. Climate and Development, 2012. 4(1): p. 5-15.

196. Hammi, S., et al., Can traditional forest management buffer forest depletion? Dynamics of Moroccan High Atlas Mountain forests using remote sensing and vegetation analysis. Forest Ecology and Management, 2010. 260(10): p. 1861-1872.

197. Han, Y. and X.-y. Hou, Perceptions and adaptation strategies of herders in desert steppe of Inner Mongolia to climate change. Yingyong Shengtai Xuebao, 2011. 22(4): p. 913-922.

198. Haque, M.A., et al., Households' perception of climate change and human health risks: A community perspective. Environmental Health, 2012. 11.

199. Hardoy, J. and P.R. Lankao, Latin American cities and climate change: challenges and options to mitigation and adaptation responses. Current Opinion in Environmental Sustainability, 2011. 3(3): p. 158-163.

200. Hardoy, J. and G. Pandiella, Urban poverty and vulnerability to climate change in Latin America. Environment and Urbanization, 2009. 21(1): p. 203-224.

201. Hart, C.R., H.L. Berry, and A.M. Tonna, Improving the mental health of rural New South Wales communities facing drought and other adversities. Australian Journal of Rural Health, 2011. 19(5): p. 231-238.

202. Harvey, N. and C.D. Woodroffe, Australian approaches to coastal vulnerability assessment. Sustainability Science, 2008. 3(1): p. 67-87.

203. Hassan, R.M., Implications of Climate Change for Agricultural Sector Performance in Africa: Policy Challenges and Research Agenda(dagger). Journal of African Economies, 2010. 19: p. ii77-ii105.

204. Hay, J. and N. Mimura, Supporting climate change vulnerability and adaptation assessments in the Asia-Pacific region: an example of sustainability science. Sustainability Science, 2006. 1(1): p. 23-35.

205. Hayasaka, D., et al., Ecological impacts of the 2004 Indian Ocean tsunami on coastal sand-dune species on Phuket Island, Thailand. Biodiversity and Conservation, 2012. 21(8): p. 1971-1985.

206. Hayden, M.H., H. Brenkert-Smith, and O.V. Wilhelmi, Differential Adaptive Capacity to Extreme Heat: A Phoenix, Arizona, Case Study. Weather Climate and Society, 2011. 3(4): p. 269-280.

207. Hayman, P., et al., Climate change through the farming systems lens: challenges and opportunities for farming in Australia. Crop & Pasture Science, 2012. 63(3): p. 203-214.

208. Head, L., et al., A Fine-Grained Study of the Experience of Drought, Risk and Climate Change Among Australian Wheat Farming Households. Annals of the Association of American Geographers, 2011. 101(5): p. 1089-1108.

209. Healey, G.K., et al., Community Perspectives on the Impact of Climate Change on Health in Nunavut, Canada. Arctic, 2011. 64(1): p. 89-97.

210. Heberger, M., et al., Potential impacts of increased coastal flooding in California due to sea-level rise. Climatic Change, 2011. 109: p. 229-249.

211. Hedger, M., M. Greeley, and J. Leavy, Evaluating Climate Change: Pro-Poor Perspectives. Ids Bulletin-Institute of Development Studies, 2008. 39(4): p. 75-+.

212. Hendrickson, J., et al., Interactions in integrated US agricultural systems: The past, present and future. Renewable Agriculture and Food Systems, 2008. 23(4): p. 314-324.

213. Hertzler, G., Adapting to climate change and managing climate risks by using real options. Australian Journal of Agricultural Research, 2007. 58(10): p. 985-992.

214. Hess, J.J., J.N. Malilay, and A.J. Parkinson, Climate Change The Importance of Place. American Journal of Preventive Medicine, 2008. 35(5): p. 468-478.

215. Hill, M., A. Wallner, and J. Furtado, Reducing vulnerability to climate change in the Swiss Alps: a study of adaptive planning. Climate Policy, 2010. 10(1): p. 70-86.

216. Hobson, K. and S. Niemeyer, Public responses to climate change: The role of deliberation in building capacity for adaptive action. Global Environmental Change-Human and Policy Dimensions, 2011. 21(3): p. 957-971.

217. Hoegh-Guldberg, O., The adaptation of coral reefs to climate change: Is the Red Queen being outpaced? Scientia Marina, 2012. 76(2): p. 403-408.

218. Hogan, A., A. Bode, and H. Berry, Farmer Health and Adaptive Capacity in the Face of Climate Change and Variability. Part 2: Contexts, Personal Attributes and Behaviors. International Journal of Environmental Research and Public Health, 2011. 8(10): p. 4055-4068.

219. Horton, G., L. Hanna, and B. Kelly, Drought, drying and climate change: Emerging health issues for ageing Australians in rural areas. Australasian Journal on Ageing, 2010. 29(1): p. 2-7.

220. Howe, P.D., Hurricane preparedness as anticipatory adaptation: A case study of community businesses. Global Environmental Change-Human and Policy Dimensions, 2011. 21(2): p. 711-720.

221. Huang, C., et al., The impact of temperature on years of life lost in Brisbane, Australia. Nature Climate Change, 2012. 2(4): p. 265-270.

222. Huigen, M.G.A., et al., Simulation of climate change effects on the agricultural sector in the Danubia catchment in South Germany with a generic modeling framework. 18th World Imacs Congress and Modsim09 International Congress on Modelling and Simulation: Interfacing Modelling and Simulation with Mathematical and Computational Sciences, ed. R.S. Anderssen, R.D. Braddock, and L.T.H. Newham. 2009. 894-894.

223. Huntington, H.P., E. Goodstein, and E. Euskirchen, Towards a Tipping Point in Responding to Change: Rising Costs, Fewer Options for Arctic and Global Societies. Ambio, 2012. 41(1): p. 66-74.

224. Ireland, P. and F. Thomalla, The role of collective action in enhancing communities' adaptive capacity to environmental risk: an exploration of two case studies from Asia. PLoS currents, 2011. 3: p. RRN1279-RRN1279.

225. Iwasaki, S., B.H.N. Razafindrabe, and R. Shaw, Fishery livelihoods and adaptation to climate change: a case study of Chilika lagoon, India. Mitigation and Adaptation Strategies for Global Change, 2009. 14(4): p. 339-355.

226. Jabeen, H., C. Johnson, and A. Allen, Built-in resilience: learning from grassroots coping strategies for climate variability. Environment and Urbanization, 2010. 22(2): p. 415-431.

227. Jacobsen, S.E., C.R. Jensen, and F. Liu, Improving crop production in the arid Mediterranean climate. Field Crops Research, 2012. 128: p. 34-47.

228. Janes, C.R., Failed Development and Vulnerability to Climate Change in Central Asia: Implications for Food Security and Health. Asia-Pacific Journal of Public Health, 2010. 22: p. 236S-245S.

229. Janssen, M.A., Population Aggregation in Ancient Arid Environments. Ecology and Society, 2010. 15(2).

230. Joerin, J., et al., Action-oriented resilience assessment of communities in Chennai, India. Environmental Hazards-Human and Policy Dimensions, 2012. 11(3): p. 226-241.

231. Johnstone, W.M. and B.J. Lence, Assessing the value of mitigation strategies in reducing the impacts of rapid-onset, catastrophic floods. Journal of Flood Risk Management, 2009. 2(3): p. 209-221.

232. Jones, A.R., W. Gladstone, and N.J. Hacking, Australian sandy-beach ecosystems and climate change: ecology and management. Australian Zoologist, 2007. 34(2): p. 190-202.

233. Jones, M.T. and P.R. Sievert, Effects of Stochastic Flood Disturbance on Adult Wood Turtles, Glyptemys insculpta, in Massachusetts. Canadian Field-Naturalist, 2009. 123(4): p. 313-322.

234. Kabubo-Mariara, J., Climate change adaptation and livestock activity choices in Kenya: An economic analysis. Natural Resources Forum, 2008. 32(2): p. 131-141.

235. Kakota, T., et al., Gender vulnerability to climate variability and household food insecurity. Climate and Development, 2011. 3(4): p. 298-309.

236. Kalame, F.B., D. Kudejira, and J. Nkem, Assessing the process and options for implementing National Adaptation Programmes of Action (NAPA): a case study from Burkina Faso. Mitigation and Adaptation Strategies for Global Change, 2011. 16(5): p. 535-553.

237. Kampragou, E., et al., Towards the harmonization of water-related policies for managing drought risks across the EU. Environmental Science & Policy, 2011. 14(7): p. 815-824.

238. Kenny, G.P., et al., Heat stress in older individuals and patients with common chronic diseases. Canadian Medical Association Journal, 2010. 182(10): p. 1053-1060.

239. Keogh, D.U., et al., Resilience, vulnerability and adaptive capacity of an inland rural town prone to flooding: a climate change adaptation case study of Charleville, Queensland, Australia. Natural Hazards, 2011. 59(2): p. 699-723.

240. Keskitalo, E.C.H., et al., Adaptive capacity determinants in developed states: examples from the Nordic countries and Russia. Regional Environmental Change, 2011. 11(3): p. 579-592.

241. Keskitalo, E.C.H. and A.A. Kulyasova, The role of governance in community adaptation to climate change. Polar Research, 2009. 28(1): p. 60-70.

242. Kiltz, L., The Challenges of Developing a Homeland Security Discipline to Meet Future Threats to the Homeland. Journal of Homeland Security and Emergency Management, 2011. 8(2).

243. Kingsford, R.T. and J.E.M. Watson, Climate Change in Oceania - A synthesis of biodiversity impacts and adaptations. Pacific Conservation Biology, 2011. 17(3, Sp. Iss. SI): p. 270-284.

244. Kjellstrom, T. and H.J. Weaver, Climate change and health: impacts, vulnerability, adaptation and mitigation. New South Wales public health bulletin, 2009. 20(1-2): p. 5-9.

245. Klenk, N.L., et al., Climate change adaptation and sustainable forest management: A proposed reflexive research agenda. Forestry Chronicle, 2011. 87(3): p. 351-357.

246. Klint, L.M., et al., Climate change adaptation in the Pacific Island tourism sector: analysing the policy environment in Vanuatu. Current Issues in Tourism, 2012. 15(3): p. 247-274.

247. Knowlton, K., et al., The 2006 California Heat Wave: Impacts on Hospitalizations and Emergency Department Visits. Environmental Health Perspectives, 2009. 117(1): p. 61-67.

248. Koike, T. and T. Oki, Time for a Change in Japanese Water Resources Policy, Part 2: Towards a Planning and Management Framework for Adapting to Changes. International Journal of Water Resources Development, 2009. 25(4): p. 565-570.

249. Kreibich, H., Do perceptions of climate change influence precautionary measures? International Journal of Climate Change Strategies and Management, 2011. 3(2): p. 189-199.

250. Krishnamurthy, P.K., J.B. Fisher, and C. Johnson, Mainstreaming local perceptions of hurricane risk into policymaking: A case study of community GIS in Mexico. Global Environmental Change-Human and Policy Dimensions, 2011. 21(1): p. 143-153.

251. Kwiatkowski, R.E., Indigenous community based participatory research and health impact assessment: A Canadian example. Environmental Impact Assessment Review, 2011. 31(4): p. 445-450.

252. Lagos, P., Peru's approach to climate change in the Andean mountain region: Achieving multidisciplinary regional cooperation for integrated assessment of climate change. Mountain Research and Development, 2007. 27(1): p. 28-31.

253. Lahiri-Dutt, K., 'Like the drifting grains of Sand: Vulnerability, security and adjustment by communities in the charlands of the damodar river, India. South Asia-Journal of South Asian Studies, 2007. 30(2): p. 327-349.

254. Laidler, G.J., et al., Travelling and hunting in a changing Arctic: assessing Inuit vulnerability to sea ice change in Igloolik, Nunavut. Climatic Change, 2009. 94(3-4): p. 363-397.

255. Lal, P., J.R.R. Alavalapati, and E.D. Mercer, Socio-economic impacts of climate change on rural United States. Mitigation and Adaptation Strategies for Global Change, 2011. 16(7): p. 819-844.

256. Lantz, V., et al., Assessing market and non-market costs of freshwater flooding due to climate change in the community of Fredericton, Eastern Canada. Climatic Change, 2012. 110(1-2): p. 347-372.

257. Lata, S. and P. Nunn, Misperceptions of climate-change risk as barriers to climate-change adaptation: a case study from the Rewa Delta, Fiji. Climatic Change, 2012. 110(1-2): p. 169-186.

258. Lebel, L., T. Grothmann, and B. Siebenhuener, The role of social learning in adaptiveness: insights from water management. International Environmental Agreements-Politics Law and Economics, 2010. 10(4): p. 333-353.

259. Lebel, L., J.B. Manuta, and P. Garden, Institutional traps and vulnerability to changes in climate and flood regimes in Thailand. Regional Environmental Change, 2011. 11(1): p. 45-58.

260. Lemelin, H., et al., Climate change, wellbeing and resilience in the Weenusk First Nation at Peawanuck: the Moccasin Telegraph goes global. Rural and Remote Health, 2010. 10(2).

261. Leys, A.J. and J.K. Vanclay, Social learning: A knowledge and capacity building approach for adaptive co-management of contested landscapes. Land Use Policy, 2011. 28(3): p. 574-584.

262. Lindgren, E., A. Albihn, and Y. Andersson, Climate Change, Water-Related Health Impacts, and Adaptation: Highlights from the Swedish Government's Commission on Climate and Vulnerability, in Climate Change Adaptation in Developed Nations: From Theory to Practice, J.D. Ford and L. BerrangFord, Editors. 2011. p. 177-188.

263. Linnekamp, F., A. Koedam, and I.S.A. Baud, Household vulnerability to climate change: Examining perceptions of households of flood risks in Georgetown and Paramaribo. Habitat International, 2011. 35(3): p. 447-456.

264. Linnerooth-Bayer, J. and R. Mechler, Insurance for assisting adaptation to climate change in developing countries: a proposed strategy. Climate Policy, 2006. 6(6): p. 621-636.

265. Littell, J.S., et al., Climate and wildfire area burned in western U. S. ecoprovinces, 1916-2003. Ecological Applications, 2009. 19(4): p. 1003-1021.

266. Littell, J.S., et al., U.S. National Forests adapt to climate change through Science-Management partnerships. Climatic Change, 2012. 110(1-2): p. 269-296.

267. Liu, C., D. Golding, and G. Gong, Farmers' coping response to the low flows in the lower Yellow River: A case study of temporal dimensions of vulnerability. Global Environmental Change-Human and Policy Dimensions, 2008. 18(4): p. 543-553.

268. Liu, J., et al., A spatially explicit assessment of current and future hotspots of hunger in Sub-Saharan Africa in the context of global change. Global and Planetary Change, 2008. 64(3-4): p. 222-235.

269. Lloret, J. and V. Riera, Evolution of a Mediterranean Coastal Zone: Human Impacts on the Marine Environment of Cape Creus. Environmental Management, 2008. 42(6): p. 977-988.

270. Lloyd, D.L., et al., Action learning in partnership with Landcare and catchment management groups to support increased pasture sowings in southern inland Queensland. Animal Production Science, 2009. 49(9-10): p. 907-915.

271. Lopes, P.F.M., R.A.M. Silvano, and A. Begossi, Extractive and Sustainable Development Reserves in Brazil: resilient alternatives to fisheries? Journal of Environmental Planning and Management, 2011. 54(4): p. 421-443.

272. Lopez-Larrosa, S., The family system upon divorce: Risk and protective factors and intervention programmes. Cultura Y Educacion, 2009. 21(4): p. 391-402.

273. Lopez-Marrero, T., An integrative approach to study and promote natural hazards adaptive capacity: a case study of two flood-prone communities in Puerto Rico. Geographical Journal, 2010. 176: p. 150-163.

274. Lopez-Marrero, T. and B. Yarnal, Putting adaptive capacity into the context of people's lives: a case study of two flood-prone communities in Puerto Rico. Natural Hazards, 2010. 52(2): p. 277-297.

275. Loring, P.A., et al., Ways to Help and Ways to Hinder: Governance for Effective Adaptation to an Uncertain Climate. Arctic, 2011. 64(1): p. 73-88.

276. Loucks, C., et al., Sea level rise and tigers: predicted impacts to Bangladesh's Sundarbans mangroves. Climatic Change, 2010. 98(1-2): p. 291-298.

277. Lundmark, L., et al., Effects of climate change and extreme events on forest communities in the European North. Climatic Change, 2008. 87(1-2): p. 235-249.

278. Luxereau, A., P. Genthon, and J.-M.A. Karimou, Fluctuations in the size of Lake Chad: consequences on the livelihoods of the riverain peoples in eastern Niger. Regional Environmental Change, 2012. 12(3): p. 507-521.

279. Lwasa, S., Adapting urban areas in Africa to climate change: the case of Kampala. Current Opinion in Environmental Sustainability, 2010. 2(3): p. 166-171.

280. Lynch, A.H. and R.D. Brunner, Context and climate change: An integrated assessment for Barrow, Alaska. Climatic Change, 2007. 82(1-2): p. 93-111.

281. Manderson, L. and T. Victoria, Social and public health effects of climate change in the '40 South'. Wiley Interdisciplinary Reviews-Climate Change, 2011. 2(6): p. 902-918.

282. Marchand, F.L., et al., Are heat and cold resistance of arctic species affected by successive extreme temperature events? New Phytologist, 2006. 170(2): p. 291-300.

283. Marino, E., The long history of environmental migration: Assessing vulnerability construction and obstacles to successful relocation in Shishmaref, Alaska. Global Environmental Change-Human and Policy Dimensions, 2012. 22(2): p. 374-381.

284. Markoff, M.S. and A.C. Cullen, Impact of climate change on Pacific Northwest hydropower. Climatic Change, 2008. 87(3-4): p. 451-469.

285. Marquez-Caraveo, M.E., et al., Psychometric data of the EMBU-C "My memories of upbringing" as indicator of child rearing perception in an adolescent sample in Mexico City. Salud Mental, 2007. 30(2): p. 58-66.

286. Martin, D., et al., Drinking water and potential threats to human health in Nunavik: Adaptation strategies under climate change conditions. Arctic, 2007. 60(2): p. 195-202.

287. Martin-Benito, D., et al., Response of climate-growth relationships and water use efficiency to thinning in a Pinus nigra afforestation. Forest Ecology and Management, 2010. 259(5): p. 967-975.

288. Mathew, S., S. Trueck, and A. Henderson-Sellers, Kochi, India case study of climate adaptation to floods: Ranking local government investment options. Global Environmental Change-Human and Policy Dimensions, 2012. 22(1): p. 308-319.

289. Mathot, K.J., et al., Adaptive strategies for managing uncertainty may explain personality-related differences in behavioural plasticity. Oikos, 2012. 121(7): p. 1009-1020.

290. Matson, P.G., T.R. Martz, and G.E. Hofmann, High-frequency observations of pH under Antarctic sea ice in the southern Ross Sea. Antarctic Science, 2011. 23(6): p. 607-613.

291. Maynard, N.G. and G.A. Conway, A view from above: use of satellite imagery to enhance our understanding of potential impacts of climate change on human health in the Arctic. Alaska medicine, 2007. 49(2 Suppl): p. 38-43.

292. Mbow, C., et al., The history of environmental change and adaptation in eastern Saloum-Senegal-Driving forces and perceptions. Global and Planetary Change, 2008. 64(3-4): p. 210-221.

293. McClanahan, T.R., et al., Identifying Reefs of Hope and Hopeful Actions: Contextualizing Environmental, Ecological, and Social Parameters to Respond Effectively to Climate Change. Conservation Biology, 2009. 23(3): p. 662-671.

294. McDaniels, T., et al., Vulnerability of Fraser River sockeye salmon to climate change: A life cycle perspective using expert judgments. Journal of Environmental Management, 2010. 91(12): p. 2771-2780.

295. McDowell, J.Z. and J.J. Hess, Accessing adaptation: Multiple stressors on livelihoods in the Bolivian highlands under a changing climate. Global Environmental Change-Human and Policy Dimensions, 2012. 22(2): p. 342-352.

296. McEvoy, J. and M. Wilder, Discourse and desalination: Potential impacts of proposed climate change adaptation interventions in the Arizona-Sonora border region. Global Environmental Change-Human and Policy Dimensions, 2012. 22(2): p. 353-363.

297. McGinnity, P., et al., Impact of naturally spawning captive-bred Atlantic salmon on wild populations: depressed recruitment and increased risk of climate-mediated extinction. Proceedings of the Royal Society B-Biological Sciences, 2009. 276(1673): p. 3601-3610.

298. McLachlan, S.M. and M. Yestrau, From the ground up: holistic management and grassroots rural adaptation to bovine spongiform encephalopathy across western Canada. Mitigation and Adaptation Strategies for Global Change, 2009. 14(4): p. 299-316.

299. McLeman, R. and B. Smit, Vulnerability to climate change hazards and risks: crop and flood insurance. Canadian Geographer-Geographe Canadien, 2006. 50(2): p. 217-226.

300. McLeman, R.A., et al., Opportunities and Barriers for Adaptation and Local Adaptation Planning in Canadian Rural and Resource-Based Communities, in Climate Change Adaptation in Developed Nations: From Theory to Practice, J.D. Ford and L. BerrangFord, Editors. 2011. p. 449-459.

301. McMichael, C., J. Barnett, and A.J. McMichael, An Ill Wind? Climate Change, Migration, and Health. Environmental Health Perspectives, 2012. 120(5): p. 646-654.

302. McNeeley, S.M., Examining barriers and opportunities for sustainable adaptation to climate change in Interior Alaska. Climatic Change, 2012. 111(3-4): p. 835-857.

303. McNeeley, S.M. and M.D. Shulski, Anatomy of a closing window: Vulnerability to changing seasonality in Interior Alaska. Global Environmental Change-Human and Policy Dimensions, 2011. 21(2): p. 464-473.

304. McSweeney, K. and O.T. Coomes, Climate-related disaster opens a window of opportunity for rural poor in northeastern Honduras. Proceedings of the National Academy of Sciences of the United States of America, 2011. 108(13): p. 5203-5208.

305. Mdluli, T.N. and C.H. Vogel, Challenges to achieving a successful transition to a low carbon economy in South Africa: examples from poor urban communities. Mitigation and Adaptation Strategies for Global Change, 2010. 15(3): p. 205-222.

306. Meenawat, H. and B.K. Sovacool, Improving adaptive capacity and resilience in Bhutan. Mitigation and Adaptation Strategies for Global Change, 2011. 16(5): p. 515-533.

307. Mendelsohn, R., A. Dinar, and L. Williams, The distributional impact of climate change on rich and poor countries. Environment and Development Economics, 2006. 11: p. 159-178.

308. Menon, N., Rainfall Uncertainty and Occupational Choice in Agricultural Households of Rural Nepal. Journal of Development Studies, 2009. 45(6): p. 864-888.

309. Mertz, O., et al., Sustainable land use in Tikopia: Food production and consumption in an isolated agricultural system. Singapore Journal of Tropical Geography, 2010. 31(1): p. 10-26.

310. Mertz, O., et al., Climate Variability and Environmental Stress in the Sudan-Sahel Zone of West Africa. Ambio, 2012. 41(4): p. 380-392.

311. Mertz, O., et al., Climate Factors Play a Limited Role for Past Adaptation Strategies in West Africa. Ecology and Society, 2010. 15(4).

312. Mertz, O., et al., Farmers' Perceptions of Climate Change and Agricultural Adaptation Strategies in Rural Sahel. Environmental Management, 2009. 43(5): p. 804-816.

313. Mertz, O., et al., Adaptation strategies and climate vulnerability in the Sudano-Sahelian region of West Africa. Atmospheric Science Letters, 2011. 12(1): p. 104-108.

314. Mills, D.M., Climate Change, Extreme Weather Events, and US Health Impacts: What Can We Say? Journal of Occupational and Environmental Medicine, 2009. 51(1): p. 26-32.

315. Miranda, J.D., et al., Climatic change and rainfall patterns: Effects on semi-arid plant communities of the Iberian Southeast. Journal of Arid Environments, 2011. 75(12): p. 1302-1309.

316. Moench, M., Responding to climate and other change processes in complex contexts: Challenges facing development of adaptive policy frameworks in the Ganga Basin. Technological Forecasting and Social Change, 2010. 77(6): p. 975-986.

317. Moerlein, K.J. and C. Carothers, Total Environment of Change: Impacts of Climate Change and Social Transitions on Subsistence Fisheries in Northwest Alaska. Ecology and Society, 2012. 17(1).

318. Moller, H., et al., Guidelines for cross-cultural Participatory Action Research partnerships: a case study of a customary seabird harvest in New Zealand. New Zealand Journal of Zoology, 2009. 36(3): p. 211-241.

319. Molua, E.L., Accommodation of climate change in coastal areas of Cameroon: selection of household-level protection options. Mitigation and Adaptation Strategies for Global Change, 2009. 14(8): p. 721-735.

320. Molua, E.L., Farm income, gender differentials and climate risk in Cameroon: typology of male and female adaptation options across agroecologies. Sustainability Science, 2011. 6(1): p. 21-35.

321. Molua, E.L., Climate extremes, location vulnerability and private costs of property protection in Southwestern Cameroon. Mitigation and Adaptation Strategies for Global Change, 2012. 17(3): p. 293-310.

322. Montini, M., The Impact of International Treaties on Climate Change in SEE Countries, in Environmental Security in South-Eastern Europe: International Agreements and Their Implementation, M. Montini and S. Bogdanovic, Editors. 2009. p. 59-75.

323. Moser, S.C. and A.L. Luers, Managing climate risks in California: the need to engage resource managers for successful adaptation to change. Climatic Change, 2008. 87: p. S309-S322.

324. Motsholapheko, M.R., D.L. Kgathi, and C. Vanderpost, Rural livelihoods and household adaptation to extreme flooding in the Okavango Delta, Botswana. Physics and Chemistry of the Earth, 2011. 36(14-15): p. 984-995.

325. Mukheibir, P., Water resources management strategies for adaptation to climate-induced impacts in South Africa. Water Resources Management, 2008. 22(9): p. 1259-1276.

326. Munday, P.L., et al., Climate change and the future for coral reef fishes. Fish and Fisheries, 2008. 9(3): p. 261-285.

327. Mustelin, J., et al., Understanding current and future vulnerability in coastal settings: community perceptions and preferences for adaptation in Zanzibar, Tanzania. Population and Environment, 2010. 31(5): p. 371-398.

328. Mutunga, C. and K. Hardee, Population and reproductive health in National Adaptation Programmes of Action (NAPAs) for climate change in Africa. African journal of reproductive health, 2010. 14(4 Spec no.): p. 127-39.

329. Nabuurs, G.J., et al., Hotspots of the European forests carbon cycle. Forest Ecology and Management, 2008. 256(3): p. 194-200.

330. Nardone, A., et al., Effects of climate changes on animal production and sustainability of livestock systems. Livestock Science, 2010. 130(1-3): p. 57-69.

331. Nguyen, H., S.V.R.K. Prabhakar, and R. Shaw, Adaptive drought risk reduction in Cambodia: Reality, perceptions and strategies. Environmental Hazards-Human and Policy Dimensions, 2009. 8(4): p. 245-262.

332. Nicholas, K.A. and W.H. Durham, Farm-scale adaptation and vulnerability to environmental stresses: Insights from winegrowing in Northern California. Global Environmental Change-Human and Policy Dimensions, 2012. 22(2): p. 483-494.

333. Nielsen, D.L. and M.A. Brock, Modified water regime and salinity as a consequence of climate change: prospects for wetlands of Southern Australia. Climatic Change, 2009. 95(3-4): p. 523-533.

334. Nielsen, J.O. and A. Reenberg, Cultural barriers to climate change adaptation: A case study from Northern Burkina Faso. Global Environmental Change-Human and Policy Dimensions, 2010. 20(1): p. 142-152.

335. Noss, R.F., Between the devil and the deep blue sea: Florida's unenviable position with respect to sea level rise. Climatic Change, 2011. 107(1-2): p. 1-16.

336. Null, S.E., J.H. Viers, and J.F. Mount, Hydrologic Response and Watershed Sensitivity to Climate Warming in California's Sierra Nevada. Plos One, 2010. 5(3).

337. Nuorteva, P., M. Keskinen, and O. Varis, Water, livelihoods and climate change adaptation in the Tonle Sap Lake area, Cambodia: learning from the past to understand the future. Journal of Water and Climate Change, 2010. 1(1): p. 87-101.

338. Nurse, L.A., The implications of global climate change for fisheries management in the Caribbean. Climate and Development, 2011. 3(3): p. 228-241.

339. Nursey-Bray, M., Climate change adaptation in Australia Education, training and achieving social and political outcomes. International Journal of Climate Change Strategies and Management, 2010. 2(4): p. 393-402.

340. O'Neill, M.S., et al., US local action on heat and health: are we prepared for climate change? International Journal of Public Health, 2010. 55(2): p. 105-112.

341. Oberle, B. and B.A. Schaal, Responses to historical climate change identify contemporary threats to diversity in Dodecatheon. Proceedings of the National Academy of Sciences of the United States of America, 2011. 108(14): p. 5655-5660.

342. Ogden, A.E., Forest management in a changing climate: building the environmend information base for southwest Yukon. Forestry Chronicle, 2007. 83(6): p. 806-809.

343. Ogden, A.E. and J.L. Innes, Adapting to Climate Change in the Southwest Yukon: Locally Identified Research and Monitoring Needs to Support Decision Making on Sustainable Forest Management. Arctic, 2009. 62(2): p. 159-174.

344. Ogden, N.H., P. Sockett, and M. Fleury, Public Health in Canada and Adaptation to Infectious Disease Risks of Climate Change: Are We Planning or Just Keeping Our Fingers Crossed?, in Climate Change Adaptation in Developed Nations: From Theory to Practice, J.D. Ford and L. BerrangFord, Editors. 2011. p. 161-175.

345. Olesen, J.E., et al., Impacts and adaptation of European crop production systems to climate change. European Journal of Agronomy, 2011. 34(2): p. 96-112.

346. Oliver, T.S.N., et al., Measuring, mapping and modelling: an integrated approach to the management of mangrove and saltmarsh in the Minnamurra River estuary, southeast Australia. Wetlands Ecology and Management, 2012. 20(4): p. 353-371.

347. Olsson, L. and A. Jerneck, Farmers fighting climate change-from victims to agents in subsistence livelihoods. Wiley Interdisciplinary Reviews-Climate Change, 2010. 1(3): p. 363-373.

348. Oluoko-Odingo, A.A., Vulnerability and Adaptation to Food Insecurity and Poverty in Kenya. Annals of the Association of American Geographers, 2011. 101(1): p. 1-20.

349. Olwig, M.F., Multi-sited resilience: The mutual construction of "local" and "global" understandings and practices of adaptation and innovation. Applied Geography, 2012. 33(1): p. 112-118.

350. Onta, N. and B.P. Resurreccion, The Role of Gender and Caste in Climate Adaptation Strategies in Nepal Emerging Change and Persistent Inequalities in the Far-Western Region. Mountain Research and Development, 2011. 31(4): p. 351-356.

351. Onyekuru, N.A. and R. Marchant, Nigeria's Response to the Impacts of Climate Change: Developing Resilient and Ethical Adaptation Options. Journal of Agricultural & Environmental Ethics, 2012. 25(4): p. 585-595.

352. Ouedraogo, M., Y. Dembele, and L. Some, Farmer perceptions and adaptation options to rainfall change: Evidence from Burkina Faso. Secheresse (Montrouge), 2010. 21(2): p. 87-96.

353. Ounvichit, T., Equal water sharing in scarcity conditions: the case of the Chaisombat Muang Fai Irrigation System in Thailand. Paddy and Water Environment, 2011. 9(3): p. 325-332.

354. Paavola, J., Livelihoods, vulnerability and adaptation to climate change in Morogoro, Tanzania. Environmental Science & Policy, 2008. 11(7): p. 642-654.

355. Pandey, R. and S. Jha, Climate vulnerability index - measure of climate change vulnerability to communities: a case of rural Lower Himalaya, India. Mitigation and Adaptation Strategies for Global Change, 2012. 17(5): p. 487-506.

356. Paolisso, M., et al., Climate Change, Justice, and Adaptation among African American Communities in the Chesapeake Bay Region. Weather Climate and Society, 2012. 4(1): p. 34-47.

357. Pareek, A. and P.C. Trivedi, Cultural values and indigenous knowledge of climate change and disaster prediction in Rajasthan, India. Indian Journal of Traditional Knowledge, 2011. 10(1): p. 183-189.

358. Park, S., M. Howden, and S. Crimp, Informing regional level policy development and actions for increased adaptive capacity in rural livelihoods. Environmental Science & Policy, 2012. 15(1): p. 23-37.

359. Parkinson, R.W. and T. McCue, Assessing municipal vulnerability to predicted sea level rise: City of Satellite Beach, Florida. Climatic Change, 2011. 107(1-2): p. 203-223.

360. Patt, A., et al., Making index insurance attractive to farmers. Mitigation and Adaptation Strategies for Global Change, 2009. 14(8): p. 737-753.

361. Patt, A.G. and D. Schroeter, Perceptions of climate risk in Mozambique: Implications for the success of adaptation strategies. Global Environmental Change-Human and Policy Dimensions, 2008. 18(3): p. 458-467.

362. Pearce, T., et al., Advancing adaptation planning for climate change in the Inuvialuit Settlement Region (ISR): a review and critique. Regional Environmental Change, 2011. 11(1): p. 1-17.

363. Pearce, T., et al., Inuit vulnerability and adaptive capacity to climate change in Ulukhaktok, Northwest Territories, Canada. Polar Record, 2010. 46(237): p. 157-177.

364. Pearce, T.D., et al., Community collaboration and climate change research in the Canadian Arctic. Polar Research, 2009. 28(1): p. 10-27.

365. Pearsall, H., Linking the stressors and stressing the linkages: Human-environment vulnerability and brownfield redevelopment in New York City. Environmental Hazards-Human and Policy Dimensions, 2009. 8(2): p. 117-132.

366. Peras, R.J.J., et al., Climate Variability and Extremes in the Pantabangan-Carranglan Watershed, Philippines: Assessment of Impacts and Adaptation Practices. Journal of Environmental Science and Management, 2008. 11(2): p. 14-31.

367. Perez, R.T., A Community-Based Flood Risk Management in the Lower Pampanga River Basin. Journal of Environmental Science and Management, 2008. 11(1): p. 56-63.

368. Petheram, L., et al., 'Strange changes': Indigenous perspectives of climate change and adaptation in NE Arnhem Land (Australia). Global Environmental Change-Human and Policy Dimensions, 2010. 20(4): p. 681-692.

369. Phillips, S.J. and M. Dudik, Modeling of species distributions with Maxent: new extensions and a comprehensive evaluation. Ecography, 2008. 31(2): p. 161-175.

370. Picketts, I.M., J. Curry, and E. Rapaport, Community Adaptation to Climate Change: Environmental Planners' Knowledge and Experiences in British Columbia, Canada. Journal of Environmental Policy & Planning, 2012. 14(2): p. 119-137.

371. Piko, B.F. and M.A. Balazs, Authoritative parenting style and adolescent smoking and drinking. Addictive Behaviors, 2012. 37(3): p. 353-356.

372. Pinkerton, K.E., et al., An official American Thoracic Society workshop report: Climate change and human health. Proceedings of the American Thoracic Society, 2012. 9(1): p. 3-8.

373. Plaganyi, E.E., et al., Modelling climate-change effects on Australian and Pacific aquatic ecosystems: a review of analytical tools and management implications. Marine and Freshwater Research, 2011. 62(9): p. 1132-1147.

374. Polack, E., A Right to Adaptation: Securing the Participation of Marginalised Groups. Ids Bulletin-Institute of Development Studies, 2008. 39(4): p. 16-+.

375. Pollino, C.A., et al., Alternative System Views of Climate Change in the Central West of New South Wales (Australia). Modsim 2007: International Congress on Modelling and Simulation: Land, Water and Environmental Management: Integrated Systems for Sustainability, ed. L. Oxley and D. Kulasiri. 2007. 643-+.

376. Porio, E., Vulnerability, Adaptation, and Resilience to Floods and Climate Change-Related Risks among Marginal, Riverine Communities in Metro Manila. Asian Journal of Social Science, 2011. 39(4): p. 425-445.

377. Poulter, B., et al., Robust dynamics of Amazon dieback to climate change with perturbed ecosystem model parameters. Global Change Biology, 2010. 16(9): p. 2476-2495.

378. Prabhakar, S.V.R.K. and R. Shaw, Climate change adaptation implications for drought risk mitigation: a perspective for India. Climatic Change, 2008. 88(2): p. 113-130.

379. Prno, J., et al., Community vulnerability to climate change in the context of other exposure-sensitivities in Kugluktuk, Nunavut. Polar Research, 2011. 30.

380. Proust, K., et al., Human Health and Climate Change: Leverage Points for Adaptation in Urban Environments. International Journal of Environmental Research and Public Health, 2012. 9(6): p. 2134-2158.

381. Przeslawski, R., et al., Beyond corals and fish: the effects of climate change on noncoral benthic invertebrates of tropical reefs. Global Change Biology, 2008. 14(12): p. 2773-2795.

382. Quinn, C.H., et al., Coping with Multiple Stresses in Rural South Africa. Ecology and Society, 2011. 16(3).

383. Rahim, M.A., et al., Underutilized Fruits and Vegetables in Bangladesh: Contribution to the National Economy, Poverty Reduction, Household Food Security and Nutrition, in International Symposium on Underutilized Plants for Food Security, Nutrition, Income and Sustainable Development, H. Jaenicke, et al., Editors. 2009. p. 423-428.

384. Rajmis, S., J. Barkmann, and R. Marggraf, User community preferences for climate change mitigation and adaptation measures around Hainich National Park, Germany. Climate Research, 2009. 40(1): p. 61-73.

385. Raphael, B., et al., Factors associated with population risk perceptions of continuing drought in Australia. Australian Journal of Rural Health, 2009. 17(6): p. 330-337.

386. Rasmussen, K., et al., Climate change on three Polynesian outliers in the Solomon Islands: Impacts, vulnerability and adaptation. Geografisk Tidsskrift-Danish Journal of Geography, 2009. 109(1): p. 1-13.

387. Ravera, F., D. Tarrason, and E. Simelton, Envisioning Adaptive Strategies to Change: Participatory Scenarios for Agropastoral Semiarid Systems in Nicaragua. Ecology and Society, 2011. 16(1).

388. Rawlani, A.K. and B.K. Sovacool, Building responsiveness to climate change through community based adaptation in Bangladesh. Mitigation and Adaptation Strategies for Global Change, 2011. 16(8): p. 845-863.

389. Reenberg, A., et al., Adaptation of Human Coping Strategies in a Small Island Society in the SW Pacific-50 Years of Change in the Coupled Human-Environment System on Bellona, Solomon Islands. Human Ecology, 2008. 36(6): p. 807-819.

390. Reisinger, A., et al., The Role of Local Government in Adapting to Climate Change: Lessons from New Zealand, in Climate Change Adaptation in Developed Nations: From Theory to Practice, J.D. Ford and L. BerrangFord, Editors. 2011. p. 303-319.

391. Reser, J.P. and J.K. Swim, Adapting to and Coping With the Threat and Impacts of Climate Change. American Psychologist, 2011. 66(4): p. 277-289.

392. Richardson, M.J., P. English, and L. Rudolph, A health impact assessment of California's proposed cap-and-trade regulations. American Journal of Public Health, 2012. 102(9): p. e52-8.

393. Riegl, B.M., et al., Present Limits to Heat-Adaptability in Corals and Population-Level Responses to Climate Extremes. PloS one, 2011. 6(9).

394. Roberts, D., Prioritizing climate change adaptation and local level resilience in Durban, South Africa. Environment and Urbanization, 2010. 22(2): p. 397-413.

395. Roberts, D., et al., Exploring ecosystem-based adaptation in Durban, South Africa: "learning-by-doing" at the local government coal face. Environment and Urbanization, 2012. 24(1): p. 167-195.

396. Rocklov, J. and B. Forsberg, The effect of temperature on mortality in Stockholm 1998-2003: A study of lag structures and heatwave effects. Scandinavian Journal of Public Health, 2008. 36(5): p. 516-523.

397. Rodima-Taylor, D., Social innovation and climate adaptation: Local collective action in diversifying Tanzania. Applied Geography, 2012. 33(1): p. 128-134.

398. Rodriguez, D., et al., The intrinsic plasticity of farm businesses and their resilience to change. An Australian example. Field Crops Research, 2011. 124(2): p. 157-170.

399. Rodriguez-Gonzalez, P.M., et al., Subsidy or stress? Tree structure and growth in wetland forests along a hydrological gradient in Southern Europe. Forest Ecology and Management, 2010. 259(10): p. 2015-2025.

400. Rogers, J.D., et al., Modeling scale and variability in human-environmental interactions in Inner Asia. Ecological Modelling, 2012. 241: p. 5-14.

401. Roiko, A., et al., Socio-economic trends and climate change adaptation: the case of South East Queensland. Australasian Journal of Environmental Management, 2012. 19(1): p. 35-50.

402. Romsdahl, R.J., Decision support for climate change adaptation planning in the US: why it needs a coordinated internet-based practitioners' network. Climatic Change, 2011. 106(4): p. 507-536.

403. Roncoli, C., et al., Cultural styles of participation in farmers' discussions of seasonal climate forecasts in Uganda. Agriculture and Human Values, 2011. 28(1): p. 123-138.

404. Rosen, L., et al., A framework for developing an evidence-based, comprehensive tobacco control program. Health Research Policy and Systems, 2010. 8.

405. Rosenthal, J.P. and C.M. Jessup, Global climate change and health: developing a research agenda for the NIH. Transactions of the American Clinical and Climatological Association, 2009. 120: p. 129-41.

406. Rotberg, F.J.Y., Social networks and adaptation in rural Bangladesh. Climate and Development, 2010. 2(1): p. 65-72.

407. Roy, M., Planning for sustainable urbanisation in fast growing cities: Mitigation and adaptation issues addressed in Dhaka, Bangladesh. Habitat International, 2009. 33(3): p. 276-286.

408. Ruiz-Ballesteros, E., Social-ecological resilience and community-based tourism An approach from Agua Blanca, Ecuador. Tourism Management, 2011. 32(3): p. 655-666.

409. Saavedra, C. and W.W. Budd, Climate change and environmental planning: Working to build community resilience and adaptive capacity in Washington State, USA. Habitat International, 2009. 33(3): p. 246-252.

410. Safi, A.S., W.J. Smith, Jr., and Z. Liu, Rural Nevada and Climate Change: Vulnerability, Beliefs, and Risk Perception. Risk Analysis, 2012. 32(6): p. 1041-1059.

411. Sales, R.F.M., Jr., Vulnerability and adaptation of coastal communities to climate variability and sea-level rise: Their implications for integrated coastal management in Cavite City, Philippines. Ocean & Coastal Management, 2009. 52(7): p. 395-404.

412. Salewski, V., W.M. Hochachka, and W. Fiedler, Global warming and Bergmann's rule: do central European passerines adjust their body size to rising temperatures? Oecologia, 2010. 162(1): p. 247-260.

413. Sano, M., et al., A detailed assessment of vulnerability to climate change in the Gold Coast, Australia. Journal of Coastal Research, 2011: p. 245-249.

414. Saroar, M.M. and J.K. Routray, Impacts of climatic disasters in coastal Bangladesh: why does private adaptive capacity differ? Regional Environmental Change, 2012. 12(1): p. 169-190.

415. Sato, M. and M. Seki, Sustainable Business, Sustainable Planet-A Japanese Insurance Perspective. Geneva Papers on Risk and Insurance-Issues and Practice, 2010. 35(2): p. 325-335.

416. Sawyer, M.G., et al., School-Based Prevention of Depression: A 2-Year Follow-up of a Randomized Controlled Trial of the beyondblue Schools Research Initiative. Journal of Adolescent Health, 2010. 47(3): p. 297-304.

417. Sayegh, M.A., et al., Teen Pregnancy in Texas: 2005 to 2015. Maternal and Child Health Journal, 2010. 14(1): p. 94-101.

418. Scally, J. and G. Wescott, Perceptions of Climate Change and Adaptation Responses in a Local Community: the Barwon Estuary Complex, Victoria. Australian Geographer, 2011. 42(4): p. 387-401.

419. Scheffran, J., E. Marmer, and P. Sow, Migration as a contribution to resilience and innovation in climate adaptation: Social networks and co-development in Northwest Africa. Applied Geography, 2012. 33(1): p. 119-127.

420. Schwartze, F., et al., Research News for Climate Compliant Cities: The Case of Ho Chi Minh City, Vietnam. Resilient Cities: Cities and Adaptation to Climate Change - Proceedings of the Global Forum 2010, ed. K. OttoZimmermann. Vol. 1. 2011. 339-348.

421. Schwarz, A.-M., et al., Vulnerability and resilience of remote rural communities to shocks and global changes: Empirical analysis from Solomon Islands. Global Environmental Change-Human and Policy Dimensions, 2011. 21(3): p. 1128-1140.

422. Schwinning, S., et al., Sensitivity of the Colorado Plateau to Change: Climate, Ecosystems, and Society. Ecology and Society, 2008. 13(2).

423. Scott, D., M.C. Simpson, and R. Sim, The vulnerability of Caribbean coastal tourism to scenarios of climate change related sea level rise. Journal of Sustainable Tourism, 2012. 20(6): p. 883-898.

424. Seo, S.N. and R. Mendelsohn, A Ricardian analysis of the impact of climate change on South American farms. Chilean Journal of Agricultural Research, 2008. 68(1): p. 69-79.

425. Shannon, M.W., et al., Global climate change and children's health. Pediatrics, 2007. 120(5): p. 1149-1152.

426. Sharabi, A., U. Levi, and M. Margalit, Children's Loneliness, Sense of Coherence, Family Climate, and Hope: Developmental Risk and Protective Factors. Journal of Psychology, 2012. 146(1-2): p. 61-83.

427. Shepard, C.C., et al., Assessing future risk: quantifying the effects of sea level rise on storm surge risk for the southern shores of Long Island, New York. Natural Hazards, 2012. 60(2): p. 727-745.

428. Shephard, R.J. and Y. Aoyagi, Seasonal variations in physical activity and implications for human health. European Journal of Applied Physiology, 2009. 107(3): p. 251-271.

429. Sherman, C.D.H., et al., Development of twenty-three novel microsatellite markers for the seagrass, Zostera muelleri from Australia. Conservation Genetics Resources, 2012. 4(3): p. 689-693.

430. Siegel, P.B., J. Gatsinzi, and A. Kettlewell, Adaptive Social Protection in Rwanda: 'Climate-proofing' the Vision 2020 Umurenge Programme. Ids Bulletin-Institute of Development Studies, 2011. 42(6): p. 71-78.

431. Sietz, D., S.E. Mamani Choque, and M.K.B. Luedeke, Typical patterns of smallholder vulnerability to weather extremes with regard to food security in the Peruvian Altiplano. Regional Environmental Change, 2012. 12(3): p. 489-505.

432. Silva, J.A., S. Eriksen, and Z.A. Ombe, Double exposure in Mozambique's Limpopo River Basin. Geographical Journal, 2010. 176: p. 6-24.

433. Silvia Sanchez-Cortes, M. and E. Lazos Chavero, Indigenous perception of changes in climate variability and its relationship with agriculture in a Zoque community of Chiapas, Mexico. Climatic Change, 2011. 107(3-4): p. 363-389.

434. Simoes, A.F., et al., Enhancing adaptive capacity to climate change: The case of smallholder farmers in the Brazilian semi-arid region. Environmental Science & Policy, 2010. 13(8): p. 801-808.

435. Sissoko, K., et al., Agriculture, livelihoods and climate change in the West African Sahel. Regional Environmental Change, 2011. 11: p. S119-S125.

436. Slegers, M.F.W., "If only it would rain": Farmers' perceptions of rainfall and drought in semi-arid central Tanzania. Journal of Arid Environments, 2008. 72(11): p. 2106-2123.

437. Smith, J.-A.M., M. Mulligan, and Y. Nadarajah, Scenarios for Engaging a Rural Australian Community in Climate Change Adaptation Work, in Climate Change Adaptation in Developed Nations: From Theory to Practice, J.D. Ford and L. BerrangFord, Editors. 2011. p. 413-422.

438. Smith, J.W., D.H. Anderson, and R.L. Moore, Social Capital, Place Meanings, and Perceived Resilience to Climate Change. Rural Sociology, 2012. 77(3): p. 380-407.

439. Smith, J.W., et al., Community Resilience in Southern Appalachia: A Theoretical Framework and Three Case Studies. Human Ecology, 2012. 40(3): p. 341-353.

440. Smith, T.F., et al., Managing for Climate Variability in the Sydney Region. Journal of Coastal Research, 2007: p. 109-113.

441. Smith, T.F., D.C. Thomsen, and N. Keys, The Australian Experience, in Climate Change Adaptation in Developed Nations: From Theory to Practice, J.D. Ford and L. BerrangFord, Editors. 2011. p. 69-84.

442. Snell-Rood, E.C., Selective Processes in Development: Implications for the Costs and Benefits of Phenotypic Plasticity. Integrative and Comparative Biology, 2012. 52(1): p. 31-42.

443. Sonwa, D.J., et al., Building regional priorities in forests for development and adaptation to climate change in the Congo Basin. Mitigation and Adaptation Strategies for Global Change, 2012. 17(4): p. 441-450.

444. Sovacool, B.K., Expert views of climate change adaptation in the Maldives. Climatic Change, 2012. 114(2): p. 295-300.

445. Sovacool, B.K., et al., Improving climate change adaptation in least developed Asia. Environmental Science & Policy, 2012. 21: p. 112-125.

446. Spickett, J.T., H.L. Brown, and K. Rumchev, Climate Change and Air Quality: The Potential Impact on Health. Asia-Pacific Journal of Public Health, 2011. 23(2): p. 37S-45S.

447. Spielmann, K.A., et al., Sustainable Small-Scale Agriculture in Semi-Arid Environments. Ecology and Society, 2011. 16(1).

448. Spies, T.A., et al., Climate change adaptation strategies for federal forests of the Pacific Northwest, USA: ecological, policy, and socio-economic perspectives. Landscape Ecology, 2010. 25(8): p. 1185-1199.

449. Stage, J., Economic valuation of climate change adaptation in developing countries, in Ecological Economics Reviews, K. Limburg and R. Costanza, Editors. 2010. p. 150-163.

450. Stakhiv, E.Z., Pragmatic Approaches for Water Management Under Climate Change Uncertainty. Journal of the American Water Resources Association, 2011. 47(6): p. 1183-1196.

451. Steenberg, J.W.N., P.N. Duinker, and P.G. Bush, Exploring adaptation to climate change in the forests of central Nova Scotia, Canada. Forest Ecology and Management, 2011. 262(12): p. 2316-2327.

452. Storch, H. and N.K. Downes, A scenario-based approach to assess Ho Chi Minh City's urban development strategies against the impact of climate change. Cities, 2011. 28(6): p. 517-526.

453. Strand, L.B., et al., Vulnerability of eco-environmental health to climate change: the views of government stakeholders and other specialists in Queensland, Australia. Bmc Public Health, 2010. 10.

454. Stringer, L.C., et al., Adaptations to climate change, drought and desertification: local insights to enhance policy in southern Africa. Environmental Science & Policy, 2009. 12(7): p. 748-765.

455. Stringer, L.C., et al., Adaptation to climate change and desertification: Perspectives from national policy and autonomous practice in Malawi. Climate and Development, 2010. 2(2): p. 145-160.

456. Strous, R.D., et al., Confronting the bomber - Coping at the site of previous terror attacks. Journal of Nervous and Mental Disease, 2007. 195(3): p. 233-239.

457. Sultana, P. and P. Thompson, Local institutions for floodplain management in Bangladesh and the influence of the Flood Action Plan. Environmental Hazards-Human and Policy Dimensions, 2010. 9(1): p. 26-42.

458. Syal, S.S., et al., Climate change and human health-what influences the adoption of adaptation programming in the United States public health system? Mitigation and Adaptation Strategies for Global Change, 2011. 16(8): p. 911-924.

459. Tang, Z., et al., Surveying local planning directors' actions for climate change. International Journal of Climate Change Strategies and Management, 2012. 4(1): p. 81-103.

460. Tanner, T., Shifting the Narrative: Child-led Responses to Climate Change and Disasters in El Salvador and the Philippines. Children & Society, 2010. 24(4): p. 339-351.

461. Tarnoczi, T., Transformative learning and adaptation to climate change in the Canadian Prairie agro-ecosystem. Mitigation and Adaptation Strategies for Global Change, 2011. 16(4): p. 387-406.

462. Taylor, B.M., et al., Property Developers and Urban Adaptation: Conceptual and Empirical Perspectives on Governance. Urban Policy and Research, 2012. 30(1): p. 5-24.

463. Taylor, J., Community-Based Vulnerability Assessment: Semarang, Indonesia. Resilient Cities: Cities and Adaptation to Climate Change - Proceedings of the Global Forum 2010, ed. K. OttoZimmermann. Vol. 1. 2011. 329-337.

464. Thiel, M., et al., The Humboldt Current System of northern and central Chile, in Oceanography and Marine Biology, Vol 45, R.N. Gibson, R.J.A. Atkinson, and J.D.M. Gordon, Editors. 2007. p. 195-344.

465. Thom, B. and F. McKenzie, The Population Policy Debate from a Natural Resource Perspective: Reflections from the Wentworth Group. Geographical Research, 2011. 49(3): p. 348-361.

466. Thomalla, F., et al., Reducing hazard vulnerability: towards a common approach between disaster risk reduction and climate adaptation. Disasters, 2006. 30(1): p. 39-48.

467. Thomas, D.S.G., et al., Adaptation to climate change and variability: farmer responses to intra-seasonal precipitation trends in South Africa. Climatic Change, 2007. 83(3): p. 301-322.

468. Thornton, P.K., et al., Coping strategies in livestock-dependent households in east and southern Africa: A synthesis of four case studies. Human Ecology, 2007. 35(4): p. 461-476.

469. Thornton, P.K. and P.J. Gerber, Climate change and the growth of the livestock sector in developing countries. Mitigation and Adaptation Strategies for Global Change, 2010. 15(2): p. 169-184.

470. Thornton, P.K., et al., Agriculture and food systems in sub-Saharan Africa in a 4 degrees C+ world. Philosophical Transactions of the Royal Society a-Mathematical Physical and Engineering Sciences, 2011. 369(1934): p. 117-136.

471. Thorsen, S.M. and M. Hoglind, Assessing winter survival of forage grasses in Norway under future climate scenarios by simulating potential frost tolerance in combination with simple agroclimatic indices. Agricultural and Forest Meteorology, 2010. 150(9): p. 1272-1282.

472. Tingem, M., M. Rivington, and J. Colls, Climate variability and maize production in Cameroon: Simulating the effects of extreme dry and wet years. Singapore Journal of Tropical Geography, 2008. 29(3): p. 357-370.

473. Tockner, K., et al., Multiple stressors in coupled river-floodplain ecosystems. Freshwater Biology, 2010. 55: p. 135-151.

474. Tol, R.S.J., R.J.T. Klein, and R.J. Nicholls, Towards successful adaptation to sea-level rise along Europe's coasts. Journal of Coastal Research, 2008. 24(2): p. 432-442.

475. Tompkins, E.L., L.-A. Hurlston, and W. Poortinga, Foreignness as a constraint on learning: The impact of migrants on disaster resilience in small islands. Environmental Hazards-Human and Policy Dimensions, 2009. 8(4): p. 263-277.

476. Toni, F. and E. Holanda, Jr., The effects of land tenure on vulnerability to droughts in Northeastern Brazil. Global Environmental Change-Human and Policy Dimensions, 2008. 18(4): p. 575-582.

477. Tonn, B., The intergovernmental panel on climate change: A global scale transformative initiative. Futures, 2007. 39(5): p. 614-618.

478. Traerup, S.L.M. and O. Mertz, Rainfall variability and household coping strategies in northern Tanzania: a motivation for district-level strategies. Regional Environmental Change, 2011. 11(3): p. 471-481.

479. Traill, L.W., et al., Wetland conservation and sustainable use under global change: a tropical Australian case study using magpie geese. Ecography, 2010. 33(5): p. 818-825.

480. Tremblay, M., et al., Climate Change in Northern Quebec: Adaptation Strategies from Community-Based Research. Arctic, 2008. 61: p. 27-34.

481. Troccoli, A., Seasonal climate forecasting. Meteorological Applications, 2010. 17(3): p. 251-268.

482. Trotman, A., et al., Policy responses to GEC impacts on food availability and affordability in the Caribbean community. Environmental Science & Policy, 2009. 12(4): p. 529-541.

483. Tryhorn, L.M. and A.H. Lynch, Climate change adaptation in the Alpine Shire of Australia: a decision process appraisal. Policy Sciences, 2010. 43(2): p. 105-127.

484. Turner, R.A., et al., Declining reliance on marine resources in remote South Pacific societies: ecological versus socio-economic drivers. Coral Reefs, 2007. 26(4): p. 997-1008.

485. Twomlow, S., et al., Building adaptive capacity to cope with increasing vulnerability due to climatic change in Africa - A new approach. Physics and Chemistry of the Earth, 2008. 33(8-13): p. 780-787.

486. Valdivia, C., et al., Adapting to Climate Change in Andean Ecosystems: Landscapes, Capitals, and Perceptions Shaping Rural Livelihood Strategies and Linking Knowledge Systems. Annals of the Association of American Geographers, 2010. 100(4): p. 818-834.

487. van der Merwe, P., A school-based socio-emotional programme as strategy against crime and violence. Tydskrif Vir Geesteswetenskappe, 2011. 51(3): p. 388-402.

488. van Garderen, E.R.M.A., (Re) Considering Cattle Farming in Southern Africa under a Changing Climate. Weather Climate and Society, 2011. 3(4): p. 249-253.

489. van Pelt, S.C. and R.J. Swart, Climate Change Risk Management in Transnational River Basins: The Rhine. Water Resources Management, 2011. 25(14): p. 3837-3861.

490. van Straaten, O., et al., Spatial and temporal effects of drought on soil CO2 efflux in a cacao agroforestry system in Sulawesi, Indonesia. Biogeosciences, 2010. 7(4): p. 1223-1235.

491. van Vliet, N., Participatory Vulnerability Assessment in the Context of Conservation and Development Projects: A Case Study of Local Communities in Southwest Cameroon. Ecology and Society, 2010. 15(2).

492. Vanhala, P., et al., Temperature sensitivity of soil organic matter decomposition in southern and northern areas of the boreal forest zone. Soil Biology & Biochemistry, 2008. 40(7): p. 1758-1764.

493. Venkateswarlu, B. and A.K. Shanker, Dryland Agriculture: Bringing Resilience to Crop Production Under Changing Climate. Crop Stress and its Management: Perspectives and Strategies, ed. B. Venkateswarlu, et al. 2012. 19-44.

494. Vogel, C., I. Koch, and K. Van Zyl, "A Persistent Truth"-Reflections on Drought Risk Management in Southern Africa. Weather Climate and Society, 2010. 2(1): p. 9-22.

495. von Glasenapp, M. and T.F. Thornton, Traditional Ecological Knowledge of Swiss Alpine Farmers and their Resilience to Socioecological Change. Human Ecology, 2011. 39(6): p. 769-781.

496. Walker, R. and S.E.H.C. Partnership, Climate change and primary health care intervention framework. Australian Journal of Primary Health, 2009. 15(4): p. 276-284.

497. Wamsler, C., E. Brink, and O. Rentala, Climate Change, Adaptation, and Formal Education: the Role of Schooling for Increasing Societies' Adaptive Capacities in El Salvador and Brazil. Ecology and Society, 2012. 17(2).

498. Wamsler, C. and N. Lawson, Complementing institutional with localised strategies for climate change adaptation: a South-North comparison. Disasters, 2012. 36(1): p. 28-53.

499. Wandiga, S.O., et al., Vulnerability to epidemic malaria in the highlands of Lake Victoria basin: the role of climate change/variability, hydrology and socio-economic factors. Climatic Change, 2010. 99(3-4): p. 473-497.

500. Wang, C. and B. Yarnal, The vulnerability of the elderly to hurricane hazards in Sarasota, Florida. Natural Hazards, 2012. 63(2): p. 349-373.

501. Warren, R., et al., European drought regimes under mitigated and unmitigated climate change: application of the Community Integrated Assessment System (CIAS). Climate Research, 2012. 51(2): p. 105-U37.

502. Watt, S. and J. Chamberlain, Water, climate change, and maternal and newborn health. Current Opinion in Environmental Sustainability, 2011. 3(6): p. 491-496.

503. Wesche, S., et al., Community-based health research led by the Vuntut Gwitchin First Nation. International Journal of Circumpolar Health, 2011. 70(4): p. 396-406.

504. Wesche, S.D. and H.M. Chan, Adapting to the Impacts of Climate Change on Food Security among Inuit in the Western Canadian Arctic. Ecohealth, 2010. 7(3): p. 361-373.

505. West, C.T., The survey of living conditions in the Arctic (SLiCA): A comparative sustainable livelihoods assessment. Environment Development and Sustainability, 2011. 13(1): p. 217-235.

506. West, C.T., C. Roncoli, and F. Ouattara, Local perceptions and regional climate trends on the central plateau of Burkina Faso. Land Degradation & Development, 2008. 19(3): p. 289-304.

507. Westerhoff, L. and B. Smit, The rains are disappointing us: dynamic vulnerability and adaptation to multiple stressors in the Afram Plains, Ghana. Mitigation and Adaptation Strategies for Global Change, 2009. 14(4): p. 317-337.

508. Wheaton, E., et al., Dry times: hard lessons from the Canadian drought of 2001 and 2002. Canadian Geographer-Geographe Canadien, 2008. 52(2): p. 241-262.

509. Whittaker, J., J. Handmer, and D. Mercer, Vulnerability to bushfires in rural Australia: A case study from East Gippsland, Victoria. Journal of Rural Studies, 2012. 28(2): p. 161-173.

510. Wilbanks, T.J. and R.W. Kates, Beyond Adapting to Climate Change: Embedding Adaptation in Responses to Multiple Threats and Stresses. Annals of the Association of American Geographers, 2010. 100(4): p. 719-728.

511. Willox, A.C., et al., "From this place and of this place": Climate change, sense of place, and health in Nunatsiavut, Canada. Social Science & Medicine, 2012. 75(3): p. 538-547.

512. Wolfe, D.W., et al., Projected change in climate thresholds in the Northeastern US: implications for crops, pests, livestock, and farmers. Mitigation and Adaptation Strategies for Global Change, 2008. 13(5-6): p. 555-575.

513. Wong, P.P., Small island developing states. Wiley Interdisciplinary Reviews-Climate Change, 2011. 2(1): p. 1-6.

514. Woodruff, R.E., et al., Action on climate change: the health risks of procrastinating. Australian and New Zealand Journal of Public Health, 2006. 30(6): p. 567-571.

515. Yamane, A., Climate change and hazardscape of Sri Lanka. Environment and Planning A, 2009. 41(10): p. 2396-2416.

516. Yoon, H.R., F. Rivas, and W.L. Filho, An Assessment of Adaptation Strategies in the Baltic Sea Region: A Two-Country Analysis, in Economic, Social and Political Elements of Climate Change, W.L. Filho, Editor. 2011. p. 337-354.

517. Young, G., et al., Vulnerability and adaptation in a dryland community of the Elqui Valley, Chile. Climatic Change, 2010. 98(1-2): p. 245-276.

518. Yu, X., et al., Freshwater management and climate change adaptation: Experiences from the Central Yangtze in China. Climate and Development, 2009. 1(3): p. 241-248.

519. Zachara, T., Changes in the concept of improvement felling in respect of forest management objective. Quo Vadis, Forestry?, Proceedings, ed. Z. Sierota. 2007. 449-454.

520. Zamin, T.J., et al., National Red Listing Beyond the 2010 Target. Conservation Biology, 2010. 24(4): p. 1012-1020.

521. Zanabria-Salcedo, M., et al., One-year internal consistency of the Infant/Toddler HOME Inventory in a low risk infant group of Mexico City and its metropolitan zone. Salud Mental, 2007. 30(2): p. 67-73.

522. Zanobetti, A., et al., Summer temperature variability and long-term survival among elderly people with chronic disease. Proceedings of the National Academy of Sciences of the United States of America, 2012. 109(17): p. 6608-6613.

523. Zhou, H., et al., Resilience to natural hazards: a geographic perspective. Natural Hazards, 2010. 53(1): p. 21-41.

524. Ziervogel, G. and F. Zermoglio, Climate change scenarios and the development of adaptation strategies in Africa: challenges and opportunities. Climate Research, 2009. 40(2-3): p. 133-146.

**Mitigation (n=24)**

1. Alberini, A. and A. Chiabai, Urban environmental health and sensitive populations: How much are the Italians willing to pay to reduce their risks? Regional Science and Urban Economics, 2007. 37(2): p. 239-258.

2. Bassett, E. and V. Shandas, Innovation and Climate Action Planning. Journal of the American Planning Association, 2010. 76(4): p. 435-450.

3. Belanger, D., et al., Use of a Remote Car Starter in Relation to Smog and Climate Change Perceptions: A Population Survey in Quebec (Canada). International Journal of Environmental Research and Public Health, 2009. 6(2): p. 694-709.

4. Botzen, W.J.W., J.C.J.H. Aerts, and J.C.J.M. van den Bergh, Willingness of homeowners to mitigate climate risk through insurance. Ecological Economics, 2009. 68(8-9): p. 2265-2277.

5. Bradford, J.B. and D.N. Kastendick, Age-related patterns of forest complexity and carbon storage in pine and aspen-birch ecosystems of northern Minnesota, USA. Canadian Journal of Forest Research-Revue Canadienne De Recherche Forestiere, 2010. 40(3): p. 401-409.

6. Brody, S., H. Grover, and A. Vedlitz, Examining the willingness of Americans to alter behaviour to mitigate climate change. Climate Policy, 2012. 12(1): p. 1-22.

7. Buechs, M., N. Bardsley, and S. Duwe, Who bears the brunt? Distributional effects of climate change mitigation policies. Critical Social Policy, 2011. 31(2): p. 285-307.

8. Bueno, P.B., et al., Wave of change: Coping with catastrophe, in Mitigating Impacts of Natural Hazards on Fishery Ecosystems, K.D. McLaughlin, Editor. 2008. p. 309-324.

9. Butt, T.E., et al., Advent of Climate Change and Resultant Energy Related Obsolescence in the Built Environment, in Sustainability in Energy and Buildings, R.J. Howlett, L.C. Jain, and S.H. Lee, Editors. 2011. p. 211-224.

10. Chavunduka, C. and D.W. Bromley, Climate, carbon, civil war and flexible boundaries: Sudan's contested landscape. Land Use Policy, 2011. 28(4): p. 907-916.

11. Davies, Z.G. and P.R. Armsworth, Making an impact: The influence of policies to reduce emissions from aviation on the business travel patterns of individual corporations. Energy Policy, 2010. 38(12): p. 7634-7638.

12. Doherty, S.J., et al., LESSONS LEARNED FROM IPCC AR4 Scientific Developments Needed To Understand, Predict, And Respond To Climate Change. Bulletin of the American Meteorological Society, 2009. 90(4): p. 497-+.

13. Ebohon, O.J. and A.J. Ikeme, Decomposition analysis of CO2 emission intensity between oilproducing and non-oll-producing sub-Saharan African countries. Energy Policy, 2006. 34(18): p. 3599-3611.

14. Fitton, N., et al., Greenhouse gas mitigation potential of agricultural land in Great Britain. Soil Use and Management, 2011. 27(4): p. 491-501.

15. Ford, J.D., W. Vanderbilt, and L. Berrang-Ford, Authorship in IPCC AR5 and its implications for content: climate change and Indigenous populations in WGII. Climatic Change, 2012. 113(2): p. 201-213.

16. Glemarec, Y., Financing the transition to a low-carbon society. Journal of Renewable and Sustainable Energy, 2010. 2(3).

17. Hertel, T.W. and S.D. Rosch, Climate Change, Agriculture, and Poverty. Applied Economic Perspectives and Policy, 2010. 32(3): p. 355-385.

18. Johnson, C.A. and K. Krishnamurthy, Dealing with displacement: Can "social protection" facilitate long-term adaptation to climate change? Global Environmental Change-Human and Policy Dimensions, 2010. 20(4): p. 648-655.

19. Leary, N., et al., Crossing thresholds in regional climate research: synthesis of the IPCC expert meeting on regional impacts, adaptation, vulnerability, and mitigation INTRODUCTION. Climate Research, 2009. 40(2-3): p. 121-131.

20. Lovell, H.C., Governing the carbon offset market. Wiley Interdisciplinary Reviews-Climate Change, 2010. 1(3): p. 353-362.

21. Magombeyi, M.S. and A.E. Taigbenu, Crop yield risk analysis and mitigation of smallholder farmers at quaternary catchment level: Case study of B72A in Olifants river basin, South Africa. Physics and Chemistry of the Earth, 2008. 33(8-13): p. 744-756.

22. Paeth, H. and C. Otto, The Population's View on Climate Change and Mitigation-Inferences for Media and Policy. Advanced Science Letters, 2009. 2(3): p. 310-318.

23. Ribarova, I., et al., Research-supported participatory planning for water stress mitigation. Journal of Environmental Planning and Management, 2011. 54(2): p. 283-300.

24. Semenza, J.C., G.B. Ploubidis, and L.A. George, Climate change and climate variability: personal motivation for adaptation and mitigation. Environmental Health, 2011. 10.

**Conceptual (n=145)**

1. Adger, W.N., et al., Resilience implications of policy responses to climate change. Wiley Interdisciplinary Reviews-Climate Change, 2011. 2(5): p. 757-766.

2. Adger, W.N., et al., Are there social limits to adaptation to climate change? Climatic Change, 2009. 93(3-4): p. 335-354.

3. Agrawala, S., et al., Incorporating climate change impacts and adaptation in environmental impact assessments: Opportunities and challenges. Climate and Development, 2012. 4(1): p. 26-39.

4. Alpas, H. and T. Kiymaz, Food Security and Eco-terrorism Impacts on Environmental Security Through Vulnerabilities, in Environmental Security in South-Eastern Europe: International Agreements and Their Implementation, M. Montini and S. Bogdanovic, Editors. 2009. p. 137-150.

5. Angeler, D.G., S. Drakare, and R.K. Johnson, Revealing the Organization of Complex Adaptive Systems through Multivariate Time Series Modeling. Ecology and Society, 2011. 16(3).

6. Auld, H., et al., Adaptation options for infrastructure under changing climate conditions. 2006 IEEE EIC Climate Change Conference, Vols 1 and 2. 2006. 657-667.

7. Beaumier, M.C. and J.D. Ford, Food Insecurity among Inuit Women Exacerbated by Socio-economic Stresses and Climate Change. Canadian Journal of Public Health-Revue Canadienne De Sante Publique, 2010. 101(3): p. 196-201.

8. Becker, A., et al., Climate change impacts on international seaports: knowledge, perceptions, and planning efforts among port administrators. Climatic Change, 2012. 110(1-2): p. 5-29.

9. Becker, P., The importance of integrating multiple administrative levels in capacity assessment for disaster risk reduction and climate change adaptation. Disaster Prevention and Management, 2012. 21(2): p. 226-233.

10. Below, T.B., et al., Can farmers' adaptation to climate change be explained by socio-economic household-level variables? Global Environmental Change-Human and Policy Dimensions, 2012. 22(1): p. 223-235.

11. Bender, S.O., Progress in natural hazard risk reduction: What hath development wrought? Environmental Hazards-Human and Policy Dimensions, 2011. 10(1): p. 69-79.

12. Bernier, E., et al., Easier surveillance of climate-related health vulnerabilities through a Web-based spatial OLAP application. International Journal of Health Geographics, 2009. 8.

13. Berry, H.L., K. Bowen, and T. Kjellstrom, Climate change and mental health: a causal pathways framework. International Journal of Public Health, 2010. 55(2): p. 123-132.

14. Biggs, D., et al., The implementation crisis in conservation planning: could "mental models" help? Conservation Letters, 2011. 4(3): p. 169-183.

15. Birkmann, J., et al., Adaptive urban governance: new challenges for the second generation of urban adaptation strategies to climate change. Sustainability Science, 2010. 5(2): p. 185-206.

16. Birkmann, J. and K. von Teichman, Integrating disaster risk reduction and climate change adaptation: key challenges-scales, knowledge, and norms. Sustainability Science, 2010. 5(2): p. 171-184.

17. Black, R., et al., The effect of environmental change on human migration. Global Environmental Change-Human and Policy Dimensions, 2011. 21: p. S3-S11.

18. Blennow, K. and J. Persson, Climate change: Motivation for taking measure to adapt. Global Environmental Change-Human and Policy Dimensions, 2009. 19(1): p. 100-104.

19. Botzen, W.J.W. and J.C.J.M. van den Bergh, Bounded Rationality, Climate Risks, and Insurance: Is There a Market for Natural Disasters? Land Economics, 2009. 85(2): p. 265-278.

20. Botzen, W.J.W. and J.C.J.M. Van den Bergh, Managing natural disaster risks in a changing climate. Environmental Hazards-Human and Policy Dimensions, 2009. 8(3): p. 209-225.

21. Bowen, K.J., et al., Governing for a Healthy Population: Towards an Understanding of How Decision-Making Will Determine Our Global Health in a Changing Climate. International Journal of Environmental Research and Public Health, 2012. 9(1): p. 55-72.

22. Boyd, E., et al., Exploring Development Futures in a Changing Climate: Frontiers for Development Policy and Practice. Development Policy Review, 2009. 27(6): p. 659-674.

23. Braman, L.M., P. Suarez, and M.K. van Aalst, Climate change adaptation: integrating climate science into humanitarian work. International Review of the Red Cross, 2010. 92(879): p. 693-712.

24. Brooks, S. and M. Loevinsohn, Shaping agricultural innovation systems responsive to food insecurity and climate change. Natural Resources Forum, 2011. 35(3): p. 185-200.

25. Brugmann, J., Financing the resilient city. Environment and Urbanization, 2012. 24(1): p. 215-232.

26. Castleden, M., et al., Resilience thinking in health protection. Journal of Public Health, 2011. 33(3): p. 369-377.

27. Chen, C.-C., B. McCarl, and C.-C. Chang, Climate change, sea level rise and rice: global market implications. Climatic Change, 2012. 110(3-4): p. 543-560.

28. Chen, X., et al., Adaptive and Bounded Investment Returns Promote Cooperation in Spatial Public Goods Games. PloS one, 2012. 7(5).

29. Chindarkar, N., Gender and climate change-induced migration: proposing a framework for analysis. Environmental Research Letters, 2012. 7(2).

30. Cohen, J.E., Population and Climate Change. Proceedings of the American Philosophical Society, 2010. 154(2): p. 158-182.

31. Coleman, E.A., Common property rights, adaptive capacity, and response to forest disturbance. Global Environmental Change-Human and Policy Dimensions, 2011. 21(3): p. 855-865.

32. Collier, B., J. Skees, and B. Barnett, Weather Index Insurance and Climate Change: Opportunities and Challenges in Lower Income Countries. Geneva Papers on Risk and Insurance-Issues and Practice, 2009. 34(3): p. 401-424.

33. Conlon, K.C., et al., Preventing cold-related morbidity and mortality in a changing climate. Maturitas, 2011. 69(3): p. 197-202.

34. Corfee-Morlot, J., et al., Multilevel risk governance and urban adaptation policy. Climatic Change, 2011. 104(1): p. 169-197.

35. Cousin, M.-E. and M. Siegrist, Risk perception of mobile communication: a mental models approach. Journal of Risk Research, 2010. 13(5): p. 599-620.

36. Crate, S.A., Gone the Bull of Winter? Grappling with the Cultural Implications of and Anthropology's Role(s) in Global Climate Change. Current Anthropology, 2008. 49(4): p. 569-595.

37. Cuevas, S.C., Climate change, vulnerability, and risk linkages. International Journal of Climate Change Strategies and Management, 2011. 3(1): p. 29-60.

38. Cutter, S.L., et al., A place-based model for understanding community resilience to natural disasters. Global Environmental Change-Human and Policy Dimensions, 2008. 18(4): p. 598-606.

39. David Tabara, J., et al., The Climate Learning Ladder. A Pragmatic Procedure to Support Climate Adaptation. Environmental Policy and Governance, 2010. 20(1): p. 1-11.

40. de Chazal, J., et al., Including multiple differing stakeholder values into vulnerability assessments of socio-ecological systems. Global Environmental Change-Human and Policy Dimensions, 2008. 18(3): p. 508-520.

41. de Vries, F.T., et al., Land use alters the resistance and resilience of soil food webs to drought. Nature Climate Change, 2012. 2(4): p. 276-280.

42. Deligiannis, T., The Evolution of Environment-Conflict Research: Toward a Livelihood Framework. Global Environmental Politics, 2012. 12(1): p. 78-+.

43. Dixon, M., Climate change, politics and the civil engineering profession. Proceedings of the Institution of Civil Engineers-Municipal Engineer, 2009. 162(4): p. 207-210.

44. Doherty, T.J. and S. Clayton, The Psychological Impacts of Global Climate Change. American Psychologist, 2011. 66(4): p. 265-276.

45. Dolan, A.H. and I.J. Walker, Understanding vulnerability of coastal communities to climate change related risks. Journal of Coastal Research, 2006: p. 1316-1323.

46. Engler, S., DEVELOPING A HISTORICALLY BASED "FAMINE VULNERABILITY ANALYSIS MODEL" (FVAM) - AN INTERDISCIPLINARY APPROACH. Erdkunde, 2012. 66(2): p. 157-172.

47. Fan, W., C.M. Williams, and D.M. Corkin, A MULTILEVEL ANALYSIS OF STUDENT PERCEPTIONS OF SCHOOL CLIMATE: THE EFFECT OF SOCIAL AND ACADEMIC RISK FACTORS. Psychology in the Schools, 2011. 48(6): p. 632-647.

48. Farbotko, C. and G. Waitt, Residential air-conditioning and climate change: voices of the vulnerable. Health Promotion Journal of Australia, 2011. 22: p. S13-S16.

49. Fazey, I., Resilience and Higher Order Thinking. Ecology and Society, 2010. 15(3).

50. Fieldman, G., Neoliberalism, the production of vulnerability and the hobbled state: Systemic barriers to climate adaptation. Climate and Development, 2011. 3(2): p. 159-174.

51. Fischer, A. and K. Glenk, One model fits all? On the moderating role of emotional engagement and confusion in the elicitation of preferences for climate change adaptation policies. Ecological Economics, 2011. 70(6): p. 1178-1188.

52. Folke, C., Resilience: The emergence of a perspective for social-ecological systems analyses. Global Environmental Change-Human and Policy Dimensions, 2006. 16(3): p. 253-267.

53. Ford, J.D., et al., Case study and analogue methodologies in climate change vulnerability research. Wiley Interdisciplinary Reviews-Climate Change, 2010. 1(3): p. 374-392.

54. Fuessel, H.-M., Vulnerability: A generally applicable conceptual framework for climate change research. Global Environmental Change-Human and Policy Dimensions, 2007. 17(2): p. 155-167.

55. Fuessel, H.-M., Assessing adaptation to the health risks of climate change: what guidance can existing frameworks provide? International Journal of Environmental Health Research, 2008. 18(1): p. 37-63.

56. Fussel, H.M. and R.J.T. Klein, Climate change vulnerability assessments: An evolution of conceptual thinking. Climatic Change, 2006. 75(3): p. 301-329.

57. Gifford, R., The Dragons of Inaction Psychological Barriers That Limit Climate Change Mitigation and Adaptation. American Psychologist, 2011. 66(4): p. 290-302.

58. Gill, M. and K. Johnston, Informing food policy: balancing the evidence. Proceedings of the Nutrition Society, 2010. 69(4): p. 621-627.

59. Gunasekara, N. and S. Kazama, Water conflict vulnerability of regions, in Risk in Water Resources Management, G. Bloschl, et al., Editors. 2011. p. 267-273.

60. Hahn, W.A. and T. Knoke, Sustainable development and sustainable forestry: analogies, differences, and the role of flexibility. European Journal of Forest Research, 2010. 129(5): p. 787-801.

61. Hartell, C.E.J. and G.I. Pearman, Understanding and responding to the climate change issue: Towards a whole-of-science research agenda. Journal of Management & Organization, 2010. 16(1): p. 16-47.

62. Harvey, B., D. Burns, and K. Oswald, Linking Community, Radio, and Action Research on Climate Change: Reflections on a Systemic Approach. Ids Bulletin-Institute of Development Studies, 2012. 43(3): p. 101-117.

63. Hasson, R., A. Lofgren, and M. Visser, Climate change in a public goods game Investment decision in mitigation versus adaptation. Ecological Economics, 2010. 70(2): p. 331-338.

64. Hecht, S.B., Climate change and the transformation of risk: Insurance matters. Ucla Law Review, 2008. 55(6): p. 1559-1620.

65. Heltberg, R., H. Gitay, and R.G. Prabhu, Community-based adaptation: lessons from a grant competition. Climate Policy, 2012. 12(2): p. 143-163.

66. Heltberg, R., P.B. Siegel, and S.L. Jorgensen, Addressing human vulnerability to climate change: Toward a 'no-regrets' approach. Global Environmental Change-Human and Policy Dimensions, 2009. 19(1): p. 89-99.

67. Henriques, C. and M. Fowler, Case study in resilience analysis and scheme selection. Integrating Water Systems, ed. J. Boxall and C. Maksimovic. 2010. 627-630.

68. Herman, P.F., Jr. and G.F. Treverton, The Political Consequences of Climate Change. Survival, 2009. 51(2): p. 137-147.

69. Hjerpe, M. and E. Glaas, Evolving local climate adaptation strategies: incorporating influences of socio-economic stress. Mitigation and Adaptation Strategies for Global Change, 2012. 17(5): p. 471-486.

70. Hoeppe, P. and E.N. Gurenko, Scientific and economic rationales for innovative climate insurance solutions. Climate Policy, 2006. 6(6): p. 607-620.

71. Hordijk, M. and I. Baud, Inclusive Adaptation: Linking Participatory Learning and Knowledge Management to Urban Resilience. Resilient Cities: Cities and Adaptation to Climate Change - Proceedings of the Global Forum 2010, ed. K. OttoZimmermann. Vol. 1. 2011. 111-121.

72. Hufschmidt, G., A comparative analysis of several vulnerability concepts. Natural Hazards, 2011. 58(2): p. 621-643.

73. Innocenti, D. and P. Albrito, Reducing the risks posed by natural hazards and climate change: the need for a participatory dialogue between the scientific community and policy makers. Environmental Science & Policy, 2011. 14(7): p. 730-733.

74. Ionescu, C., et al., Towards a Formal Framework of Vulnerability to Climate Change. Environmental Modeling & Assessment, 2009. 14(1): p. 1-16.

75. Jones, N., J. Clark, and G. Tripidaki, Social risk assessment and social capital: A significant parameter for the formation of climate change policies. Social Science Journal, 2012. 49(1): p. 33-41.

76. Karanja, D., S.J. Elliott, and S. Gabizon, Community level research on water health and global change: where have we been? Where are we going? Current Opinion in Environmental Sustainability, 2011. 3(6): p. 467-470.

77. Keim, M.E., Building Human Resilience The Role of Public Health Preparedness and Response As an Adaptation to Climate Change. American Journal of Preventive Medicine, 2008. 35(5): p. 508-516.

78. Keim, M.E., Preventing Disasters: Public Health Vulnerability Reduction as a Sustainable Adaptation to Climate Change. Disaster Medicine and Public Health Preparedness, 2011. 5(2): p. 140-148.

79. Keskitalo, E.C.H., Governance in vulnerability assessment: the role of globalising decision-making networks in determining local vulnerability and adaptive capacity. Mitigation and Adaptation Strategies for Global Change, 2009. 14(2): p. 185-201.

80. Kloeckner, C.A., Towards a Psychology of Climate Change, in Economic, Social and Political Elements of Climate Change, W.L. Filho, Editor. 2011. p. 153-173.

81. Kulshreshtha, S., E. Wheaton, and V. Wittrock, Natural hazards and First Nations community setting: challenges for adaptation, in Management of Natural Resources, Sustainable Development and Ecological Hazards Iii, C.A. Brebbia and S.S. Zubir, Editors. 2012. p. 277-288.

82. Kuruppu, N. and D. Liverman, Mental preparation for climate adaptation: The role of cognition and culture in enhancing adaptive capacity of water management in Kiribati. Global Environmental Change-Human and Policy Dimensions, 2011. 21(2): p. 657-669.

83. Langridge, R., J. Christian-Smith, and K.A. Lohse, Access and resilience: Analyzing the construction of social resilience to the threat of water scarcity. Ecology and Society, 2006. 11(2).

84. Lee, J.W.K., Getting Ready for a Changing Climate: Supporting Councillor's Leadership Role in Adaptation. Resilient Cities: Cities and Adaptation to Climate Change - Proceedings of the Global Forum 2010, ed. K. OttoZimmermann. Vol. 1. 2011. 141-148.

85. Leichenko, R., Climate change and urban resilience. Current Opinion in Environmental Sustainability, 2011. 3(3): p. 164-168.

86. Lemos, M.C., et al., Developing adaptation and adapting development. Ecology and Society, 2007. 12(2).

87. Lesnikowski, A.C., et al., Adapting to health impacts of climate change: a study of UNFCCC Annex I parties. Environmental Research Letters, 2011. 6(4).

88. Lin, B.B. and P.E. Morefield, The Vulnverability Cube: A Multi-Dimensional Framework for Assessing Relative Vulnerability. Environmental Management, 2011. 48(3): p. 631-643.

89. Lubchenco, J. and L.E. Petes, The Interconnected Biosphere: Science at the Ocean's Tipping Points. Oceanography, 2010. 23(2): p. 115-129.

90. MacDonald, A., The Rise of Sustainability: Changing Public Concerns and Governance Approaches toward Exploration, in Wealth Creation in the Minerals Industry: Integrating Science, Business, and Education, M.D. Doggett and J.R. Parry, Editors. 2006. p. 127-148.

91. Mahony, M. and M. Hulme, Model migrations: mobility and boundary crossings in regional climate prediction. Transactions of the Institute of British Geographers, 2012. 37(2): p. 197-211.

92. Maller, C.J. and Y. Strengers, Housing, heat stress and health in a changing climate: promoting the adaptive capacity of vulnerable households, a suggested way forward. Health Promotion International, 2011. 26(4): p. 492-498.

93. Manojlovic, N. and E. Pasche, Integration of resiliency measures into flood risk management concepts of communities, in Flood Recovery, Innovation and Response, D. Proverbs, C.A. Brebbia, and E. PenningRowsell, Editors. 2008. p. 235-245.

94. Matt, S.B. and P. Butterfield, Changing the disability climate: promoting tolerance in the workplace. AAOHN journal : official journal of the American Association of Occupational Health Nurses, 2006. 54(3): p. 129-33; quiz 134-5.

95. May, B. and R. Plummer, Accommodating the Challenges of Climate Change Adaptation and Governance in Conventional Risk Management: Adaptive Collaborative Risk Management (ACRM). Ecology and Society, 2011. 16(1).

96. McBean, G. and C. Rodgers, Climate hazards and disasters: the need for capacity building. Wiley Interdisciplinary Reviews-Climate Change, 2010. 1(6): p. 871-884.

97. McLaughlin, P., Climate Change, Adaptation, and Vulnerability: Reconceptualizing Societal-Environment Interaction Within a Socially Constructed Adaptive Landscape. Organization & Environment, 2011. 24(3): p. 269-291.

98. McLaughlin, P. and T. Dietz, Structure, agency and environment: Toward an integrated perspective on vulnerability. Global Environmental Change-Human and Policy Dimensions, 2008. 18(1): p. 99-111.

99. McLeman, R. and B. Smit, Migration as an adaptation to climate change. Climatic Change, 2006. 76(1-2): p. 31-53.

100. Measham, T.G., et al., Adapting to climate change through local municipal planning: barriers and challenges. Mitigation and Adaptation Strategies for Global Change, 2011. 16(8): p. 889-909.

101. Mitchell, T. and T. Tanner, Defining a Future Research Agenda on Pro-Poor Adaptation. Ids Bulletin-Institute of Development Studies, 2008. 39(4): p. 130-+.

102. Molnar, J.J., Climate Change and Societal Response: Livelihoods, Communities, and the Environment. Rural Sociology, 2010. 75(1): p. 1-16.

103. Moser, C. and A. Stein, Implementing urban participatory climate change adaptation appraisals: a methodological guideline. Environment and Urbanization, 2011. 23(2): p. 463-485.

104. Murphy, B.L., From interdisciplinary to inter-epistemological approaches: Confronting the challenges of integrated climate change research. Canadian Geographer-Geographe Canadien, 2011. 55(4): p. 490-509.

105. Murtinho, F. and T.M. Hayes, Adaptation in Resource-Dependent Communities: A Call for Greater Methodological Clarity in Adaptation Field Research. Society & Natural Resources, 2012. 25(5): p. 513-522.

106. Mustafa, D., et al., Pinning down vulnerability: from narratives to numbers. Disasters, 2011. 35(1): p. 62-86.

107. Nelson, D.R., Adaptation and resilience: responding to a changing climate. Wiley Interdisciplinary Reviews-Climate Change, 2011. 2(1): p. 113-120.

108. O'Neill, M.S., et al., Preventing heat-related morbidity and mortality: New approaches in a changing climate. Maturitas, 2009. 64(2): p. 98-103.

109. Palmer, M.A., et al., Climate Change and River Ecosystems: Protection and Adaptation Options. Environmental Management, 2009. 44(6): p. 1053-1068.

110. Park, S.E., et al., Informing adaptation responses to climate change through theories of transformation. Global Environmental Change-Human and Policy Dimensions, 2012. 22(1): p. 115-126.

111. Patwardhan, A., et al., Towards an integrated agenda for adaptation research: theory, practice and policy. Current Opinion in Environmental Sustainability, 2009. 1(2): p. 219-225.

112. Pitcher, H.M., The future of scenarios: issues in developing new climate change scenarios. Environmental Research Letters, 2009. 4(2).

113. Poulter, B., J.L. Goodall, and P.N. Halpin, Applications of network analysis for adaptive management of artificial drainage systems in landscapes vulnerable to sea level rise. Journal of Hydrology, 2008. 357(3-4): p. 207-217.

114. Prabhakar, S.V.R.K., A. Srinivasan, and R. Shaw, Climate change and local level disaster risk reduction planning: need, opportunities and challenges. Mitigation and Adaptation Strategies for Global Change, 2009. 14(1): p. 7-33.

115. Prato, T., Accounting for risk and uncertainty in determining preferred strategies for adapting to future climate change. Mitigation and Adaptation Strategies for Global Change, 2008. 13(1): p. 47-60.

116. Prowse, M. and L. Scott, Assets and Adaptation: An Emerging Debate. Ids Bulletin-Institute of Development Studies, 2008. 39(4): p. 42-+.

117. Pyke, C.R., et al., Development and analysis of Climate Sensitivity and Climate Adaptation opportunities indices for buildings. Building and Environment, 2012. 55: p. 141-149.

118. Reis, J. and G.C. Roman, Environmental neurology: A promising new field of practice and research. Journal of the Neurological Sciences, 2007. 262(1-2): p. 3-6.

119. Renaud, F.G., et al., Understanding multiple thresholds of coupled social-ecological systems exposed to natural hazards as external shocks. Natural Hazards, 2010. 55(3): p. 749-763.

120. Roman, C.E., A.H. Lynch, and D. Dominey-Howes, What is the Goal? Framing the Climate Change Adaptation Question through a Problem-Oriented Approach. Weather Climate and Society, 2011. 3(1): p. 16-30.

121. Ruth, M. and D. Coelho, Understanding and managing the complexity of urban systems under climate change. Climate Policy, 2007. 7(4): p. 317-336.

122. Sabates-Wheeler, R., T. Mitchell, and F. Ellis, Avoiding Repetition: Time for CBA to Engage with the Livelihoods Literature? Ids Bulletin-Institute of Development Studies, 2008. 39(4): p. 53-+.

123. Scott, D.J., C.J. Lemieux, and L. Malone, Climate services to support sustainable tourism and adaptation to climate change. Climate Research, 2011. 47(1-2): p. 111-122.

124. Smit, B. and J. Wandel, Adaptation, adaptive capacity and vulnerability. Global Environmental Change-Human and Policy Dimensions, 2006. 16(3): p. 282-292.

125. Smith, L.A. and N. Stern, Uncertainty in science and its role in climate policy. Philosophical Transactions of the Royal Society a-Mathematical Physical and Engineering Sciences, 2011. 369(1956): p. 4818-4841.

126. Smith, T.F., et al., A method for building community resilience to climate change in emerging coastal cities. Futures, 2011. 43(7): p. 673-679.

127. Sovacool, B.K., Hard and soft paths for climate change adaptation. Climate Policy, 2011. 11(4): p. 1177-1183.

128. Srinivasan, G., K.M. Rafisura, and A.R. Subbiah, Climate information requirements for community-level risk management and adaptation. Climate Research, 2011. 47(1-2): p. 5-12.

129. Stephenson, J., K. Newman, and S. Mayhew, Population dynamics and climate change: what are the links? Journal of Public Health, 2010. 32(2): p. 150-156.

130. Suarez, P., et al., Video-Mediated Approaches for Community-Level Climate Adaptation. Ids Bulletin-Institute of Development Studies, 2008. 39(4): p. 96-+.

131. Swart, R. and N. Marinova, Policy options in a worst case climate change world. Mitigation and Adaptation Strategies for Global Change, 2010. 15(6): p. 531-549.

132. Thomas, C.D., et al., A framework for assessing threats and benefits to species responding to climate change. Methods in Ecology and Evolution, 2011. 2(2): p. 125-142.

133. Tompkins, E.L. and H. Amundsen, Perceptions of the effectiveness of the United Nations Framework Convention on Climate Change in advancing national action on climate change. Environmental Science & Policy, 2008. 11(1): p. 1-13.

134. Tompkins, E.L. and H. Eakin, Managing private and public adaptation to climate change. Global Environmental Change-Human and Policy Dimensions, 2012. 22(1): p. 3-11.

135. van Aalst, M.K., T. Cannon, and I. Burton, Community level adaptation to climate change: The potential role of participatory community risk assessment. Global Environmental Change-Human and Policy Dimensions, 2008. 18(1): p. 165-179.

136. Vincent, K., Uncertainty in adaptive capacity and the importance of scale. Global Environmental Change-Human and Policy Dimensions, 2007. 17(1): p. 12-24.

137. Vogel, C., et al., Linking vulnerability, adaptation, and resilience science to practice: Pathways, players, and partnerships. Global Environmental Change-Human and Policy Dimensions, 2007. 17(3-4): p. 349-364.

138. Webster, P.J. and J. Jian, Environmental prediction, risk assessment and extreme events: adaptation strategies for the developing world. Philosophical Transactions of the Royal Society a-Mathematical Physical and Engineering Sciences, 2011. 369(1956): p. 4768-4797.

139. Wilby, R.L. and R. Keenan, Adapting to flood risk under climate change. Progress in Physical Geography, 2012. 36(3): p. 348-378.

140. Wilhelmi, O.V. and M.H. Hayden, Connecting people and place: a new framework for reducing urban vulnerability to extreme heat. Environmental Research Letters, 2010. 5(1).

141. Woodward, M., et al., Real Options in flood risk management decision making. Journal of Flood Risk Management, 2011. 4(4): p. 339-349.

142. Yohe, G., K. Knee, and P. Kirshen, On the economics of coastal adaptation solutions in an uncertain world. Climatic Change, 2011. 106(1): p. 71-92.

143. Yoo, G., J.H. Hwang, and C. Choi, Development and application of a methodology for vulnerability assessment of climate change in coastal cities. Ocean & Coastal Management, 2011. 54(7): p. 524-534.

144. Young, O.R., et al., The globalization of socio-ecological systems: An agenda for scientific research. Global Environmental Change-Human and Policy Dimensions, 2006. 16(3): p. 304-316.

145. Ziervogel, G. and P.J. Ericksen, Adapting to climate change to sustain food security. Wiley Interdisciplinary Reviews-Climate Change, 2010. 1(4): p. 525-540.

**Pre-Historic or Future-Orientated (n=46)**

1. Abuodha, P.A.O. and C.D. Woodroffe, Assessing vulnerability to sea-level rise using a coastal sensitivity index: a case study from southeast Australia. Journal of Coastal Conservation, 2010. 14(3): p. 189-205.

2. Acosta-Michlik, L. and V. Espaldon, Assessing vulnerability of selected farming communities in the Philippines based on a behavioural model of agent's adaptation to global environmental change. Global Environmental Change-Human and Policy Dimensions, 2008. 18(4): p. 554-563.

3. Albano Amora, S.S., et al., Monitoring of Lutzomyia longipalpis Lutz & Neiva, 1912 in an area of intense transmission of visceral leishmaniasis in Rio Grande do Norte, Northeast Brazil. Revista Brasileira De Parasitologia Veterinaria, 2010. 19(1): p. 39-43.

4. Auld, H., et al., Planning for atmospheric hazards and disaster management under changing climate conditions. 2006 IEEE EIC Climate Change Conference, Vols 1 and 2. 2006. 727-735.

5. Beier, C., et al., Precipitation manipulation experiments - challenges and recommendations for the future. Ecology Letters, 2012. 15(8): p. 899-911.

6. Carey, M., A. French, and E. O'Brien, Unintended effects of technology on climate change adaptation: an historical analysis of water conflicts below Andean Glaciers. Journal of Historical Geography, 2012. 38(2): p. 181-191.

7. Carey, M., et al., An integrated socio-environmental framework for glacier hazard management and climate change adaptation: lessons from Lake 513, Cordillera Blanca, Peru. Climatic Change, 2012. 112(3-4): p. 733-767.

8. Chevin, L.-M., R. Lande, and G.M. Mace, Adaptation, Plasticity, and Extinction in a Changing Environment: Towards a Predictive Theory. PLoS biology, 2010. 8(4).

9. Christierson, B.V., J.-P. Vidal, and S.D. Wade, Using UKCP09 probabilistic climate information for UK water resource planning. Journal of Hydrology, 2012. 424: p. 48-67.

10. Cloern, J.E., et al., Projected evolution of California's San Francisco Bay-Delta-river system in a century of climate change. PloS one, 2011. 6(9): p. e24465-e24465.

11. Coley, D., T. Kershaw, and M. Eames, A comparison of structural and behavioural adaptations to future proofing buildings against higher temperatures. Building and Environment, 2012. 55: p. 159-166.

12. Davies, R., The Red Store, Lerryn, Cornwall Its Adaptation Including Flood Mitigation Measures. Journal of Architectural Conservation, 2008. 14(3): p. 7-22.

13. Dewals, B.J., et al., Integrated assessment of flood protection measures in the context of climate change: hydraulic modelling and economic approach, in Environmental Economics and Investment Assessment Ii, K. Aravossis, C.A. Brebbia, and N. Gomez, Editors. 2008. p. 149-159.

14. Ebi, K.L., Healthy people 2100: modeling population health impacts of climate change. Climatic Change, 2008. 88(1): p. 5-19.

15. Endfield, G.H., Archival explorations of climate variability and social vulnerability in colonial Mexico. Climatic Change, 2007. 83(1-2): p. 9-38.

16. Fontaine, C.M. and M.D.A. Rounsevell, An agent-based approach to model future residential pressure on a regional landscape. Landscape Ecology, 2009. 24(9): p. 1237-1254.

17. Fraisse, C.W., et al., AgClimate: A climate forecast information system for agricultural risk management in the southeastern USA. Computers and Electronics in Agriculture, 2006. 53(1): p. 13-27.

18. Gallien, T.W., J.E. Schubert, and B.F. Sanders, Predicting tidal flooding of urbanized embayments: A modeling framework and data requirements. Coastal Engineering, 2011. 58(6): p. 567-577.

19. Gilligan, I., Neanderthal extinction and modern human behaviour: the role of climate change and clothing. World Archaeology, 2007. 39(4): p. 499-514.

20. Helbron, H., et al., Indicators for strategic environmental assessment in regional land use planning to assess conflicts with adaptation to global climate change. Ecological Indicators, 2011. 11(1): p. 90-95.

21. Hole, D.G., et al., Projected impacts of climate change on a continent-wide protected area network. Ecology Letters, 2009. 12(5): p. 420-431.

22. Jiang, L. and K. Hardee, How do Recent Population Trends Matter to Climate Change? Population Research and Policy Review, 2011. 30(2): p. 287-312.

23. Jung, I.W., H. Chang, and H. Moradkhani, Quantifying uncertainty in urban flooding analysis considering hydro-climatic projection and urban development effects. Hydrology and Earth System Sciences, 2011. 15(2): p. 617-633.

24. Kim, Y., et al., Different influence of outdoor temperature on traumatic and nontraumatic injuries. The journal of trauma and acute care surgery, 2012. 73(4): p. 944-9.

25. Klumpp, K., et al., Long-term impacts of agricultural practices and climatic variability on carbon storage in a permanent pasture. Global Change Biology, 2011. 17(12): p. 3534-3545.

26. Laidler, G.J., et al., Evaluating the Floe Edge Service: how well can SAR imagery address Inuit community concerns around sea ice change and travel safety? Canadian Geographer-Geographe Canadien, 2011. 55(1): p. 91-107.

27. Marino, E., The long history of environmental migration: Assessing vulnerability construction and obstacles to successful relocation in Shishmaref, Alaska. Global Environmental Change-Human and Policy Dimensions, 2012. 22(2): p. 374-381.

28. Marshall, N.A., Assessing Resource Dependency on the Rangelands as a Measure of Climate Sensitivity. Society & Natural Resources, 2011. 24(10): p. 1105-1115.

29. Mastrangelo, G., et al., Pattern and determinants of hospitalization during heat waves: an ecologic study. Bmc Public Health, 2007. 7.

30. Matulla, C., et al., Assessing the impact of a downscaled climate change simulation on the fish fauna in an Inner-Alpine River. International Journal of Biometeorology, 2007. 52(2): p. 127-137.

31. Mechler, R., et al., Modelling economic impacts and adaptation to extreme events: Insights from European case studies. Mitigation and Adaptation Strategies for Global Change, 2010. 15(7): p. 737-762.

32. Newton, A.C., Social-ecological Resilience and Biodiversity Conservation in a 900-year-old Protected Area. Ecology and Society, 2011. 16(4).

33. Orlandini, S., et al., Impacts of Climate Change and Variability on European Agriculture Results of Inventory Analysis in COST 734 Countries, in Trends and Directions in Climate Research, L. Gimeno, R. GarciaHerrera, and R.M. Trigo, Editors. 2008. p. 338-353.

34. Overbeck, M. and M. Schmidt, Modelling infestation risk of Norway spruce by Ips typographus (L.) in the Lower Saxon Harz Mountains (Germany). Forest Ecology and Management, 2012. 266: p. 115-125.

35. Prinn, R., et al., Scenarios with MIT integrated global systems model: significant global warming regardless of different approaches. Climatic Change, 2011. 104(3-4): p. 515-537.

36. Rongo, T., M. Bush, and R. van Woesik, Did ciguatera prompt the late Holocene Polynesian voyages of discovery? Journal of Biogeography, 2009. 36(8): p. 1423-1432.

37. Scally, J. and G. Wescott, Perceptions of Climate Change and Adaptation Responses in a Local Community: the Barwon Estuary Complex, Victoria. Australian Geographer, 2011. 42(4): p. 387-401.

38. Seidl, R., et al., An individual-based process model to simulate landscape-scale forest ecosystem dynamics. Ecological Modelling, 2012. 231: p. 87-100.

39. Selvaraju, R., R. Gommes, and M. Bernardi, Climate science in support of sustainable agriculture and food security. Climate Research, 2011. 47(1-2): p. 95-110.

40. Smith, C.L., et al., Fine-scale spatial temperature patterns across a UK conurbation. Climatic Change, 2011. 109(3-4): p. 269-286.

41. Sovacool, B.K., et al., Expert views of climate change adaptation in least developed Asia. Journal of Environmental Management, 2012. 97: p. 78-88.

42. Tschakert, P., et al., Holistic, adaptive management of the terrestrial carbon cycle at local and regional scales. Global Environmental Change-Human and Policy Dimensions, 2008. 18(1): p. 128-141.

43. Vogel, S., Leaves in the lowest and highest winds: temperature, force and shape. New Phytologist, 2009. 183(1): p. 13-26.

44. Wardekker, J.A., et al., Operationalising a resilience approach to adapting an urban delta to uncertain climate changes. Technological Forecasting and Social Change, 2010. 77(6): p. 987-998.

45. Warrick, R.A., Using SimCLIM for modelling the impacts of climate extremes in a changing climate: a preliminary case study of household water harvesting in Southeast Queensland. 18th World Imacs Congress and Modsim09 International Congress on Modelling and Simulation: Interfacing Modelling and Simulation with Mathematical and Computational Sciences, ed. R.S. Anderssen, R.D. Braddock, and L.T.H. Newham. 2009. 2583-2589.

46. Wood, R.G., Is there a Role for Cash Transfers in Climate Change Adaptation? Ids Bulletin-Institute of Development Studies, 2011. 42(6): p. 79-85.

**Natural Systems (n=295)**

1. Abad-Franch, F. and F.A. Monteiro, Biogeography and evolution of Amazonian triatomines (Heteroptera : Reduviidae): implications for Chagas disease surveillance in humid forest ecoregions. Memorias Do Instituto Oswaldo Cruz, 2007. 102: p. 57-69.

2. Abbott, I. and D. Le Maitre, Monitoring the impact of climate change on biodiversity: The challenge of megadiverse Mediterranean climate ecosystems. Austral Ecology, 2010. 35(4): p. 406-422.

3. Aberhan, M., W. Kiessling, and F.T. Fursich, Testing the role of biological interactions in the evolution of mid-Mesozoic marine benthic ecosystems. Paleobiology, 2006. 32(2): p. 259-277.

4. Abernethy, K.E., et al., Fuelling the decline in UK fishing communities? Ices Journal of Marine Science, 2010. 67(5): p. 1076-1085.

5. Allen, C.R., et al., Managing for resilience. Wildlife Biology, 2011. 17(4): p. 337-349.

6. Allen, C.R. and C.S. Holling, Novelty, Adaptive Capacity, and Resilience. Ecology and Society, 2010. 15(3).

7. Alvarez, J.C. and K. Vodden, Local Ecological Knowledge and the Impacts of Global Climatic Change on the Community of Seaweed Extractors in Pisco-Peru. Proceedings of Picmet 09 - Technology Management in the Age of Fundamental Change, Vols 1-5, ed. D.F. Kocaoglu, et al. 2009. 1015-1022.

8. Alvarez-Alvarez, P., et al., Relating growth and nutrition to site factors in young chestnut plantations established on agricultural and forest land in northern Spain. Agroforestry Systems, 2010. 79(3): p. 291-301.

9. Anderson, C.R. and S.M. McLachlan, Exiting, enduring and innovating: Farm household adaptation to global zoonotic disease. Global Environmental Change-Human and Policy Dimensions, 2012. 22(1): p. 82-93.

10. Angadi, S.V., et al., Adaptation of alternative pulse and oilseed crops to the semiarid Canadian Prairie: Seed yield and water use efficiency. Canadian Journal of Plant Science, 2008. 88(3): p. 425-438.

11. Angeler, D.G., C.R. Allen, and R.K. Johnson, Insight on Invasions and Resilience Derived from Spatiotemporal Discontinuities of Biomass at Local and Regional Scales. Ecology and Society, 2012. 17(2).

12. Angert, A.L., et al., Do species' traits predict recent shifts at expanding range edges? Ecology Letters, 2011. 14(7): p. 677-689.

13. Anilir, S., et al., Application of Infra-Free Motherboard (IFM) in a Decentralized Community for a Customized Real-time Processing Multidirectional Energy Supply Network. Journal of Asian Architecture and Building Engineering, 2009. 8(2): p. 407-414.

14. Attorre, F., et al., Evaluating the effects of climate change on tree species abundance and distribution in the Italian peninsula. Applied Vegetation Science, 2011. 14(2): p. 242-255.

15. Badjeck, M.-C., et al., Impacts of climate variability and change on fishery-based livelihoods. Marine Policy, 2010. 34(3): p. 375-383.

16. Badjeck, M.-C., et al., Climate variability and the Peruvian scallop fishery: the role of formal institutions in resilience building. Climatic Change, 2009. 94(1-2): p. 211-232.

17. Baker, A.C., P.W. Glynn, and B. Riegl, Climate change and coral reef bleaching: An ecological assessment of long-term impacts, recovery trends and future outlook. Estuarine Coastal and Shelf Science, 2008. 80(4): p. 435-471.

18. Balog, T., et al., The influence of season on oxidant-antioxidant status in trained and sedentary subjects. Life Sciences, 2006. 78(13): p. 1441-1447.

19. Ban, N.C., R.L. Pressey, and S. Weeks, Conservation Objectives and Sea-Surface Temperature Anomalies in the Great Barrier Reef. Conservation Biology, 2012. 26(5): p. 799-809.

20. Banks, S.C., et al., The effects of wildfire on mortality and resources for an arboreal marsupial: resilience to fire events but susceptibility to fire regime change. PloS one, 2011. 6(8): p. e22952-e22952.

21. Barbier, B., et al., Human Vulnerability to Climate Variability in the Sahel: Farmers' Adaptation Strategies in Northern Burkina Faso. Environmental Management, 2009. 43(5): p. 790-803.

22. Bardsen, B.-J., et al., Experimental evidence of a risk-sensitive reproductive allocation in a long-lived mammal. Ecology, 2008. 89(3): p. 829-837.

23. Barinova, S.S., G. Yehuda, and E. Nevo, Comparative analysis of algal communities in the rivers of northern and southern Israel as bearing on ecological consequences of climate change. Journal of Arid Environments, 2010. 74(7): p. 765-776.

24. Barton, B.T., Local adaptation to temperature conserves top-down control in a grassland food web. Proceedings of the Royal Society B-Biological Sciences, 2011. 278(1721): p. 3102-3107.

25. Baskett, M.L., S.D. Gaines, and R.M. Nisbet, Symbiont diversity may help coral reefs survive moderate climate change. Ecological Applications, 2009. 19(1): p. 3-17.

26. Beaumier, M.C. and J.D. Ford, Food Insecurity among Inuit Women Exacerbated by Socio-economic Stresses and Climate Change. Canadian Journal of Public Health-Revue Canadienne De Sante Publique, 2010. 101(3): p. 196-201.

27. Beeden, R., et al., A Framework for Responding to Coral Disease Outbreaks that Facilitates Adaptive Management. Environmental Management, 2012. 49(1): p. 1-13.

28. Bennema, S., et al., The use of bulk-tank milk ELISAs to assess the spatial distribution of Fasciola hepatica, Ostertagia ostertagi and Dictyocaulus viviparus in dairy cattle in Flanders (Belgium). Veterinary Parasitology, 2009. 165(1-2): p. 51-57.

29. Berard, A., et al., Severe drought-induced community tolerance to heat wave. An experimental study on soil microbial processes. Journal of Soils and Sediments, 2012. 12(4): p. 513-518.

30. Bhopal, R.S. and S.B. Rafnsson, Could mitochondrial efficiency explain the susceptibility to adiposity, metabolic syndrome, diabetes and cardiovascular diseases in South Asian populations? International Journal of Epidemiology, 2009. 38(4): p. 1072-1081.

31. Bijlsma, R. and V. Loeschcke, Genetic erosion impedes adaptive responses to stressful environments. Evolutionary Applications, 2012. 5(2): p. 117-129.

32. Binkley, D., et al., The role of old-growth forests in frequent-fire landscapes. Ecology and Society, 2007. 12(2).

33. Bjork, R.G. and U. Molau, Ecology of alpine snowbeds and the impact of global change. Arctic Antarctic and Alpine Research, 2007. 39(1): p. 34-43.

34. Bocquier, F. and E. Gonzalez-Garcia, Sustainability of ruminant agriculture in the new context: feeding strategies and features of animal adaptability into the necessary holistic approach. Animal, 2010. 4(7): p. 1258-1273.

35. Bonebrake, T.C. and C.A. Deutsch, Climate heterogeneity modulates impact of warming on tropical insects. Ecology, 2012. 93(3): p. 449-455.

36. Boon, H.J., et al., Bronfenbrenner's bioecological theory for modelling community resilience to natural disasters. Natural Hazards, 2012. 60(2): p. 381-408.

37. Boyles, J.G., B. Smit, and A.E. McKechnie, Variation in body temperature is related to ambient temperature but not experimental manipulation of insulation in two small endotherms with different thermoregulatory patterns. Journal of Zoology, 2012. 287(3): p. 224-232.

38. Bradford, J.B. and D.N. Kastendick, Age-related patterns of forest complexity and carbon storage in pine and aspen-birch ecosystems of northern Minnesota, USA. Canadian Journal of Forest Research-Revue Canadienne De Recherche Forestiere, 2010. 40(3): p. 401-409.

39. Brannlund, I. and P. Axelsson, Reindeer management during the colonization of Sami lands: A long-term perspective of vulnerability and adaptation strategies. Global Environmental Change-Human and Policy Dimensions, 2011. 21(3): p. 1095-1105.

40. Brodie, J.F. and E. Post, Nonlinear responses of wolverine populations to declining winter snowpack. Population Ecology, 2010. 52(2): p. 279-287.

41. Bucci, S.J., et al., Nutrient availability constrains the hydraulic architecture and water relations of savannah trees. Plant Cell and Environment, 2006. 29(12): p. 2153-2167.

42. Buckley, Y.M., The role of research for integrated management of invasive species, invaded landscapes and communities. Journal of Applied Ecology, 2008. 45(2): p. 397-402.

43. Buhay, J.E., Leading researchers to water: Assembling multi-disciplinary collective networks for the study of crustacean populations. Integrative and Comparative Biology, 2011. 51: p. E17-E17.

44. Bush, A., et al., Determining vulnerability of stream communities to climate change at the landscape scale. Freshwater Biology, 2012. 57(8): p. 1689-1701.

45. Buters, J.T.M., et al., Release of Bet v 1 from birch pollen from 5 European countries. Results from the HIALINE study. Atmospheric Environment, 2012. 55: p. 496-505.

46. Caffarra, A. and A. Donnelly, The ecological significance of phenology in four different tree species: effects of light and temperature on bud burst. International Journal of Biometeorology, 2011. 55(5): p. 711-721.

47. Callaway, J.C., et al., Emerging issues for the restoration of tidal marsh ecosystems in the context of predicted climate change. Madrono, 2007. 54(3): p. 234-248.

48. Camargo, C., et al., Community involvement in management for maintaining coral reef resilience and biodiversity in southern Caribbean marine protected areas. Biodiversity and Conservation, 2009. 18(4): p. 935-956.

49. Campoy, J.A., D. Ruiz, and J. Egea, Dormancy in temperate fruit trees in a global warming context: A review. Scientia Horticulturae, 2011. 130(2): p. 357-372.

50. Carvalho, S.B., et al., From climate change predictions to actions - conserving vulnerable animal groups in hotspots at a regional scale. Global Change Biology, 2010. 16(12): p. 3257-3270.

51. Cassinelli, J.D. and C.M. Moffitt, Comparison of Growth and Stress in Resident Redband Trout Held in Laboratory Simulations of Montane and Desert Summer Temperature Cycles. Transactions of the American Fisheries Society, 2010. 139(2): p. 339-352.

52. Cereghino, R., et al., Biological traits of European pond macroinvertebrates. Hydrobiologia, 2012. 689(1): p. 51-61.

53. Chapperon, C. and L. Seuront, Behavioral thermoregulation in a tropical gastropod: links to climate change scenarios. Global Change Biology, 2011. 17(4): p. 1740-1749.

54. Chytry, M., et al., Habitat invasions by alien plants: a quantitative comparison among Mediterranean, subcontinental and oceanic regions of Europe. Journal of Applied Ecology, 2008. 45(2): p. 448-458.

55. Cinner, J.E., et al., Gear-based fisheries management as a potential adaptive response to climate change and coral mortality. Journal of Applied Ecology, 2009. 46(3): p. 724-732.

56. Cirilo, A.G., et al., Morpho-physiological traits associated with maize crop adaptations to environments differing in nitrogen availability. Field Crops Research, 2009. 113(2): p. 116-124.

57. Clark, T.D., et al., Exceptional aerobic scope and cardiovascular performance of pink salmon (Oncorhynchus gorbuscha) may underlie resilience in a warming climate. Journal of Experimental Biology, 2011. 214(18): p. 3074-3081.

58. Colchero, F., et al., Predicting population survival under future climate change: density dependence, drought and extraction in an insular bighorn sheep. Journal of Animal Ecology, 2009. 78(3): p. 666-673.

59. Cooley, S.R., et al., Nutrition and income from molluscs today imply vulnerability to ocean acidification tomorrow. Fish and Fisheries, 2012. 13(2): p. 182-215.

60. Corlett, R.T., Impacts of warming on tropical lowland rainforests. Trends in Ecology & Evolution, 2011. 26(11): p. 606-613.

61. Cormont, A., et al., Using life-history traits to explain bird population responses to changing weather variability. Climate Research, 2011. 49(1): p. 59-U86.

62. Coulthard, S., Adapting to environmental change in artisanal fisheries - Insights from a South Indian Lagoon. Global Environmental Change-Human and Policy Dimensions, 2008. 18(3): p. 479-489.

63. Crimp, S.J., et al., Managing Murray-Darling Basin livestock systems in a variable and changing climate: challenges and opportunities. Rangeland Journal, 2010. 32(3): p. 293-304.

64. Crispo, E., et al., Broken barriers: Human-induced changes to gene flow and introgression in animals. Bioessays, 2011. 33(7): p. 508-518.

65. D'Amore, D.V., et al., Adaptation to exploit nitrate in surface soils predisposes yellow-cedar to climate-induced decline while enhancing the survival of western redcedar: A new hypothesis. Forest Ecology and Management, 2009. 258(10): p. 2261-2268.

66. Dahlhoff, E.P., et al., Effects of Temperature on Physiology and Reproductive Success of a Montane Leaf Beetle: Implications for Persistence of Native Populations Enduring Climate Change. Physiological and Biochemical Zoology, 2008. 81(6): p. 718-732.

67. Dale, A. and D. Armitage, Marine mammal co-management in Canada's Arctic: Knowledge co-production for learning and adaptive capacity. Marine Policy, 2011. 35(4): p. 440-449.

68. Dalgleish, H.J., D.N. Koons, and P.B. Adler, Can life-history traits predict the response of forb populations to changes in climate variability? Journal of Ecology, 2010. 98(1): p. 209-217.

69. Damiani, G., et al., Pro-inflammatory variants of DRB1 and RAGE genes are associated with susceptibility to pediatric type 1 diabetes: A new hypothesis on the adaptive role of Autoimmunity. Rivista Di Biologia-Biology Forum, 2007. 100(2): p. 285-304.

70. Daw, T.M., et al., To fish or not to fish: factors at multiple scales affecting artisanal fishers' readiness to exit a declining fishery. PloS one, 2012. 7(2): p. e31460-e31460.

71. de Witte, L.C., et al., AFLP markers reveal high clonal diversity and extreme longevity in four key arctic-alpine species. Molecular Ecology, 2012. 21(5): p. 1081-1097.

72. Dean, J.G. and H.J. Stain, Mental health impact for adolescents living with prolonged drought. Australian Journal of Rural Health, 2010. 18(1): p. 32-37.

73. del Mar Naval, M., et al., Analysis of genetic diversity among persimmon cultivars using microsatellite markers. Tree Genetics & Genomes, 2010. 6(5): p. 677-687.

74. Delmont, T.O., et al., Metagenomic mining for microbiologists. Isme Journal, 2011. 5(12): p. 1837-1843.

75. Denizot, M., J.W. Neal, and P. Gasque, Encephalitis due to emerging viruses: CNS innate immunity and potential therapeutic targets. Journal of Infection, 2012. 65(1): p. 1-16.

76. Denver, R.J., et al., Comparative endocrinology in the 21st century. Integrative and Comparative Biology, 2009. 49(4): p. 339-348.

77. DeSoto, L., et al., Geographically structured and temporally unstable growth responses of Juniperus thurifera to recent climate variability in the Iberian Peninsula. European Journal of Forest Research, 2012. 131(4): p. 905-917.

78. Donelson, J.M., et al., Rapid transgenerational acclimation of a tropical reef fish to climate change. Nature Climate Change, 2012. 2(1): p. 30-32.

79. Douhovnikoff, V. and R.S. Dodd, Lineage Divergence in Coast Redwood (Sequoia sempervirens), Detected by a New Set of Nuclear Microsatellite Loci. American Midland Naturalist, 2011. 165(1): p. 22-37.

80. Doyle, C.M., P.L. Leberg, and P.L. Klerks, Heritability of heat tolerance in a small livebearing fish, Heterandria formosa. Ecotoxicology, 2011. 20(3): p. 535-542.

81. Dubbin, W.E., M.G. Penn, and M.E. Hodson, Edaphic influences on plant community adaptation in the Chiquibul forest of Belize. Geoderma, 2006. 131(1-2): p. 76-88.

82. Dulin-Keita, A., et al., Do neighbourhoods matter? Neighbourhood disorder and long-term trends in serum cortisol levels. Journal of Epidemiology and Community Health, 2012. 66(1): p. 24-29.

83. Dupont, S., B. Lundve, and M. Thorndyke, Near Future Ocean Acidification Increases Growth Rate of the Lecithotrophic Larvae and Juveniles of the Sea Star Crossaster papposus. Journal of Experimental Zoology Part B-Molecular and Developmental Evolution, 2010. 314B(5): p. 382-389.

84. Ekue, M.R.M., et al., Population genetics of the cycad Encephalartos barteri ssp barteri (Zamiaceae) in Benin with notes on leaflet morphology and implications for conservation. Belgian Journal of Botany, 2008. 141(1): p. 78-94.

85. Elliott, J.M., The ecology of riffle beetles (Coleoptera: Elmidae). Freshwater Reviews, 2008. 1(2): p. 189-203.

86. Elliott, J.M. and J.A. Elliott, Temperature requirements of Atlantic salmon Salmo salar, brown trout Salmo trutta and Arctic charr Salvelinus alpinus: predicting the effects of climate change. Journal of Fish Biology, 2010. 77(8): p. 1793-1817.

87. Ellwood, M.D.F., et al., On the vapour trail of an atmospheric imprint in insects. Biology Letters, 2011. 7(4): p. 601-604.

88. Elmendorf, S.C., et al., Global assessment of experimental climate warming on tundra vegetation: heterogeneity over space and time. Ecology Letters, 2012. 15(2): p. 164-175.

89. Emma Huertas, I., et al., Warming will affect phytoplankton differently: evidence through a mechanistic approach. Proceedings of the Royal Society B-Biological Sciences, 2011. 278(1724): p. 3534-3543.

90. Evans, C.W., et al., Metabolic and behavioural adaptations during early development of the Antarctic silverfish, Pleuragramma antarcticum. Polar Biology, 2012. 35(6): p. 891-898.

91. Felizola Diniz-Filho, J.A., et al., Climate history, human impacts and global body size of Carnivora (Mammalia: Eutheria) at multiple evolutionary scales. Journal of Biogeography, 2009. 36(12): p. 2222-2236.

92. Felton, A., et al., Replacing coniferous monocultures with mixed-species production stands: An assessment of the potential benefits for forest biodiversity in northern Europe. Forest Ecology and Management, 2010. 260(6): p. 939-947.

93. Ferguson, S.H. and J.W. Higdon, How seals divide up the world: environment, life history, and conservation. Oecologia, 2006. 150(2): p. 318-329.

94. Ferrari, M.C.O., et al., Effects of Ocean Acidification on Learning in Coral Reef Fishes. PloS one, 2012. 7(2).

95. Fischer, D., et al., Combining Climatic Projections and Dispersal Ability: A Method for Estimating the Responses of Sandfly Vector Species to Climate Change. Plos Neglected Tropical Diseases, 2011. 5(11).

96. Foo, S.A., et al., Adaptive Capacity of the Habitat Modifying Sea Urchin Centrostephanus rodgersii to Ocean Warming and Ocean Acidification: Performance of Early Embryos. PloS one, 2012. 7(8).

97. Forrest, J. and J.D. Thomson, CONSEQUENCES OF VARIATION IN FLOWERING TIME WITHIN AND AMONG INDIVIDUALS OF MERTENSIA FUSIFORMIS (BORAGINACEAE), AN EARLY SPRING WILDFLOWER. American Journal of Botany, 2010. 97(1): p. 38-48.

98. Forrest, J.L., et al., Conservation and climate change: Assessing the vulnerability of snow leopard habitat to treeline shift in the Himalaya. Biological Conservation, 2012. 150(1): p. 129-135.

99. Forrest, J.R.K., et al., Seasonal change in a pollinator community and the maintenance of style length variation in Mertensia fusiformis (Boraginaceae). Annals of Botany, 2011. 108(1): p. 1-12.

100. Franssen, S.U., et al., Transcriptomic resilience to global warming in the seagrass Zostera marina, a marine foundation species. Proceedings of the National Academy of Sciences of the United States of America, 2011. 108(48): p. 19276-19281.

101. Franzen, M. and M. Molander, How threatened are alpine environments? a cross taxonomic study. Biodiversity and Conservation, 2012. 21(2): p. 517-526.

102. Franzen, M. and E. Ockinger, Climate-driven changes in pollinator assemblages during the last 60 years in an Arctic mountain region in Northern Scandinavia. Journal of Insect Conservation, 2012. 16(2): p. 227-238.

103. Frascaria-Lacoste, N. and J. Fernandez-Manjarres, Assisted Colonization of Foundation Species: Lack of Consideration of the Extended Phenotype Concept-Response to Kreyling et al. (2011). Restoration Ecology, 2012. 20(3): p. 296-298.

104. Frederiksen, M., et al., The demographic impact of extreme events: stochastic weather drives survival and population dynamics in a long-lived seabird. Journal of Animal Ecology, 2008. 77(5): p. 1020-1029.

105. Frick, W.F., et al., Climate and weather impact timing of emergence of bats. PloS one, 2012. 7(8): p. e42737-e42737.

106. Furberg, M., B. Evengard, and M. Nilsson, Facing the limit of resilience: perceptions of climate change among reindeer herding Sami in Sweden. Global health action, 2011. 4.

107. Galvin, K.A., Transitions: Pastoralists Living with Change, in Annual Review of Anthropology. 2009. p. 185-198.

108. Garcia-Robledo, E., et al., Photosynthetic activity and community shifts of microphytobenthos covered by green macroalgae. Environmental Microbiology Reports, 2012. 4(3): p. 316-325.

109. Gargouri, K., H. Bentaher, and A. Rhouma, A novel method to assess drought stress of olive tree. Agronomy for Sustainable Development, 2012. 32(3): p. 735-745.

110. Garraud, S. and L. Mahamane, Evolution of agropastoral community adaptation practices in the regions of North Tillabery and Tahoua in Niger against the background of climate change. Secheresse (Montrouge), 2012. 23(1): p. 24-30.

111. Garrity, D.P., et al., Evergreen Agriculture: a robust approach to sustainable food security in Africa. Food Security, 2010. 2(3): p. 197-214.

112. Gaudart, J., et al., Modelling malaria incidence with environmental dependency in a locality of Sudanese savannah area, Mali. Malaria Journal, 2009. 8.

113. Gaudes, A., J. Artigas, and I. Munoz, Species traits and resilience of meiofauna to floods and drought in a Mediterranean stream. Marine and Freshwater Research, 2010. 61(11): p. 1336-1347.

114. Gebrekirstos, A., et al., Relationships of stable carbon isotopes, plant water potential and growth: an approach to asses water use efficiency and growth strategies of dry land agroforestry species. Trees-Structure and Function, 2011. 25(1): p. 95-102.

115. Geneau, R., et al., Chronic Diseases: Chronic Diseases and Development 1 Raising the priority of preventing chronic diseases: a political process. Lancet, 2010. 376(9753): p. 1689-1698.

116. Gessler, A., et al., Potential risks for European beech (Fagus sylvatica L.) in a changing climate. Trees-Structure and Function, 2007. 21(1): p. 1-11.

117. Gilgen, A.K. and N. Buchmann, Response of temperate grasslands at different altitudes to simulated summer drought differed but scaled with annual precipitation. Biogeosciences, 2009. 6(11): p. 2525-2539.

118. Gillespie, R.G., E.M. Claridge, and G.K. Roderick, Biodiversity dynamics in isolated island communities: interaction between natural and human-mediated processes. Molecular Ecology, 2008. 17(1): p. 45-57.

119. Gilman, R.T., et al., Evolution of plant-pollinator mutualisms in response to climate change. Evolutionary Applications, 2012. 5(1): p. 2-16.

120. Girard, C. and S. Renaud, Disparity Changes in 370 Ma Devonian Fossils: The Signature of Ecological Dynamics? PloS one, 2012. 7(4).

121. Godfrey, L.R. and M.T. Irwin, The evolution of extinction risk: Past and present anthropogenic impacts on the primate communities of Madagascar. Folia Primatologica, 2007. 78(5-6): p. 405-419.

122. Gonzalez-Benecke, C.A. and T.A. Martin, Water availability and genetic effects on water relations of loblolly pine (Pinus taeda) stands. Tree Physiology, 2010. 30(3): p. 376-392.

123. Goosey, E. and S. Harrad, Perfluoroalkyl substances in UK indoor and outdoor air: Spatial and seasonal variation, and implications for human exposure. Environment International, 2012. 45: p. 86-90.

124. Gowland, R.L. and A.G. Western, Morbidity in the marshes: Using spatial epidemiology to investigate skeletal evidence for malaria in Anglo-Saxon England (AD 410-1050). American Journal of Physical Anthropology, 2012. 147(2): p. 301-311.

125. Grasso, M. and G. Feola, Mediterranean agriculture under climate change: adaptive capacity, adaptation, and ethics. Regional Environmental Change, 2012. 12(3): p. 607-618.

126. Gurbuz, B., et al., Adaptation of endemic mediterranean Sternbergia candida Mathew Et T. Baytop in the continental climate of central anatolia. Scientia Horticulturae, 2009. 123(1): p. 99-103.

127. Healey, M., The cumulative impacts of climate change on Fraser River sockeye salmon (Oncorhynchus nerka) and implications for management (vol 68, pg 718, 2011). Canadian Journal of Fisheries and Aquatic Sciences, 2011. 68(5): p. 953-953.

128. Heino, J., R. Virkkala, and H. Toivonen, Climate change and freshwater biodiversity: detected patterns, future trends and adaptations in northern regions. Biological Reviews, 2009. 84(1): p. 39-54.

129. Henaux, V., et al., Establishing Winter Origins of Migrating Lesser Snow Geese Using Stable Isotopes. Avian Conservation and Ecology, 2012. 7(1).

130. Hennige, S.J., et al., Acclimation and adaptation of scleractinian coral communities along environmental gradients within an Indonesian reef system. Journal of Experimental Marine Biology and Ecology, 2010. 391(1-2): p. 143-152.

131. Hernandez-Martinez, J., et al., HPinus greggii var. Australis seed zones in Hidalgo, Mexico. Revista Fitotecnia Mexicana, 2007. 30(3): p. 241-249.

132. Hewitt, N., et al., Taking stock of the assisted migration debate. Biological Conservation, 2011. 144(11): p. 2560-2572.

133. Hitchings, T.R., Leptophlebiidae (Ephemeroptera) of the alpine region of the Southern Alps, New Zealand. Aquatic Insects, 2009. 31: p. 595-601.

134. Hoffmann, I., Climate change and the characterization, breeding and conservation of animal genetic resources. Animal Genetics, 2010. 41: p. 32-46.

135. Hofmann, G.E., et al., The Effect of Ocean Acidification on Calcifying Organisms in Marine Ecosystems: An Organism-to-Ecosystem Perspective, in Annual Review of Ecology, Evolution, and Systematics, Vol 41, D.J. Futuyma, H.B. Shafer, and D. Simberloff, Editors. 2010. p. 127-147.

136. Hogerwerf, L., et al., Persistence of Highly Pathogenic Avian Influenza H5N1 Virus Defined by Agro-Ecological Niche. Ecohealth, 2010. 7(2): p. 213-225.

137. Hoye, T.T. and M.C. Forchhammer, Phenology of high-arctic arthropods: Effects of climate on spatial, seasonal, and inter-annual variation. Advances in Ecological Research, Vol 40, 2008. 40: p. 299-324.

138. Huang, T.C., Y.T. Hsu, and Y.C. Chou, Influence of climate change on the incidence of rice diseases and our adaptive strategies. Plant Protection Bulletin (Taichung), 2010. 52(2): p. 25-42.

139. Hubert, P., et al., Ecological factors driving the higher hedgehog (Erinaceus europeaus) density in an urban area compared to the adjacent rural area. Landscape and Urban Planning, 2011. 103(1): p. 34-43.

140. Huey, R.B., et al., Predicting organismal vulnerability to climate warming: roles of behaviour, physiology and adaptation. Philosophical Transactions of the Royal Society B-Biological Sciences, 2012. 367(1596): p. 1665-1679.

141. Hughes, T.P., et al., Shifting base-lines, declining coral cover, and the erosion of reef resilience: comment on Sweatman et al. (2011). Coral Reefs, 2011. 30(3): p. 653-660.

142. Jaenicke, H., et al., Impacts of Underutilized Plant Species Promotion on Biodiversity, in International Symposium on Underutilized Plants for Food Security, Nutrition, Income and Sustainable Development, H. Jaenicke, et al., Editors. 2009. p. 621-627.

143. Jagadish, S.V.K., et al., Genetic Advances in Adapting Rice to a Rapidly Changing Climate. Journal of Agronomy and Crop Science, 2012. 198(5): p. 360-373.

144. Jat, H.S., R.K. Singh, and J.S. Mann, Ardu (Ailanthus sp) in arid ecosystem: A compatible species for combating with drought and securing livelihood security of resource poor people. Indian Journal of Traditional Knowledge, 2011. 10(1): p. 102-113.

145. Jelenik, Z., et al., Tick-borne encephalitis and golden agers: position paper of the International Scientific Working Group on Tick-borne encephalitis (ISW-TBE). Wiener medizinische Wochenschrift (1946), 2010. 160(9-10): p. 247-51.

146. Jennings, T.L., Transcending the Adaptation/Mitigation Climate Change Science Policy Debate: Unmasking Assumptions about Adaptation and Resilience. Weather Climate and Society, 2011. 3(4): p. 238-248.

147. Jiguet, F., et al., Climate envelope, life history traits and the resilience of birds facing global change. Global Change Biology, 2007. 13(8): p. 1672-1684.

148. Johnson, S.E., et al., Gray-headed Lemur (Eulemur cinereiceps) Abundance and Forest Structure Dynamics at Manombo, Madagascar. Biotropica, 2011. 43(3): p. 371-379.

149. Johst, K., et al., Biodiversity conservation in dynamic landscapes: trade-offs between number, connectivity and turnover of habitat patches. Journal of Applied Ecology, 2011. 48(5): p. 1227-1235.

150. Junk, W.J. and P.B. Bayley, The scope of the flood pulse concept regarding riverine fish and fisheries, given geographic and man-made differences among systems, in Reconciling Fisheries with Conservation, Vols I and Ii, J. Nielsen, et al., Editors. 2008. p. 1907-1923.

151. Junk, W.J., et al., Biodiversity and its conservation in the Pantanal of Mato Grosso, Brazil. Aquatic Sciences, 2006. 68(3): p. 278-309.

152. Kattwinkel, M., et al., Climate change, agricultural insecticide exposure, and risk for freshwater communities. Ecological Applications, 2011. 21(6): p. 2068-2081.

153. Kaukoranta, T., R. Tahvonen, and A. Ylamaki, Climatic potential and risks for apple growing by 2040. Agricultural and Food Science, 2010. 19(2): p. 144-159.

154. Kausrud, K.L., et al., Trees Wanted-Dead or Alive! Host Selection and Population Dynamics in Tree-Killing Bark Beetles. PloS one, 2011. 6(5).

155. Kausrud, K.L., et al., Trees wanted--dead or alive! Host selection and population dynamics in tree-killing bark beetles. PloS one, 2011. 6(5): p. e18274-e18274.

156. Keatinge, J.D.H., et al., Overcoming chronic malnutrition in a future warming world: the key importance of mungbean and vegetable soybean. Euphytica, 2011. 180(1): p. 129-141.

157. Keith, S.A., et al., Individualistic species limitations of climate-induced range expansions generated by meso-scale dispersal barriers. Diversity and Distributions, 2011. 17(2): p. 275-286.

158. Kellermann, V., et al., Upper thermal limits of Drosophila are linked to species distributions and strongly constrained phylogenetically. Proceedings of the National Academy of Sciences of the United States of America, 2012. 109(40): p. 16228-33.

159. Kelman, I., Introduction to "Municipalities addressing climate change: a case study of Norway". International Journal of Climate Change Strategies and Management, 2011. 3(4): p. 338-344.

160. Khrunin, A., et al., Regional differences in the genetic variability of Finno-Ugric speaking Komi populations. American Journal of Human Biology, 2007. 19(6): p. 741-750.

161. Kipfer, T., et al., Ectomycorrhiza succession patterns in Pinus sylvestris forests after stand-replacing fire in the Central Alps. Oecologia, 2011. 167(1): p. 219-228.

162. Kirchhof, S., et al., The reptile species assemblage of the Soutpansberg (Limpopo Province, South Africa) and its characteristics. Salamandra, 2010. 46(3): p. 147-166.

163. Kleinteich, J., et al., Temperature-related changes in polar cyanobacterial mat diversity and toxin production. Nature Climate Change, 2012. 2(5): p. 356-360.

164. Knudsen, E., et al., Challenging claims in the study of migratory birds and climate change. Biological Reviews, 2011. 86(4): p. 928-946.

165. Kofinas, G.P., et al., Resilience of Athabascan subsistence systems to interior Alaska's changing climate. Canadian Journal of Forest Research-Revue Canadienne De Recherche Forestiere, 2010. 40(7): p. 1347-1359.

166. Kokko, H. and A. Lopez-Sepulcre, From individual dispersal to species ranges: Perspectives for a changing world. Science, 2006. 313(5788): p. 789-791.

167. Kordas, R.L., C.D.G. Harley, and M.I. O'Connor, Community ecology in a warming world: The influence of temperature on interspecific interactions in marine systems. Journal of Experimental Marine Biology and Ecology, 2011. 400(1-2): p. 218-226.

168. Korunka, C., et al., Predictors of a successful implementation of an ergonomic training program. Applied Ergonomics, 2010. 42(1): p. 98-105.

169. Kriticos, D.J., Regional climate-matching to estimate current and future sources of biosecurity threats. Biological Invasions, 2012. 14(8): p. 1533-1544.

170. Kubisch, A. and H.-J. Poethke, Range border formation in a world with increasing climatic variance. Evolutionary Ecology Research, 2011. 13(2): p. 159-169.

171. Kueffer, C., Alien Plants in Mountains-State of Knowledge and Management Needs. Gesunde Pflanzen, 2011. 63(2): p. 63-68.

172. Kumar, S., et al., Genetic improvement of grass pea for low neurotoxin (beta-ODAP) content. Food and Chemical Toxicology, 2011. 49(3): p. 589-600.

173. Kurihara, H., Effects of CO2-driven ocean acidification on the early developmental stages of invertebrates. Marine Ecology-Progress Series, 2008. 373: p. 275-284.

174. La Rovere, E.L., A.C. Avzaradel, and J.M. Guimaraes Monteiro, Potential synergy between adaptation and mitigation strategies: production of vegetable oils and biodiesel in northeastern Brazil. Climate Research, 2009. 40(2-3): p. 233-239.

175. Lau, C.L., et al., Climate change, flooding, urbanisation and leptospirosis: fuelling the fire? Transactions of the Royal Society of Tropical Medicine and Hygiene, 2010. 104(10): p. 631-638.

176. Lawler, J.J. and J.D. Olden, Reframing the debate over assisted colonization. Frontiers in Ecology and the Environment, 2011. 9(10): p. 569-574.

177. Lawson, C.R., et al., Local and landscape management of an expanding range margin under climate change. Journal of Applied Ecology, 2012. 49(3): p. 552-561.

178. Lebl, K., et al., Survival rates in a small hibernator, the edible dormouse: a comparison across Europe. Ecography, 2011. 34(4): p. 683-692.

179. Leimu, R., et al., Habitat fragmentation, climate change, and inbreeding in plants, in Year in Ecology and Conservation Biology 2010, R.S. Ostfeld and W.H. Schlesinger, Editors. 2010. p. 84-98.

180. Lepetz, V., et al., Biodiversity monitoring: some proposals to adequately study species' responses to climate change. Biodiversity and Conservation, 2009. 18(12): p. 3185-3203.

181. Li, J., et al., Farmers' adoption of maize (Zea mays L.) hybrids and the persistence of landraces in Southwest China: implications for policy and breeding. Genetic Resources and Crop Evolution, 2012. 59(6): p. 1147-1160.

182. Li, W. and Y. Li, Managing Rangeland as a Complex System: How Government Interventions Decouple Social Systems from Ecological Systems. Ecology and Society, 2012. 17(1).

183. Liao, W.B., C.Q. Zhou, and J.C. Hu, Head-body length variation in the mole-shrew (Anourosorex squamipes) in relation to annual temperature and elevation. North-Western Journal of Zoology, 2011. 7(1): p. 47-54.

184. Longo S, M., et al., Macroinvertebrate community dynamics in the Potrerillos stream (Colombia): Response to seasonal flow changes. Limnetica, 2010. 29(2): p. 195-210.

185. Luna-Vega, I., et al., Ecological niche modeling on the effect of climatic change and conservation of Ternstroemia lineata DC. (Ternstroemiaceae) in Mesoamerica. Botany-Botanique, 2012. 90(7): p. 637-650.

186. Maazouzi, C., et al., Ecophysiological responses to temperature of the "killer shrimp" Dikerogammarus villosus: Is the invader really stronger than the native Gammarus pulex? Comparative Biochemistry and Physiology a-Molecular & Integrative Physiology, 2011. 159(3): p. 268-274.

187. Mable, B.K., M.A. Alexandrou, and M.I. Taylor, Genome duplication in amphibians and fish: an extended synthesis. Journal of Zoology, 2011. 284(3): p. 151-182.

188. MacNeil, M.A., et al., Transitional states in marine fisheries: adapting to predicted global change. Philosophical Transactions of the Royal Society B-Biological Sciences, 2010. 365(1558): p. 3753-3763.

189. Markesteijn, L. and L. Poorter, Seedling root morphology and biomass allocation of 62 tropical tree species in relation to drought- and shade-tolerance. Journal of Ecology, 2009. 97(2): p. 311-325.

190. Massot, M., J. Clobert, and R. Ferriere, Climate warming, dispersal inhibition and extinction risk. Global Change Biology, 2008. 14(3): p. 461-469.

191. Mazzocchi, M.G., et al., Stability and resilience in coastal copepod assemblages: The case of the Mediterranean long-term ecological research at Station MC (LTER-MC). Progress in Oceanography, 2012. 97: p. 135-151.

192. McDonald-Madden, E., et al., Optimal timing for managed relocation of species faced with climate change. Nature Climate Change, 2011. 1(5): p. 261-265.

193. McDougall, K.L., et al., Alien flora of mountains: global comparisons for the development of local preventive measures against plant invasions. Diversity and Distributions, 2011. 17(1): p. 103-111.

194. McIntyre, S., Ecological and anthropomorphic factors permitting low-risk assisted colonization in temperate grassy woodlands. Biological Conservation, 2011. 144(6): p. 1781-1789.

195. McLeman, R.A. and S.K. Ploeger, Soil and its influence on rural drought migration: insights from Depression-era Southwestern Saskatchewan, Canada. Population and Environment, 2012. 33(4): p. 304-332.

196. Medeiros, J.S., et al., Variation in seedling freezing response is associated with climate in Larrea. Oecologia, 2012. 169(1): p. 73-84.

197. Meek, C.L., et al., Adaptive governance and the human dimensions of marine mammal management: Implications for policy in a changing North. Marine Policy, 2011. 35(4): p. 466-476.

198. Mehner, T., Diel vertical migration of freshwater fishes - proximate triggers, ultimate causes and research perspectives. Freshwater Biology, 2012. 57(7): p. 1342-1359.

199. Mellone, U., et al., Weather conditions promote route flexibility during open ocean crossing in a long-distance migratory raptor. International Journal of Biometeorology, 2011. 55(4): p. 463-468.

200. Menu, F., et al., Adaptive Developmental Delay in Chagas Disease Vectors: An Evolutionary Ecology Approach. Plos Neglected Tropical Diseases, 2010. 4(5).

201. Mitchell, N.J., et al., Demographic effects of temperature-dependent sex determination: will tuatara survive global warming? Global Change Biology, 2010. 16(1): p. 60-72.

202. Moller, A.P., Basal metabolic rate and risk-taking behaviour in birds. Journal of Evolutionary Biology, 2009. 22(12): p. 2420-2429.

203. Monaghan, K.A. and A.M.V.M. Soares, The bioassessment of fish and macroinvertebrates in a Mediterranean-Atlantic climate: Habitat assessment and concordance between contrasting ecological samples. Ecological Indicators, 2010. 10(2): p. 184-191.

204. Monteiro-Henriques, T. and M. Dalila Espirito-Santo, Climate change and the outdoor regional living plant collections: an example from mainland Portugal. Biodiversity and Conservation, 2011. 20(2): p. 335-343.

205. Montoya, J.M. and D. Raffaelli, Climate change, biotic interactions and ecosystem services. Philosophical Transactions of the Royal Society B-Biological Sciences, 2010. 365(1549): p. 2013-2018.

206. Mooney, H., et al., Biodiversity, climate change, and ecosystem services. Current Opinion in Environmental Sustainability, 2009. 1(1): p. 46-54.

207. Moore, J.W. and D.E. Schindler, Spawning salmon and the phenology of emergence in stream insects. Proceedings of the Royal Society B-Biological Sciences, 2010. 277(1688): p. 1695-1703.

208. Morand, S. and J.F. Guegan, How the biodiversity sciences may aid biological tools and ecological engineering to assess the impact of climatic changes. Revue Scientifique Et Technique-Office International Des Epizooties, 2008. 27(2): p. 355-366.

209. Moretti, M., et al., Fire-induced taxonomic and functional changes in saproxylic beetle communities in fire sensitive regions. Ecography, 2010. 33(4): p. 760-771.

210. Morimoto, Y., Biodiversity and ecosystem services in urban areas for smart adaptation to climate change: "Do you Kyoto"? Landscape and Ecological Engineering, 2011. 7(1): p. 9-16.

211. Mormede, P., et al., Breeding for robustness: the role of cortisol. Animal, 2011. 5(5): p. 651-657.

212. Morris, M.J., E.S. Na, and A.K. Johnson, Salt craving: The psychobiology of pathogenic sodium intake. Physiology & Behavior, 2008. 94(5): p. 709-721.

213. Myers, W.L., et al., Contextual clustering for configuring collaborative conservation of watersheds in the Mid-Atlantic Highlands. Environmental and Ecological Statistics, 2006. 13(4): p. 391-407.

214. Nardone, A., et al., Effects of climate changes on animal production and sustainability of livestock systems. Livestock Science, 2010. 130(1-3): p. 57-69.

215. Newton, A.C., et al., Climate Change and Defense against Pathogens in Plants. Advances in applied microbiology, 2012. 81: p. 89-132.

216. Nitschke, C.R. and J.L. Innes, A tree and climate assessment tool for modelling ecosystem response to climate change. Ecological Modelling, 2008. 210(3): p. 263-277.

217. Noroozi, J., et al., The subnival-nival vascular plant species of Iran: a unique high-mountain flora and its threat from climate warming. Biodiversity and Conservation, 2011. 20(6): p. 1319-1338.

218. Nunes, F., R.D. Norris, and N. Knowlton, Implications of isolation and low genetic diversity in peripheral populations of an amphi-Atlantic coral. Molecular Ecology, 2009. 18(20): p. 4283-4297.

219. O'Neill, S.J. and J. Handmer, Responding to bushfire risk: the need for transformative adaptation. Environmental Research Letters, 2012. 7(1).

220. Ogden, A.E. and J.L. Innes, Application of Structured Decision Making to an Assessment of Climate Change Vulnerabilities and Adaptation Options for Sustainable Forest Management. Ecology and Society, 2009. 14(1).

221. Olden, J.D., et al., Challenges and Opportunities in Implementing Managed Relocation for Conservation of Freshwater Species. Conservation Biology, 2011. 25(1): p. 40-47.

222. Olden, J.D., N.L. Poff, and K.R. Bestgen, Trait synergisms and the rarity, extirpation, and extinction risk of desert fishes. Ecology, 2008. 89(3): p. 847-856.

223. Ooi, M.K.J., Seed bank persistence and climate change. Seed Science Research, 2012. 22: p. S53-S60.

224. Orsini, L., et al., Single nucleotide polymorphism discovery from expressed sequence tags in the waterflea Daphnia magna. Bmc Genomics, 2011. 12.

225. Ortiz-Milan, S.M., Project of recovery the biological conditions of the production system in saltworks of Industria Salinera de Yucatan SA de C.V. (ISYSA) damaged by the hurricane Isidore in September of 2002. Proceedings of the 1st International Conference on the Ecological Importance of Solar Saltworks, ed. T.D. Lekkas and N.A. Korovessis. 2006. 25-30.

226. Oswald, S.A. and J.M. Arnold, Direct impacts of climatic warming on heat stress in endothermic species: seabirds as bioindicators of changing thermoregulatory constraints. Integrative Zoology, 2012. 7(2): p. 121-136.

227. Oswald, S.A., et al., Heat stress in a high-latitude seabird: effects of temperature and food supply on bathing and nest attendance of great skuas Catharacta skua. Journal of Avian Biology, 2008. 39(2): p. 163-169.

228. Park, A. and C. Talbot, Assisted migration: uncertainty, risk and opportunity. Forestry Chronicle, 2012. 88(4): p. 412-419.

229. Parkins, J.R. and N.A. MacKendrick, Assessing community vulnerability: A study of the mountain pine beetle outbreak in British Columbia, Canada. Global Environmental Change-Human and Policy Dimensions, 2007. 17(3-4): p. 460-471.

230. Pearce-Higgins, J.W., Using diet to assess the sensitivity of northern and upland birds to climate change. Climate Research, 2010. 45(1): p. 119-U435.

231. Pearson, G.A., A. Lago-Leston, and C. Mota, Frayed at the edges: selective pressure and adaptive response to abiotic stressors are mismatched in low diversity edge populations. Journal of Ecology, 2009. 97(3): p. 450-462.

232. Peiser, B., et al., Seasonal changes and physiological responses: Their impact on activity, health, exercise and athletic performance. International Sportmed Journal, 2006. 7(1): p. 16-32.

233. Pichancourt, J.-B. and R.D. van Klinken, Phenotypic plasticity influences the size, shape and dynamics of the geographic distribution of an invasive plant. PloS one, 2012. 7(2): p. e32323-e32323.

234. Pilling, D., et al., Sustaining livestock biodiversity - from assessment to action. Biodiversity (Ottawa), 2008. 9(1-2): p. 14-18.

235. Pitt, J.P.W., J. Regniere, and S. Worner, Risk assessment of the gypsy moth, Lymantria dispar (L), in New Zealand based on phenology modelling. International Journal of Biometeorology, 2007. 51(4): p. 295-305.

236. Planque, B., et al., How does fishing alter marine populations and ecosystems sensitivity to climate? Journal of Marine Systems, 2010. 79(3-4): p. 403-417.

237. Polato, N.R., et al., Location-Specific Responses to Thermal Stress in Larvae of the Reef-Building Coral Montastraea faveolata. PloS one, 2010. 5(6).

238. Prober, S.M., et al., Combining community-level spatial modelling and expert knowledge to inform climate adaptation in temperate grassy eucalypt woodlands and related grasslands. Biodiversity and Conservation, 2012. 21(7): p. 1627-1650.

239. Prober, S.M. and F.P. Smith, Enhancing biodiversity persistence in intensively used agricultural landscapes: A synthesis of 30 years of research in the Western Australian wheatbelt. Agriculture Ecosystems & Environment, 2009. 132(3-4): p. 173-191.

240. Procaccini, G., J.L. Olsen, and T.B.H. Reusch, Contribution of genetics and genomics to seagrass biology and conservation. Journal of Experimental Marine Biology and Ecology, 2007. 350(1-2): p. 234-259.

241. Reed, T.E., et al., Time to evolve? Potential evolutionary responses of fraser river sockeye salmon to climate change and effects on persistence. PloS one, 2011. 6(6): p. e20380-e20380.

242. Reed, T.E., D.E. Schindler, and R.S. Waples, Interacting Effects of Phenotypic Plasticity and Evolution on Population Persistence in a Changing Climate. Conservation Biology, 2011. 25(1): p. 56-63.

243. Regan, H.M., et al., Evaluation of assisted colonization strategies under global change for a rare, fire-dependent plant. Global Change Biology, 2012. 18(3): p. 936-947.

244. Reif, A., et al., Forest Management in Times of Climate Change - Synergies and potential conflicts between forestry and nature conservation. Naturschutz und Landschaftsplanung, 2010. 42(9): p. 261-266.

245. Ribas, L., et al., Expression Profiling the Temperature-Dependent Amphibian Response to Infection by Batrachochytrium dendrobatidis. PloS one, 2009. 4(12).

246. Roulin, A., R. Burri, and S. Antoniazza, Owl melanin-based plumage redness is more frequent near than away from the equator: implications on the effect of climate change on biodiversity. Biological Journal of the Linnean Society, 2011. 102(3): p. 573-582.

247. Rubenstein, D.R., Spatiotemporal environmental variation, risk aversion, and the evolution of cooperative breeding as a bet-hedging strategy. Proceedings of the National Academy of Sciences of the United States of America, 2011. 108: p. 10816-10822.

248. Ruelle, M.L. and K.-A.S. Kassam, Diversity of Plant Knowledge as an Adaptive Asset: A Case Study with Standing Rock Elders. Economic Botany, 2011. 65(3): p. 295-307.

249. Salinas, S. and S.B. Munch, Thermal legacies: transgenerational effects of temperature on growth in a vertebrate. Ecology Letters, 2012. 15(2): p. 159-163.

250. Sanchez-Salguero, R., et al., Selective drought-induced decline of pine species in southeastern Spain. Climatic Change, 2012. 113(3-4): p. 767-785.

251. Sardella, B.A., E. Sanmarti, and D. Kultz, The acute temperature tolerance of green sturgeon (Acipenser medirostris) and the effect of environmental salinity. Journal of Experimental Zoology Part a-Ecological Genetics and Physiology, 2008. 309A(8): p. 477-483.

252. Saxon, E., Noah's Parks: A partial antidote to the Anthropocene extinction event. Biodiversity (Ottawa), 2008. 9(3-4): p. 5-10.

253. Sayles, J.S. and M.E. Mulrennan, Securing a Future: Cree Hunters' Resistance and Flexibility to Environmental Changes, Wemindji, James Bay. Ecology and Society, 2010. 15(4).

254. Scharf, I., et al., Impact of a social parasite on ant host populations depends on host species, habitat and year. Biological Journal of the Linnean Society, 2011. 103(3): p. 559-570.

255. Schwandt, J.W., et al., Current health issues and management strategies for white pines in the western United States and Canada. Forest Pathology, 2010. 40(3-4): p. 226-250.

256. Sekercioglu, C.H., R.B. Primack, and J. Wormworth, The effects of climate change on tropical birds. Biological Conservation, 2012. 148(1): p. 1-18.

257. Shackleton, S.E. and C.M. Shackleton, Linking poverty, HIV/AIDS and climate change to human and ecosystem vulnerability in southern Africa: consequences for livelihoods and sustainable ecosystem management. International Journal of Sustainable Development and World Ecology, 2012. 19(3): p. 275-286.

258. Sherman, J.A., Evolutionary origin of bipolar disorder-revised: EOBD-R. Medical Hypotheses, 2012. 78(1): p. 113-122.

259. Shimode, S., et al., Life history and ontogenetic vertical migration of Neocalanus gracilis in the western North Pacific Ocean. Aquatic Biology, 2009. 7(3): p. 295-306.

260. Shimono, H., Earlier rice phenology as a result of climate change can increase the risk of cold damage during reproductive growth in northern Japan. Agriculture Ecosystems & Environment, 2011. 144(1): p. 201-207.

261. Shoo, L.P., et al., Engineering a future for amphibians under climate change. Journal of Applied Ecology, 2011. 48(2): p. 487-492.

262. Sih, A., M.C.O. Ferrari, and D.J. Harris, Evolution and behavioural responses to human-induced rapid environmental change. Evolutionary Applications, 2011. 4(2): p. 367-387.

263. Simberloff, D., The Role of Propagule Pressure in Biological Invasions, in Annual Review of Ecology Evolution and Systematics. 2009. p. 81-102.

264. Sperry, J.H. and P.J. Weatherhead, PREY-MEDIATED EFFECTS OF DROUGHT ON CONDITION AND SURVIVAL OF A TERRESTRIAL SNAKE. Ecology, 2008. 89(10): p. 2770-2776.

265. Stien, A., et al., Icing events trigger range displacement in a high-arctic ungulate. Ecology, 2010. 91(3): p. 915-920.

266. Stohlgren, T.J., et al., Ensemble Habitat Mapping of Invasive Plant Species. Risk Analysis, 2010. 30(2): p. 224-235.

267. Stork, N.E., et al., Vulnerability and Resilience of Tropical Forest Species to Land-Use Change. Conservation Biology, 2009. 23(6): p. 1438-1447.

268. Summers, D.M., et al., Species vulnerability to climate change: impacts on spatial conservation priorities and species representation. Global Change Biology, 2012. 18(7): p. 2335-2348.

269. Sundar, K.S.G., Agricultural intensification, rainfall patterns, and large waterbird breeding success in the extensively cultivated landscape of Uttar Pradesh, India. Biological Conservation, 2011. 144(12): p. 3055-3063.

270. Sutherland, W.J., Predicting the ecological consequences of environmental change: a review of the methods. Journal of Applied Ecology, 2006. 43(4): p. 599-616.

271. Thomas, C.D., et al., Protected areas facilitate species' range expansions. Proceedings of the National Academy of Sciences of the United States of America, 2012. 109(35): p. 14063-14068.

272. Traill, L.W., et al., Mechanisms driving change: altered species interactions and ecosystem function through global warming. Journal of Animal Ecology, 2010. 79(5): p. 937-947.

273. Tscharntke, T., et al., Multifunctional shade-tree management in tropical agroforestry landscapes - a review. Journal of Applied Ecology, 2011. 48(3): p. 619-629.

274. Tuck, G., et al., The potential distribution of bioenergy crops in Europe under present and future climate. Biomass & Bioenergy, 2006. 30(3): p. 183-197.

275. Tyack, P.L., Implications for marine mammals of large-scale changes in the marine acoustic environment. Journal of Mammalogy, 2008. 89(3): p. 549-558.

276. Varpe, O., Fitness and phenology: annual routines and zooplankton adaptations to seasonal cycles. Journal of Plankton Research, 2012. 34(4): p. 267-276.

277. Verboven, N., et al., Adrenocortical function of Arctic-breeding glaucous gulls in relation to persistent organic pollutants. General and Comparative Endocrinology, 2010. 166(1): p. 25-32.

278. Vigotti, M.A., V.M.R. Muggeo, and R. Cusimano, The effect of birthplace on heat tolerance and mortality in Milan, Italy, 1980-1989. International Journal of Biometeorology, 2006. 50(6): p. 335-341.

279. Volney, W.J.A. and R.A. Fleming, Spruce budworm (Choristoneura spp.) biotype reactions to forest and climate characteristics. Global Change Biology, 2007. 13(8): p. 1630-1643.

280. Wake, D.B. and V.T. Vredenburg, Are we in the midst of the sixth mass extinction? A view from the world of amphibians. Proceedings of the National Academy of Sciences of the United States of America, 2008. 105: p. 11466-11473.

281. Walentowski, H. and A. Zehm, Occurrences of relict species and endemics in the temperate deciduous forest region of Bavaria - A geobotanical analysis as a contribution for setting priorities in botanical species conservation. Tuexenia, 2010(30): p. 59-81.

282. Wells, J.C.K., Ecogeographical associations between climate and human body composition: Analyses based on anthropometry and skinfolds. American Journal of Physical Anthropology, 2012. 147(2): p. 169-186.

283. Whitehead, H. and P.J. Richerson, The evolution of conformist social learning can cause population collapse in realistically variable environments. Evolution and Human Behavior, 2009. 30(4): p. 261-273.

284. Wiens, D. and M.R. Slaton, The mechanism of background extinction. Biological Journal of the Linnean Society, 2012. 105(2): p. 255-268.

285. Williams, C.L., et al., Agro-ecoregionalization of Iowa using multivariate geographical clustering. Agriculture Ecosystems & Environment, 2008. 123(1-3): p. 161-174.

286. Williamson, T., H. Hesseln, and M. Johnston, Adaptive capacity deficits and adaptive capacity of economic systems in climate change vulnerability assessment. Forest Policy and Economics, 2012. 15: p. 160-166.

287. Willis, B.L., et al., The role of hybridization in the evolution of reef corals, in Annual Review of Ecology Evolution and Systematics. 2006. p. 489-517.

288. Willis, K.J. and G.M. MacDonald, Long-Term Ecological Records and Their Relevance to Climate Change Predictions for a Warmer World, in Annual Review of Ecology, Evolution, and Systematics, Vol 42, D.J. Futuyma, H.B. Shaffer, and D. Simberloff, Editors. 2011. p. 267-287.

289. Wilson, R.J. and I.M.D. Maclean, Recent evidence for the climate change threat to Lepidoptera and other insects. Journal of Insect Conservation, 2011. 15(1-2): p. 259-268.

290. Winters, G., et al., Spatial and temporal photoacclimation of Stylophora pistillata: zooxanthella size, pigmentation, location and clade. Marine Ecology-Progress Series, 2009. 384: p. 107-119.

291. Wulder, M.A., et al., National circumstances in the international circumboreal community. Forestry Chronicle, 2007. 83(4): p. 539-556.

292. Yang, G.-J., et al., The Regional Network for Asian Schistosomiasis and Other Helminth Zoonoses (RNAS(+)): Target Diseases in Face of Climate Change, in Advances in Parasitology, Vol 73: Important Helminth Infections in Southeast Asia: Diversity and Potential for Control and Elimination, Pt B, X.N. Zhou, et al., Editors. 2010. p. 101-135.

293. Yates, P.M., et al., Diversity in young shark habitats provides the potential for portfolio effects. Marine Ecology-Progress Series, 2012. 458: p. 269-281.

294. Yesson, C. and A. Culham, A phyloclimatic study of Cyclamen. Bmc Evolutionary Biology, 2006. 6.

295. Zheng, H., X. Huang, and K. Butcher, Lithostratigraphy, petrography and facies analysis of the Late Cenozoic sediments in the foreland basin of the West Kunlun. Palaeogeography Palaeoclimatology Palaeoecology, 2006. 241(1): p. 61-78.

**Books, Reviews, Editorials etc (n=153)**

1. Climate Change and Adaptation Strategies for Human Health. Climate Change and Adaptation Strategies for Human Health, ed. B. Menne and K. Ebi. 2006.

2. Climate Change Adaptation in Developed Nations: From Theory to Practice, in Climate Change Adaptation in Developed Nations: From Theory to Practice, J.D. Ford and L. BerrangFord, Editors. 2011.

3. Responding to Climate Change in New York State: The ClimAID Integrated Assessment for Effective Climate Change Adaptation in New York State: Final Report, in Responding to Climate Change in New York State: The ClimAID Integrated Assessment for Effective Climate Change Adaptation in New York State: Final Report, C. Rosenzweig, et al., Editors. 2011.

4. Adanalyan, A. and S. Gevorgyan, The Global Climate Change Impact on Water Resources of Armenia, in Climate Change and Its Effects on Water Resources: Issues of National and Global Security, A. Baba, et al., Editors. 2011. p. 123-129.

5. Adger, W.N., et al., Resilience implications of policy responses to climate change. Wiley Interdisciplinary Reviews-Climate Change, 2011. 2(5): p. 757-766.

6. Adger, W.N., et al., Are there social limits to adaptation to climate change? Climatic Change, 2009. 93(3-4): p. 335-354.

7. Adhikari, B. and K. Taylor, Vulnerability and adaptation to climate change: A review of local actions and national policy response. Climate and Development, 2012. 4(1): p. 54-65.

8. Alston, M., Synthesis paper on socioeconomic factors relating to agriculture and community development. Crop & Pasture Science, 2012. 63(3): p. 232-239.

9. Bassett, E. and V. Shandas, Innovation and Climate Action Planning. Journal of the American Planning Association, 2010. 76(4): p. 435-450.

10. Beeden, R., et al., A Framework for Responding to Coral Disease Outbreaks that Facilitates Adaptive Management. Environmental Management, 2012. 49(1): p. 1-13.

11. Bhopal, R.S. and S.B. Rafnsson, Could mitochondrial efficiency explain the susceptibility to adiposity, metabolic syndrome, diabetes and cardiovascular diseases in South Asian populations? International Journal of Epidemiology, 2009. 38(4): p. 1072-1081.

12. Biazin, B., et al., Rainwater harvesting and management in rainfed agricultural systems in sub-Saharan Africa - A review. Physics and Chemistry of the Earth, 2012. 47-48: p. 139-151.

13. Biggs, D., et al., The implementation crisis in conservation planning: could "mental models" help? Conservation Letters, 2011. 4(3): p. 169-183.

14. Birkmann, J., et al., Adaptive urban governance: new challenges for the second generation of urban adaptation strategies to climate change. Sustainability Science, 2010. 5(2): p. 185-206.

15. Birkmann, J. and K. von Teichman, Integrating disaster risk reduction and climate change adaptation: key challenges-scales, knowledge, and norms. Sustainability Science, 2010. 5(2): p. 171-184.

16. Bloetscher, F., B. Heimlich, and D.E. Meeroff, Development of an adaptation toolbox to protect southeast Florida water supplies from climate change. Environmental Reviews, 2011. 19: p. 397-417.

17. Boyd, E., et al., Exploring Development Futures in a Changing Climate: Frontiers for Development Policy and Practice. Development Policy Review, 2009. 27(6): p. 659-674.

18. Braman, L.M., P. Suarez, and M.K. van Aalst, Climate change adaptation: integrating climate science into humanitarian work. International Review of the Red Cross, 2010. 92(879): p. 693-712.

19. Briceno, S., Investing Today for a Safer Future: How the Hyogo Framework for Action can Contribute to Reducing Deaths During Earthquakes. Earthquake Engineering in Europe, ed. M. Garevski and A. Ansal. Vol. 17. 2010. 441-461.

20. Brown, C., et al., Hydroclimate risk to economic growth in sub-Saharan Africa. Climatic Change, 2011. 106(4): p. 621-647.

21. Brown, K. and E. Westaway, Agency, Capacity, and Resilience to Environmental Change: Lessons from Human Development, Well-Being, and Disasters, in Annual Review of Environment and Resources, Vol 36, A. Gadgil and D.M. Liverman, Editors. 2011. p. 321-342.

22. Brown, S. and G. Walker, Understanding heat wave vulnerability in nursing and residential homes. Building Research and Information, 2008. 36(4): p. 363-372.

23. Burbidge, A.A., et al., Is Australia ready for assisted colonization? Policy changes required to facilitate translocations under climate change. Pacific Conservation Biology, 2011. 17(3, Sp. Iss. SI): p. 259-269.

24. Campbell, S., Ecological specialisation and conservation of Australia's Large-footed Myotis: a review of trawling bat behaviour. Biology and Conservation of Australasian Bats, ed. B. Law, et al. 2011. 72-85.

25. Campoy, J.A., D. Ruiz, and J. Egea, Dormancy in temperate fruit trees in a global warming context: A review. Scientia Horticulturae, 2011. 130(2): p. 357-372.

26. Castleden, M., et al., Resilience thinking in health protection. Journal of Public Health, 2011. 33(3): p. 369-377.

27. Clark, M.S. and L.S. Peck, HSP70 heat shock proteins and environmental stress in Antarctic marine organisms: A mini-review. Marine Genomics, 2009. 2(1): p. 11-18.

28. Conlon, K.C., et al., Preventing cold-related morbidity and mortality in a changing climate. Maturitas, 2011. 69(3): p. 197-202.

29. Corfee-Morlot, J., et al., Multilevel risk governance and urban adaptation policy. Climatic Change, 2011. 104(1): p. 169-197.

30. Crispo, E., et al., Broken barriers: Human-induced changes to gene flow and introgression in animals. Bioessays, 2011. 33(7): p. 508-518.

31. Darnhofer, I., et al., Adaptiveness to enhance the sustainability of farming systems. A review. Agronomy for Sustainable Development, 2010. 30(3): p. 545-555.

32. Darnhofer, I., et al., Adaptiveness to Enhance the Sustainability of Farming Systems. Sustainable Agriculture, Vol 2, ed. E. Lichtfouse, et al. 2011. 45-58.

33. Dixon, M., Climate change, politics and the civil engineering profession. Proceedings of the Institution of Civil Engineers-Municipal Engineer, 2009. 162(4): p. 207-210.

34. Djoudi, H. and M. Brockhaus, Is adaptation to climate change gender neutral? Lessons from communities dependent on livestock and forests in northern Mali. International Forestry Review, 2011. 13(2): p. 123-135.

35. Dodman, D., D. Mitlin, and J.R. Co, Victims to victors, disasters to opportunities Community-driven responses to climate change in the Philippines. International Development Planning Review, 2010. 32(1): p. 1-26.

36. Doulton, H. and K. Brown, Ten years to prevent catastrophe? Discourses of climate change and international development in the UK press. Global Environmental Change-Human and Policy Dimensions, 2009. 19(2): p. 191-202.

37. Drimie, S. and S. Gillespie, Adaptation to climate change in Southern Africa: factoring in AIDS. Environmental Science & Policy, 2010. 13(8): p. 778-784.

38. Dumaru, P., Community-based adaptation: enhancing community adaptive capacity in Druadrua Island, Fiji. Wiley Interdisciplinary Reviews-Climate Change, 2010. 1(5): p. 751-763.

39. Eisenack, K., et al., Adaptation to climate change in the transport sector: a review of actions and actors. Mitigation and Adaptation Strategies for Global Change, 2012. 17(5): p. 451-469.

40. English, P.B., et al., Environmental Health Indicators of Climate Change for the United States: Findings from the State Environmental Health Indicator Collaborative. Environmental Health Perspectives, 2009. 117(11): p. 1673-1681.

41. Feehan, J., M. Harley, and J. van Minnen, Climate change in Europe. 1. Impact on terrestrial ecosystems and biodiversity. A review (Reprinted). Agronomy for Sustainable Development, 2009. 29(3): p. 409-421.

42. Ford, J.D., Indigenous Health and Climate Change. American Journal of Public Health, 2012. 102(7): p. 1260-1266.

43. Ford, J.D., L. Berrang-Ford, and J. Paterson, A systematic review of observed climate change adaptation in developed nations A letter. Climatic Change, 2011. 106(2): p. 327-336.

44. Ford, J.D. and C. Goldhar, Climate change vulnerability and adaptation in resource dependent communities: a case study from West Greenland. Climate Research, 2012. 54(2): p. 181-196.

45. Ford, J.D., et al., Case study and analogue methodologies in climate change vulnerability research. Wiley Interdisciplinary Reviews-Climate Change, 2010. 1(3): p. 374-392.

46. Ford, J.D. and T. Pearce, What we know, do not know, and need to know about climate change vulnerability in the western Canadian Arctic: a systematic literature review. Environmental Research Letters, 2010. 5(1).

47. Ford, J.D., et al., Climate change policy responses for Canada's Inuit population: The importance of and opportunities for adaptation. Global Environmental Change-Human and Policy Dimensions, 2010. 20(1): p. 177-191.

48. Fraser, E.D.G., et al., Assessing Vulnerability to Climate Change in Dryland Livelihood Systems: Conceptual Challenges and Interdisciplinary Solutions. Ecology and Society, 2011. 16(3).

49. Fuessel, H.-M., Vulnerability: A generally applicable conceptual framework for climate change research. Global Environmental Change-Human and Policy Dimensions, 2007. 17(2): p. 155-167.

50. Fuessel, H.-M., Assessing adaptation to the health risks of climate change: what guidance can existing frameworks provide? International Journal of Environmental Health Research, 2008. 18(1): p. 37-63.

51. Galvin, K.A., Transitions: Pastoralists Living with Change, in Annual Review of Anthropology. 2009. p. 185-198.

52. Garrity, D.P., et al., Evergreen Agriculture: a robust approach to sustainable food security in Africa. Food Security, 2010. 2(3): p. 197-214.

53. Garschagen, M., F.G. Renaud, and J. Birkmann, Dynamic Resilience of Peri-Urban Agriculturalists in the Mekong Delta Under Pressures of Socio-Economic Transformation and Climate Change, in Environmental Change and Agricultural Sustainability in the Mekong Delta, M.A. Stewart and P.A. Coclanis, Editors. 2011. p. 141-163.

54. Gessler, A., et al., Potential risks for European beech (Fagus sylvatica L.) in a changing climate. Trees-Structure and Function, 2007. 21(1): p. 1-11.

55. Glenk, K. and A. Fischer, Insurance, prevention or just wait and see? Public preferences for water management strategies in the context of climate change. Ecological Economics, 2010. 69(11): p. 2279-2291.

56. Gupta, R. and M. Gregg, Using UK climate change projections to adapt existing English homes for a warming climate. Building and Environment, 2012. 55: p. 20-42.

57. Hahn, W.A. and T. Knoke, Sustainable development and sustainable forestry: analogies, differences, and the role of flexibility. European Journal of Forest Research, 2010. 129(5): p. 787-801.

58. Hajat, S., M. O'Connor, and T. Kosatsky, Health effects of hot weather: from awareness of risk factors to effective health protection. Lancet, 2010. 375(9717): p. 856-863.

59. Hamisi, H.I., et al., Crisis in the wetlands: Combined stresses in a changing climate - Experience from Tanzania. Climate and Development, 2012. 4(1): p. 5-15.

60. Hansen, A., et al., Older persons and heat-susceptibility: the role of health promotion in a changing climate. Health Promotion Journal of Australia, 2011. 22: p. S17-S20.

61. Hansen, A., et al., Perceptions of Heat-Susceptibility in Older Persons: Barriers to Adaptation. International Journal of Environmental Research and Public Health, 2011. 8(12): p. 4714-4728.

62. Hardoy, J. and P.R. Lankao, Latin American cities and climate change: challenges and options to mitigation and adaptation responses. Current Opinion in Environmental Sustainability, 2011. 3(3): p. 158-163.

63. Hecht, S.B., Climate change and the transformation of risk: Insurance matters. Ucla Law Review, 2008. 55(6): p. 1559-1620.

64. Heino, J., R. Virkkala, and H. Toivonen, Climate change and freshwater biodiversity: detected patterns, future trends and adaptations in northern regions. Biological Reviews, 2009. 84(1): p. 39-54.

65. Hess, J.J., J.N. Malilay, and A.J. Parkinson, Climate Change The Importance of Place. American Journal of Preventive Medicine, 2008. 35(5): p. 468-478.

66. Hewitt, N., et al., Taking stock of the assisted migration debate. Biological Conservation, 2011. 144(11): p. 2560-2572.

67. Hofmann, G.E., et al., The Effect of Ocean Acidification on Calcifying Organisms in Marine Ecosystems: An Organism-to-Ecosystem Perspective, in Annual Review of Ecology, Evolution, and Systematics, Vol 41, D.J. Futuyma, H.B. Shafer, and D. Simberloff, Editors. 2010. p. 127-147.

68. Hosking, J. and D. Campbell-Lendrum, How well does climate change and human health research match the demands of policymakers? A scoping review. Environmental Health Perspectives, 2012. 120(8): p. 1076-82.

69. Huang, C., et al., Constraints and Barriers to Public Health Adaptation to Climate Change A Review of the Literature. American Journal of Preventive Medicine, 2011. 40(2): p. 183-190.

70. Hultman, N.E., D.M. Hassenzahl, and S. Rayner, Climate Risk, in Annual Review of Environment and Resources, Vol 35, A. Gadgil and D.M. Liverman, Editors. 2010. p. 283-303.

71. Hutton, D., Behavioral Health and Risk Perception: Factors in Strengthening Community Resiliency and Emergency Preparedness, in Climate Change Adaptation in Developed Nations: From Theory to Practice, J.D. Ford and L. BerrangFord, Editors. 2011. p. 133-142.

72. Jacobsen, S.E., C.R. Jensen, and F. Liu, Improving crop production in the arid Mediterranean climate. Field Crops Research, 2012. 128: p. 34-47.

73. Jarvis, A., et al., AN INTEGRATED ADAPTATION AND MITIGATION FRAMEWORK FOR DEVELOPING AGRICULTURAL RESEARCH: SYNERGIES AND TRADE-OFFS. Experimental Agriculture, 2011. 47(2): p. 185-203.

74. Jiang, L. and K. Hardee, How do Recent Population Trends Matter to Climate Change? Population Research and Policy Review, 2011. 30(2): p. 287-312.

75. Johnson, C.A. and K. Krishnamurthy, Dealing with displacement: Can "social protection" facilitate long-term adaptation to climate change? Global Environmental Change-Human and Policy Dimensions, 2010. 20(4): p. 648-655.

76. Johnson, J.E. and D.J. Welch, Marine Fisheries Management in a Changing Climate: A Review of Vulnerability and Future Options. Reviews in Fisheries Science, 2010. 18(1): p. 106-124.

77. Kallis, G., Droughts, in Annual Review of Environment and Resources. 2008. p. 85-118.

78. Kampragou, E., et al., Towards the harmonization of water-related policies for managing drought risks across the EU. Environmental Science & Policy, 2011. 14(7): p. 815-824.

79. Kenny, G.P., et al., Heat stress in older individuals and patients with common chronic diseases. Canadian Medical Association Journal, 2010. 182(10): p. 1053-1060.

80. Kjellstrom, T., et al., The 'Hothaps' programme for assessing climate change impacts on occupational health and productivity: an invitation to carry out field studies. Global health action, 2009. 2: p. 81-87.

81. Knudsen, E., et al., Challenging claims in the study of migratory birds and climate change. Biological Reviews, 2011. 86(4): p. 928-946.

82. Krishnan, R., et al., HIGH-TEMPERATURE EFFECTS ON RICE GROWTH, YIELD, AND GRAIN QUALITY, in Advances in Agronomy, Vol 111, D.L. Sparks, Editor. 2011. p. 87-206.

83. Kwiatkowski, R.E., Indigenous community based participatory research and health impact assessment: A Canadian example. Environmental Impact Assessment Review, 2011. 31(4): p. 445-450.

84. Leimu, R., et al., Habitat fragmentation, climate change, and inbreeding in plants, in Year in Ecology and Conservation Biology 2010, R.S. Ostfeld and W.H. Schlesinger, Editors. 2010. p. 84-98.

85. Li, Y., A. Ahuja, and J.E. Padgett, Review of Methods to Assess, Design for, and Mitigate Multiple Hazards. Journal of Performance of Constructed Facilities, 2012. 26(1): p. 104-117.

86. Lindgren, E., A. Albihn, and Y. Andersson, Climate Change, Water-Related Health Impacts, and Adaptation: Highlights from the Swedish Government's Commission on Climate and Vulnerability, in Climate Change Adaptation in Developed Nations: From Theory to Practice, J.D. Ford and L. BerrangFord, Editors. 2011. p. 177-188.

87. Liverman, D., Assessing impacts, adaptation and vulnerability: Reflections on the Working Group II Report of the Intergovernmental Panel on Climate Change. Global Environmental Change-Human and Policy Dimensions, 2008. 18(1): p. 4-7.

88. Lopez-Larrosa, S., The family system upon divorce: Risk and protective factors and intervention programmes. Cultura Y Educacion, 2009. 21(4): p. 391-402.

89. Mable, B.K., M.A. Alexandrou, and M.I. Taylor, Genome duplication in amphibians and fish: an extended synthesis. Journal of Zoology, 2011. 284(3): p. 151-182.

90. Manderson, L. and T. Victoria, Social and public health effects of climate change in the '40 South'. Wiley Interdisciplinary Reviews-Climate Change, 2011. 2(6): p. 902-918.

91. Mathot, K.J., et al., Adaptive strategies for managing uncertainty may explain personality-related differences in behavioural plasticity. Oikos, 2012. 121(7): p. 1009-1020.

92. Mavrogianni, A., et al., LONDON HOUSING AND CLIMATE CHANGE: Impact on Comfort and Health - Preliminary Results of a Summer Overheating Study. Open House International, 2010. 35(2): p. 49-59.

93. Mawdsley, J.R., R. O'Malley, and D.S. Ojima, A Review of Climate-Change Adaptation Strategies for Wildlife Management and Biodiversity Conservation. Conservation Biology, 2009. 23(5): p. 1080-1089.

94. McBean, G. and C. Rodgers, Climate hazards and disasters: the need for capacity building. Wiley Interdisciplinary Reviews-Climate Change, 2010. 1(6): p. 871-884.

95. McLeman, R. and B. Smit, Vulnerability to climate change hazards and risks: crop and flood insurance. Canadian Geographer-Geographe Canadien, 2006. 50(2): p. 217-226.

96. McLeman, R.A., et al., Opportunities and Barriers for Adaptation and Local Adaptation Planning in Canadian Rural and Resource-Based Communities, in Climate Change Adaptation in Developed Nations: From Theory to Practice, J.D. Ford and L. BerrangFord, Editors. 2011. p. 449-459.

97. McMichael, C., J. Barnett, and A.J. McMichael, An Ill Wind? Climate Change, Migration, and Health. Environmental Health Perspectives, 2012. 120(5): p. 646-654.

98. Meera, S.N., et al., Changing Roles of Agricultural Extension: Harnessing Information and Communication Technology (ICT) for Adapting to Stresses Envisaged Under Climate Change. Crop Stress and its Management: Perspectives and Strategies, ed. B. Venkateswarlu, et al. 2012. 585-605.

99. Molua, E.L., Accommodation of climate change in coastal areas of Cameroon: selection of household-level protection options. Mitigation and Adaptation Strategies for Global Change, 2009. 14(8): p. 721-735.

100. Morris, M.J., E.S. Na, and A.K. Johnson, Salt craving: The psychobiology of pathogenic sodium intake. Physiology & Behavior, 2008. 94(5): p. 709-721.

101. Morss, R.E., et al., Improving Societal Outcomes of Extreme Weather in a Changing Climate: An Integrated Perspective, in Annual Review of Environment and Resources, Vol 36, A. Gadgil and D.M. Liverman, Editors. 2011. p. 1-25.

102. Moss, R.H., et al., The next generation of scenarios for climate change research and assessment. Nature, 2010. 463(7282): p. 747-756.

103. Motsholapheko, M.R., D.L. Kgathi, and C. Vanderpost, Rural livelihoods and household adaptation to extreme flooding in the Okavango Delta, Botswana. Physics and Chemistry of the Earth, 2011. 36(14-15): p. 984-995.

104. Nardone, A., et al., Effects of climate changes on animal production and sustainability of livestock systems. Livestock Science, 2010. 130(1-3): p. 57-69.

105. Nelson, D.R., Adaptation and resilience: responding to a changing climate. Wiley Interdisciplinary Reviews-Climate Change, 2011. 2(1): p. 113-120.

106. Newton, A.C., et al., Cereal landraces for sustainable agriculture. A review. Agronomy for Sustainable Development, 2010. 30(2): p. 237-269.

107. Newton, A.C., et al., Climate Change and Defense against Pathogens in Plants. Advances in applied microbiology, 2012. 81: p. 89-132.

108. Noss, R.F., Between the devil and the deep blue sea: Florida's unenviable position with respect to sea level rise. Climatic Change, 2011. 107(1-2): p. 1-16.

109. Ogden, N.H., P. Sockett, and M. Fleury, Public Health in Canada and Adaptation to Infectious Disease Risks of Climate Change: Are We Planning or Just Keeping Our Fingers Crossed?, in Climate Change Adaptation in Developed Nations: From Theory to Practice, J.D. Ford and L. BerrangFord, Editors. 2011. p. 161-175.

110. Olsson, L. and A. Jerneck, Farmers fighting climate change-from victims to agents in subsistence livelihoods. Wiley Interdisciplinary Reviews-Climate Change, 2010. 1(3): p. 363-373.

111. Ooi, M.K.J., Seed bank persistence and climate change. Seed Science Research, 2012. 22: p. S53-S60.

112. Orlandini, S., et al., Impacts of Climate Change and Variability on European Agriculture Results of Inventory Analysis in COST 734 Countries, in Trends and Directions in Climate Research, L. Gimeno, R. GarciaHerrera, and R.M. Trigo, Editors. 2008. p. 338-353.

113. Oven, K.J., et al., Climate change and health and social care: Defining future hazard, vulnerability and risk for infrastructure systems supporting older people's health care in England. Applied Geography, 2012. 33(1): p. 16-24.

114. Overbeck, M. and M. Schmidt, Modelling infestation risk of Norway spruce by Ips typographus (L.) in the Lower Saxon Harz Mountains (Germany). Forest Ecology and Management, 2012. 266: p. 115-125.

115. Pascal, M., et al., How can a climate change perspective be integrated into public health surveillance? Public Health, 2012. 126(8): p. 660-667.

116. Pearce, T., et al., Advancing adaptation planning for climate change in the Inuvialuit Settlement Region (ISR): a review and critique. Regional Environmental Change, 2011. 11(1): p. 1-17.

117. Plaganyi, E.E., et al., Modelling climate-change effects on Australian and Pacific aquatic ecosystems: a review of analytical tools and management implications. Marine and Freshwater Research, 2011. 62(9): p. 1132-1147.

118. Prabhakar, S.V.R.K. and R. Shaw, Climate change adaptation implications for drought risk mitigation: a perspective for India. Climatic Change, 2008. 88(2): p. 113-130.

119. Rawlani, A.K. and B.K. Sovacool, Building responsiveness to climate change through community based adaptation in Bangladesh. Mitigation and Adaptation Strategies for Global Change, 2011. 16(8): p. 845-863.

120. Reif, A., et al., Forest Management in Times of Climate Change - Synergies and potential conflicts between forestry and nature conservation. Naturschutz und Landschaftsplanung, 2010. 42(9): p. 261-266.

121. Richardson, M.J., P. English, and L. Rudolph, A health impact assessment of California's proposed cap-and-trade regulations. American Journal of Public Health, 2012. 102(9): p. e52-8.

122. Rocklov, J., K. Ebi, and B. Forsberg, Mortality related to temperature and persistent extreme temperatures: a study of cause-specific and age-stratified mortality. Occupational and Environmental Medicine, 2011. 68(7): p. 531-536.

123. Satterthwaite, D., Editorial: Why is community action needed for disaster risk reduction and climate change adaptation? Environment and Urbanization, 2011. 23(2): p. 339-349.

124. Semenza, J.C., Lateral Public Health: A Comprehensive Approach to Adaptation in Urban Environments, in Climate Change Adaptation in Developed Nations: From Theory to Practice, J.D. Ford and L. BerrangFord, Editors. 2011. p. 143-159.

125. Simberloff, D., The Role of Propagule Pressure in Biological Invasions, in Annual Review of Ecology Evolution and Systematics. 2009. p. 81-102.

126. Smit, B. and J. Wandel, Adaptation, adaptive capacity and vulnerability. Global Environmental Change-Human and Policy Dimensions, 2006. 16(3): p. 282-292.

127. Smith, W., B. Grant, and R. Desjardins, Some perspectives on agricultural GHG mitigation and adaptation strategies with respect to the impact of climate change/variability in vulnerable areas. Idojaras, 2009. 113(1-2): p. 103-115.

128. Snell-Rood, E.C., Selective Processes in Development: Implications for the Costs and Benefits of Phenotypic Plasticity. Integrative and Comparative Biology, 2012. 52(1): p. 31-42.

129. Stage, J., Economic valuation of climate change adaptation in developing countries, in Ecological Economics Reviews, K. Limburg and R. Costanza, Editors. 2010. p. 150-163.

130. Strand, L.B., et al., Vulnerability of eco-environmental health to climate change: the views of government stakeholders and other specialists in Queensland, Australia. Bmc Public Health, 2010. 10.

131. Sutherland, W.J., Predicting the ecological consequences of environmental change: a review of the methods. Journal of Applied Ecology, 2006. 43(4): p. 599-616.

132. Thiel, M., et al., The Humboldt Current System of northern and central Chile, in Oceanography and Marine Biology, Vol 45, R.N. Gibson, R.J.A. Atkinson, and J.D.M. Gordon, Editors. 2007. p. 195-344.

133. Tompkins, E.L. and H. Eakin, Managing private and public adaptation to climate change. Global Environmental Change-Human and Policy Dimensions, 2012. 22(1): p. 3-11.

134. Troccoli, WEATHER/CLIMATE RISK MANAGEMENT FOR THE ENERGY SECTOR: WORKSHOP RECOMMENDATIONS, in Management of Weather and Climate Risk in the Energy Industry, A. Troccoli, Editor. 2010. p. 327-332.

135. Troccoli, A., Seasonal climate forecasting. Meteorological Applications, 2010. 17(3): p. 251-268.

136. Tscharntke, T., et al., Multifunctional shade-tree management in tropical agroforestry landscapes - a review. Journal of Applied Ecology, 2011. 48(3): p. 619-629.

137. van Aalst, M.K., T. Cannon, and I. Burton, Community level adaptation to climate change: The potential role of participatory community risk assessment. Global Environmental Change-Human and Policy Dimensions, 2008. 18(1): p. 165-179.

138. van Pelt, S.C. and R.J. Swart, Climate Change Risk Management in Transnational River Basins: The Rhine. Water Resources Management, 2011. 25(14): p. 3837-3861.

139. Vegas-Vilarrubia, T., et al., Quaternary palaeoecology and nature conservation: a general review with examples from the neotropics. Quaternary Science Reviews, 2011. 30(19-20): p. 2361-2388.

140. Vogel, S., Leaves in the lowest and highest winds: temperature, force and shape. New Phytologist, 2009. 183(1): p. 13-26.

141. Walentowski, H. and A. Zehm, Occurrences of relict species and endemics in the temperate deciduous forest region of Bavaria - A geobotanical analysis as a contribution for setting priorities in botanical species conservation. Tuexenia, 2010(30): p. 59-81.

142. Walker, R., et al., Health promotion interventions to address climate change using a primary health care approach: a literature review. Health Promotion Journal of Australia, 2011. 22: p. S6-S12.

143. Wassmann, R., et al., CLIMATE CHANGE AFFECTING RICE PRODUCTION: THE PHYSIOLOGICAL AND AGRONOMIC BASIS FOR POSSIBLE ADAPTATION STRATEGIES, in Advances in Agronomy, Vol 101, D.L. Sparks, Editor. 2009. p. 59-122.

144. White-Newsome, J.L., et al., Assessing heat-adaptive behaviors among older, urban-dwelling adults. Maturitas, 2011. 70(1): p. 85-91.

145. Wilby, R.L. and R. Keenan, Adapting to flood risk under climate change. Progress in Physical Geography, 2012. 36(3): p. 348-378.

146. Wilby, R.L., et al., A review of climate risk information for adaptation and development planning. International Journal of Climatology, 2009. 29(9): p. 1193-1215.

147. Willis, B.L., et al., The role of hybridization in the evolution of reef corals, in Annual Review of Ecology Evolution and Systematics. 2006. p. 489-517.

148. Willis, K.J. and G.M. MacDonald, Long-Term Ecological Records and Their Relevance to Climate Change Predictions for a Warmer World, in Annual Review of Ecology, Evolution, and Systematics, Vol 42, D.J. Futuyma, H.B. Shaffer, and D. Simberloff, Editors. 2011. p. 267-287.

149. Wilson, R.J. and I.M.D. Maclean, Recent evidence for the climate change threat to Lepidoptera and other insects. Journal of Insect Conservation, 2011. 15(1-2): p. 259-268.

150. Wong, P.P., Small island developing states. Wiley Interdisciplinary Reviews-Climate Change, 2011. 2(1): p. 1-6.

151. Zamin, T.J., et al., National Red Listing Beyond the 2010 Target. Conservation Biology, 2010. 24(4): p. 1012-1020.

152. Ziervogel, G. and P.J. Ericksen, Adapting to climate change to sustain food security. Wiley Interdisciplinary Reviews-Climate Change, 2010. 1(4): p. 525-540.

153. Zimmerer, K.S., Biological Diversity in Agriculture and Global Change, in Annual Review of Environment and Resources, Vol 35, A. Gadgil and D.M. Liverman, Editors. 2010. p. 137-+.
